# Identification of integrated proteomics and transcriptomics signature of alcohol-associated liver disease using machine learning

**DOI:** 10.1371/journal.pdig.0000447

**Published:** 2024-02-09

**Authors:** Stanislav Listopad, Christophe Magnan, Le Z. Day, Aliya Asghar, Andrew Stolz, John A. Tayek, Zhang-Xu Liu, Jon M. Jacobs, Timothy R. Morgan, Trina M. Norden-Krichmar

**Affiliations:** 1 Department of Computer Science, University of California, Irvine, California, United States of America; 2 Biological Sciences Division and Environmental and Molecular Sciences Division, Pacific Northwest National Laboratory, Richland, Washington, United States of America; 3 Medical and Research Services, VA Long Beach Healthcare System, Long Beach, California, United States of America; 4 Division of Gastrointestinal & Liver Diseases, Department of Medicine, Keck School of Medicine, University of Southern California, Los Angeles, California, United States of America; 5 Lundquist Institute for Biomedical Innovation at Harbor-UCLA Medical Center, Department of Internal Medicine, David Geffen School of Medicine, University of California Los Angeles, Torrance, California, United States of America; 6 Department of Epidemiology and Biostatistics, University of California, Irvine, California, United States of America; McGill University, CANADA

## Abstract

Distinguishing between alcohol-associated hepatitis (AH) and alcohol-associated cirrhosis (AC) remains a diagnostic challenge. In this study, we used machine learning with transcriptomics and proteomics data from liver tissue and peripheral mononuclear blood cells (PBMCs) to classify patients with alcohol-associated liver disease. The conditions in the study were AH, AC, and healthy controls. We processed 98 PBMC RNAseq samples, 55 PBMC proteomic samples, 48 liver RNAseq samples, and 53 liver proteomic samples. First, we built separate classification and feature selection pipelines for transcriptomics and proteomics data. The liver tissue models were validated in independent liver tissue datasets. Next, we built integrated gene and protein expression models that allowed us to identify combined gene-protein biomarker panels. For liver tissue, we attained 90% nested-cross validation accuracy in our dataset and 82% accuracy in the independent validation dataset using transcriptomic data. We attained 100% nested-cross validation accuracy in our dataset and 61% accuracy in the independent validation dataset using proteomic data. For PBMCs, we attained 83% and 89% accuracy with transcriptomic and proteomic data, respectively. The integration of the two data types resulted in improved classification accuracy for PBMCs, but not liver tissue. We also identified the following gene-protein matches within the gene-protein biomarker panels: *CLEC4M-CLC4M*, *GSTA1-GSTA2* for liver tissue and *SELENBP1-SBP1* for PBMCs. In this study, machine learning models had high classification accuracy for both transcriptomics and proteomics data, across liver tissue and PBMCs. The integration of transcriptomics and proteomics into a multi-omics model yielded improvement in classification accuracy for the PBMC data. The set of integrated gene-protein biomarkers for PBMCs show promise toward developing a liquid biopsy for alcohol-associated liver disease.

## Author summary

Alcohol-associated cirrhosis and alcohol-associated hepatitis can be difficult to classify clinically. Previously, we established that these two diseases can be differentiated using RNA sequencing gene expression data collected from either liver tissue biopsies or from peripheral blood mononuclear cells (PBMCs), which are extracted from blood samples. In the current study, we investigated whether using protein expression data, in addition to gene expression data, would improve our machine learning models’ ability to distinguish between the two alcohol-associated liver diseases and enable identification of gene and protein biomarkers. We found that our models accurately classified alcohol-associated liver diseases with each data type. We were also able to identify promising tissue and blood-based diagnostic gene and protein biomarkers. Additionally, we have demonstrated that challenges present in analyzing small sample size, high dimensional genomic data can be addressed through careful application of appropriate software, bioinformatics, and machine learning methods. By applying these computational approaches to this liver disease genomics data set, we have identified blood-based diagnostic biomarkers of liver disease that will potentially contribute to the development of highly accurate blood tests that will replace invasive liver biopsies.

## Introduction

In this study, we focused on alcohol-associated hepatitis (AH) and alcohol-associated cirrhosis (AC) because these are deadly liver conditions with similar clinical presentation. In 2019 there were 23,780 deaths from alcohol-associated cirrhosis (AC) in United States [1]. This is more than triple the number of deaths from alcohol-associated cirrhosis in 1999. The patients with alcohol-associated liver disease (ALD) account for 18% of liver transplants [2]. However, attaining a liver transplant as an ALD patient is difficult, since donor livers are scarce and there are concerns about allocation to individuals with alcohol addiction [2]. Typically, a 6-month abstinence from alcohol is required to be a candidate for liver transplant [2]. Many of ALD patients have alcohol-associated hepatitis (AH) a condition which carries mortality as high as 50% at 3 months [3]. For the severe AH patients, the 6-month abstinence requirement can be tantamount to a death sentence [2]. When carefully selected, ALD patients can benefit from liver transplantation [4,5,6,7]. Currently, establishing AH diagnosis can require liver biopsy, typically done using a transjugular route [3]. Liver biopsy has several limitations, such as procedural risk of internal bleeding, high cost, and patient dissatisfaction. Thus, development of a non-invasive test that can reliably distinguish between AH and AC would be beneficial. Currently, there are a large number of imaging and blood tests for diagnosis of liver cirrhosis [8]. However, liver biopsy remains the current standard for diagnosis [9]. Further improvement in accuracy of non-invasive tests is necessary to reduce the need for liver biopsy [10].

In a previous study, we established that gene expression biomarkers from liver tissue and peripheral mononuclear blood cells (PBMCs) can be used with a multiclass machine learning approach to successfully distinguish between multiple liver diseases [11]. In the present study, in addition to transcriptomic data, we also obtained proteomic data for participants from the same cohort [12]. Addition of proteomic data presented new opportunities, but it also further increased the ratio of feature size to sample size. This made overfitting a greater challenge than when we only used the gene expression data. First, we compared how well gene and protein biomarkers could be used to classify these conditions separately. Then we examined whether further improvement in classification accuracy could be obtained by combining transcriptomic and proteomic data. As part of the classification process, we have identified the most effective gene and protein biomarkers of alcohol-associated liver disease. We also examined the degree of concordance between top differentially expressed proteins and genes for the three conditions. The gene and protein biomarkers identified in this study, with further validation, could be used to develop new highly accurate blood tests to distinguish between various types of ALD.

## Materials and methods

### Study population

This study was primarily conducted using biospecimens collected from participants enrolled by the Southern California Alcoholic Hepatitis Consortium (SCAHC). The protocol was approved by the IRB, and informed written consent was obtained from all participants. The liver tissue from participants with AC and healthy controls were obtained from the liver tissue cell distribution system (LTCDS) at University of Minnesota. The study population demographics for liver tissue and PBMC samples for transcriptomic and proteomic analyses can be found in Tables 1 and 2.

**Table 1:** Study population demographics (liver) for proteomic and RNAseq analysis.

	Liver tissue samples (proteomics)			Liver tissue samples (transcriptomics)		
	AH	CT	AC	AH	CT	AC
	n = 33	n = 10	n = 10	n = 32	n = 8	n = 8
**Age: mean ± std**	42.7 ± 11.4	56 ± 8.6	51.9 ± 13.1	43.3 ± 11.3	55.4 ± 4.3	54.2 ± 6.9*
**MELD: mean ± std**	25.2 ± 5.7	NA	32 ± 6.1*	25.1 ± 5.7	NA	NA
**Maddrey’s DF: mean**	53.3 ± 22.2	NA	NA	52.3 ± 22.1	NA	NA
**BMI: mean ± std**	29 ± 5.3	NA	25.6 ± 8.4*	29.4 ± 5.9	NA	NA
** **						
**Gender: N (percent)**						
Female	3(9.1%)	0(0.0%)	0(0.0%)	3(9.4%)	0(0.0%)	0(0.0%)
Male	30(90.9%)	10(100%)	9(90%)	29(90.6%)	7(87.5%)	5(62.5%)
** **						
**Ethnicity: N (percent)**						
Hispanic	25(75.8%)	NA	0(0.0%)	25 (78.1%)	NA	0 (0.0%)
NHW	5(15.1%)	NA	5(50%)	5 (15.6%)	NA	4 (50.0%)
Black	2(6.1%)	NA	0(0.0%)	1 (3.1%)	NA	0 (0.0%)
Other	1(3.0%)	NA	0(0.0%)	1 (3.1%)	NA	0 (0.0%)
Source	SCAHC	LTCDS	LTCDS	SCAHC	LTCDS	LTCDS

Abbreviations: AC, alcohol-associated cirrhosis; AH, alcohol-associated hepatitis; CT, healthy controls; MELD, model for end-stage liver disease; NHW, non-Hispanic White; NA, not available; SCAHC, Southern California Alcoholic Hepatitis Consortium.

*Missing MELD scores for 7 proteomic AC samples, BMI for 8 proteomic AC samples, and age for 3 transcriptomic AC samples.

**Table 2:** Study population demographics (PBMCs) for proteomic and RNAseq analysis.

	PBMC samples (proteomics)			PBMC samples (transcriptomics)		
	AH	CT	AC	AH	CT	AC
	n = 20	n = 22	n = 13	n = 38	n = 20	n = 40
**Age: mean ± std**	48.7 ± 11.6	34.8 ± 15.1	54.2 ± 11.2	47.3 ± 11.5	35.9 ± 15.6	54.5 ± 9.7
**MELD: mean ± std**	24.5 ± 3.6	7.5 ± 2.5	13.6 ± 6.7	25 ± 3.8	7.3 ± 2.6	13.4 ± 5.8
**Maddrey’s DF: mean**	49.3 ± 17.3	2.5 ± 7.8	22.1 ± 23.3	52.6 ± 20.7	2.4 ± 8.1	21.1 ± 19.1
**BMI: mean ± std**	29.6 ± 5.5	27.1 ± 4	30 ± 4.8	30 ± 6.2	27 ± 3.5	30.4 ± 5.1
** **						
**Gender: N (percent)**						
Female	1(5%)	10(45.4%)	0(0.0%)	1 (2.6%)	8 (40.0%)	0 (0.0%)
Male	19(95%)	12(54.6%)	13(100%)	37 (97.4%)	12 (60.0%)	40(100.0%)
** **						
**Ethnicity: N (percent)**						
Hispanic	12(60%)	12(54.5%)	10(76.9%)	25 (65.8%)	8 (40.0%)	25 (62.5%)
NHW	5(25%)	0(0.0%)	2(15.4%)	10 (26.3%)	0 (0.0%)	13 (32.5%)
Black	2(10%)	1(4.5%)	0(0.0%)	2 (5.3%)	2 (10.0%)	1 (2.5%)
Other	1(5%)	12(54.5%)	1(7.7%)	1 (2.6%)	10 (50.0%)	1 (2.5%)
Source	SCAHC	SCAHC	SCAHC	SCAHC	SCAHC	SCAHC

*The ethnicity and sex percentages may not add up to 100% due to missing data.

Abbreviations: AC, alcohol-associated cirrhosis; AH, alcohol-associated hepatitis; CT, healthy controls; LTCDS, Liver Tissue Cell Distribution System; MELD, model for end-stage liver disease; NHW, non-Hispanic White; NA, not available; SCAHC, Southern California Alcoholic Hepatitis Consortium.

The biospecimens consisted of 98 PBMC RNAseq samples, 55 PBMC proteomic samples, 48 liver tissue RNAseq samples, and 53 liver tissue proteomic samples. The liver diseases represented were encoded with two letter symbols as follows: alcohol-associated hepatitis (AH) and alcohol-associated cirrhosis (AC). Most of the AC participants within the SCAHC study were expected to be in-patients with decompensated cirrhosis. Best efforts were made during recruitment of the AH and AC groups within SCAHC study to match based on age, gender, and ethnicity. Severity-based matching was not possible due to small sample size. One of the main reasons for small sample size in our study and in publicly available data sets, is difficulty in recruiting patients with AH. AH has a low incidence rate of an estimated 4.5 hospitalizations per 100,000 person per year [13]. Additional information about the inclusion and exclusion criteria, sample collection, sample processing, and preliminary data processing can be found in S1 Text.

### Partitioning samples into datasets

Because some proteomic and transcriptomic samples came from the same participants, while others did not, we implemented a strategy to partition and balance the samples in the datasets into matched and unmatched sets. Table 3 summarizes the degree of matching between proteomic and transcriptomic samples in liver tissue and PBMC. For several algorithms in the pipeline, some of the unmatched subsets were too small. Therefore, we moved some matched samples into unmatched sample categories, and we will refer to these new categories as “balanced matched” and “balanced unmatched” subsets. We divided our data into the following dataset categories described below.

**Table 3:** The degree of matching between proteomic and transcriptomic samples for PBMC and liver tissue. The numbers in parenthesis denote the number of samples that were moved from matched category into matched balanced and unmatched balanced categories.

	PBMC (proteomics)			PBMC (transcriptomics)		
	AH	CT	AC	AH	CT	AC
Full	20	22	13	38	20	40
Matched	18	19	13	18	19	13
Unmatched	2	3	0	20	1	27
Matched Balanced	9(-9)	12(-7)	6(-7)	9(-9)	12(-7)	6(-7)
Unmatched Balanced	11(+9)	10(+7)	7(+7)	29(+9)	8(+7)	34(+7)
	Liver (proteomics)			Liver (transcriptomics)		
	AH	CT	AC	AH	CT	AC
Full	33	10	10	32	8	8
Matched	29	3	5	29	3	5
Unmatched	4	7	5	3	5	3
Matched Balanced	24(-5)	3	3(-2)	24(-5)	3	3(-2)
Unmatched Balanced	9(+5)	7	7(+2)	8(+5)	5	5(+2)

#### Full datasets

These datasets are composed of all available samples for the given tissue and genomic datatype: PBMC 3-Way Full proteomics, PBMC 3-Way Full RNAseq, Liver 3-Way Full proteomics, and Liver 3-Way Full RNAseq.

#### Unmatched balanced datasets

These datasets consist of a mixture of matched and unmatched samples: PBMC 3-Way Unmatched Balanced proteomics, PBMC 3-Way Unmatched Balanced RNAseq, Liver 3-Way Unmatched Balanced proteomics, and Liver 3-Way Unmatched Balanced RNAseq.

#### Matched balanced datasets

These datasets consist of only matched samples, such that for each RNAseq sample there is also a proteomic sample obtained from the same individual: PBMC 3-Way Matched Balanced proteomics, PBMC 3-Way Matched Balanced RNAseq, Liver 3-Way Matched Balanced proteomics, and Liver 3-Way Matched Balanced RNAseq.

#### Matched balanced integrated datasets

These datasets were formed by merging the proteomic and RNAseq data from Matched Balanced datasets: PBMC 3-Way Matched Balanced Integrated and Liver 3-Way Matched Balanced Integrated.

### Validation dataset

We validated our proteomic liver tissue machine learning (ML) models using data obtained from MassIVE repository (accession number MSV000089168) [12]. This dataset contained liver tissue proteomic data from participants with AH (n = 6) and healthy controls (n = 12). Notably, the healthy controls came from two different sources, 7 from University of Louisville and 5 from John Hopkins University. Publicly available proteomic data from PBMCs was not available for the conditions in our study, and therefore, only the liver tissue datasets were validated using independent data. Information regarding the RNAseq liver tissue validation dataset can be found in our previous publication [11].

### RNAseq Classification and Feature Selection Pipeline

The detailed methods used to classify RNAseq counts and identify best genes are described in [11]. Briefly, the classification was performed using nested cross-validation with feature selection. Features were selected using either differential expression software or information gain algorithm. Additionally, outlier features were removed prior to feature selection. Domain expertise was incorporated into the pipeline via enrichment analysis. For each dataset, multiple pipeline configurations were executed, resulting in multiple, promising, candidate gene sets. For each dataset, we then selected a single best gene set that maximized classification performance and in-silico biological relevancy (attained via enrichment analysis), while minimizing the gene set size. The methods used throughout were focused on minimizing the possibility of overfitting. Note that for any given pipeline configuration, there is a resultant set of genes (candidate gene set). Subsequently, when referring to candidate or best gene sets, we are also referring to the pipeline configurations that resulted in those gene sets.

### Proteomic Classification and Feature Selection Pipeline

Methods used to classify proteomic counts and identify best proteins were similar to the methods used for analysis of RNAseq data with the following exceptions.

#### Feature sizes

The feature sizes for proteomic data were largely based on our findings when dealing with RNAseq data. Due to smaller number of proteomic samples the maximum number of features used was reduced from 500 to 200. The following feature sizes were selected: 15, 25, 35, 50, 60, 70, 80, 90, 100, 150, and 200.

#### Imputation

Unlike the RNAseq data, the proteomic data contained missing values. We used median and replacement with zero imputation strategies to address this. Median imputation replaces missing values using the median along each column (feature, in this case protein). Zero imputation replaces all missing values with zeros.

Imputed values were used for proteins that were missing data for a small number of samples. The following imputation thresholds were used 0%, 5%, and 10%. That is, values for a given protein were only imputed if less than the threshold % of total samples were missing data. Threshold of 0% means no imputation took place and all proteins with missing values were removed.

#### Differential expression feature selection

Cuffdiff [14] was used for the differential expression analysis of the RNAseq data, while we used INFERNORDN to perform differential expression analysis with proteomic counts [15]. Proteins were filtered by q-value ≤ 0.05. Afterward, any proteins that had too much missing data (above imputation threshold) were removed.

#### In silico biological validation and best protein set selection

Enrichr [16], which was used for RNAseq data analysis, was replaced with AGOTOOL [17] for enrichment analysis of proteins. When selecting the best protein set, an identical algorithm was used for both transcriptomic and proteomic data, with one exception. That is, for proteomic data, protein sets produced by configurations with the least imputation were preferred for selection.

### Analysis outline

**Fig 1:**
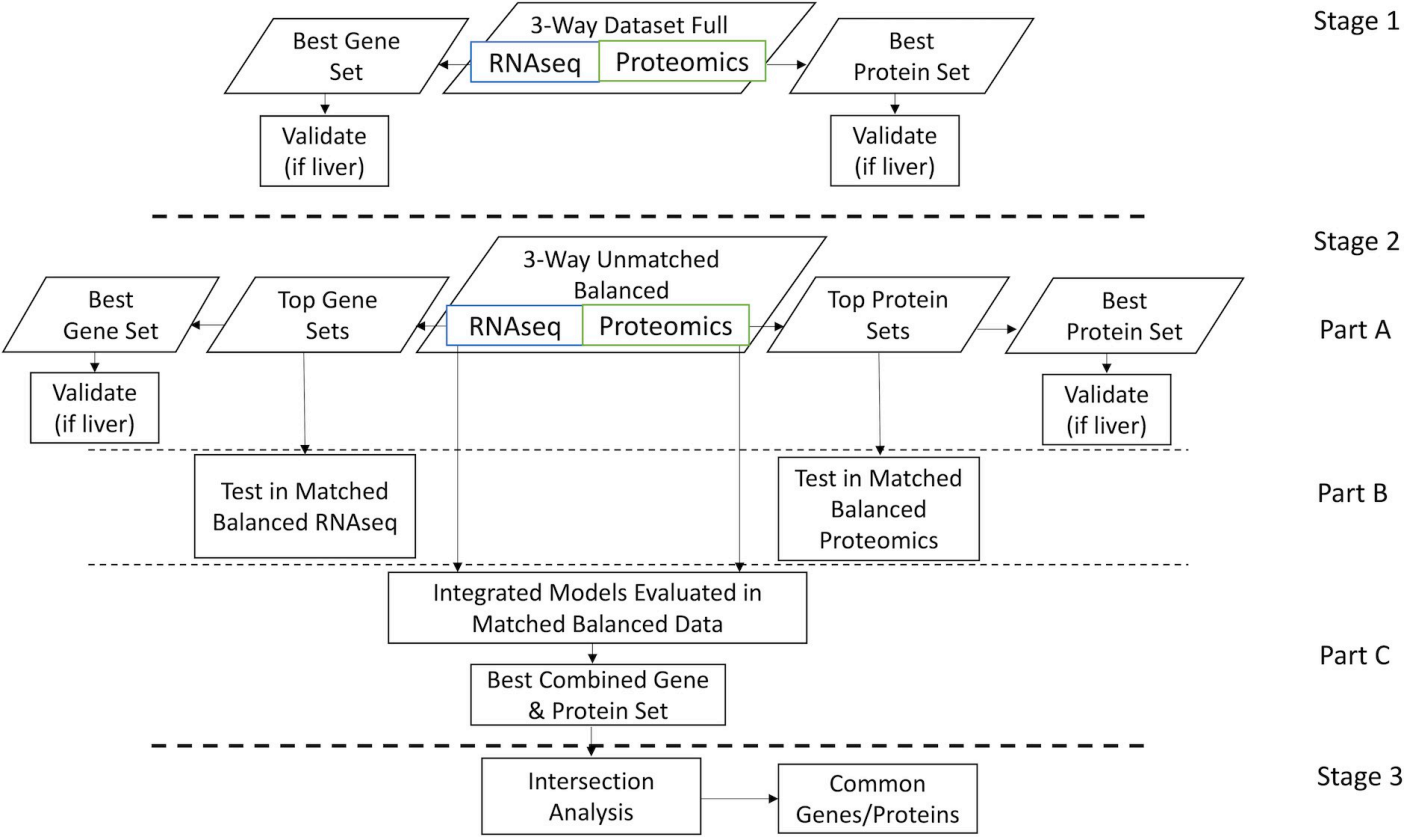
Flowchart of the 3 stages of the analysis. **Stage 1: Separate analyses of full RNAseq and proteomics datasets (Liver 3-Way RNAseq Full, Liver 3-Way Proteomics Full, PBMC 3-Way RNAseq Full, and PBMC 3-Way Proteomics Full).** To simplify the flowchart, we are only showing one representative dataset, which we will refer to as “3-Way Full Datasets”. Stage 2: Training ML models in unmatched balanced data with subsequent testing and integration in matched balanced data. Part A: Identification of top transcriptomic and proteomic pipeline configurations along with their corresponding gene and protein sets for unmatched balanced datasets. Part B: Evaluation of top performing models with their corresponding gene and protein sets from part A in matched balanced data. Part C: Integration of paired sets of the top performing gene and proteomics models with their corresponding gene and protein sets, in matched balanced data. Stage 3: Intersection analysis of the combined best gene-protein sets for liver samples and for PBMCs.

The analysis pipeline was divided into the 3 stages, which are shown in Fig 1.

#### Stage 1 (No Integration)

**Fig 2:**
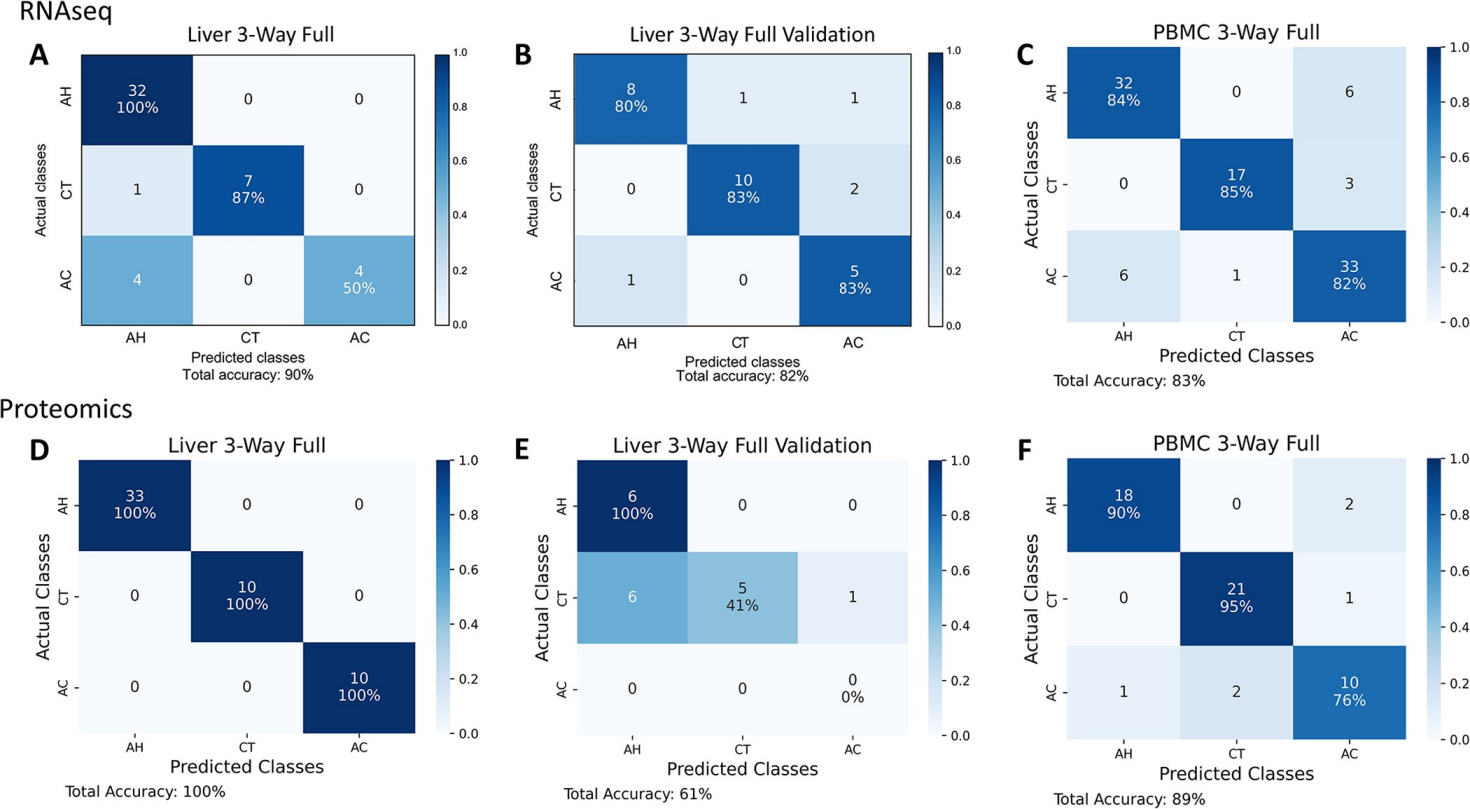
Confusion matrices corresponding to the best gene and protein sets of the full datasets and the liver tissue validation datasets. The Liver 3-way Full best gene and protein sets contained 33 genes and 27 proteins, respectively. The PBMC 3-Way Full best gene and protein sets contained 16 genes and 28 proteins, respectively. (A) Confusion matrix for classification of Liver 3-Way Full RNAseq dataset using best gene set identified by filter feature selection. The diagonal contains the number and percentage of the correctly predicted samples. (B) Confusion matrix for classification of AH, AC, and healthy control (CT) samples within independent validation RNAseq dataset. (C) Confusion matrix for classification of PBMC 3-Way Full RNAseq dataset using best gene set identified by filter feature selection. (D) Confusion matrix for classification of Liver 3-Way Full proteomic dataset using best protein set identified by filter feature selection. (E) Confusion matrix for classification of AH, AC, and CT samples within independent validation proteomic dataset. (F) Confusion matrix for classification of PBMC 3-Way Full proteomic dataset using best protein set identified by filter feature selection.

In the first stage, we used machine learning approaches with nested cross-validation to separately classify the Full datasets (Liver 3-Way RNAseq Full, Liver 3-Way Proteomics Full, PBMC 3-Way RNAseq Full, and PBMC 3-Way Proteomics Full). This enabled us to identify the best genes and proteins, independently of each other, for both sample types using our RNAseq and proteomic pipelines. Refer to Fig 2 for the classification performance for Stage 1.

#### Stage 2 (Integration)

Part A:

We performed the same type of analyses as in Stage 1, i.e. nested cross-validation, to classify the Liver 3-Way Unmatched Balanced and PBMC 3-Way Unmatched Balanced gene and protein datasets. Each pipeline configuration produced a unique candidate gene/protein set. We noted several best performing candidate gene and protein sets for later use in parts B and C.

**Fig 3:**
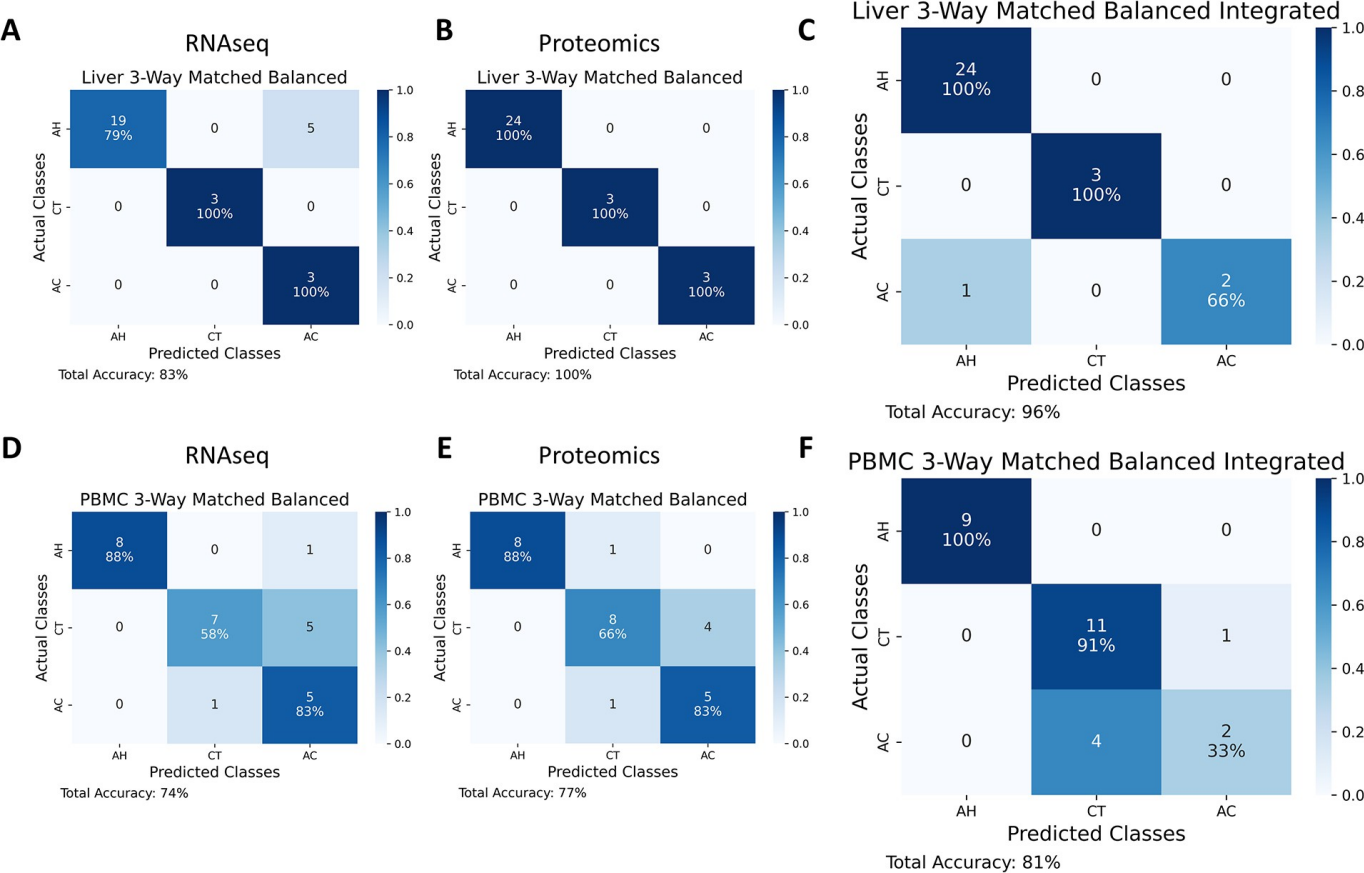
Confusion matrices corresponding to the best gene and protein sets in the matched balanced data set tested separately, and tested with the integrated gene/protein set. Confusion matrices corresponding to the best gene and protein sets (59 genes and 19 proteins, respectively) evaluated within Liver 3-Way Matched Balanced data and within PBMC 3-Way Matched Balanced data (16 genes and 33 proteins, respectively). (A) Confusion matrix for classification of Liver 3-Way Matched Balanced RNAseq dataset using best gene set identified by filter feature selection. (B) Confusion matrix for classification of Liver 3-Way Matched Balanced proteomic dataset using best protein set identified by filter feature selection. (C) Confusion matrix for classification of Liver 3-Way Matched Balanced dataset using a combination of best gene and protein sets. (D) Confusion matrix for classification of PBMC 3-Way Matched Balanced RNAseq dataset using best gene set identified by filter feature selection. (E) Confusion matrix for classification of PBMC 3-Way Matched Balanced proteomic dataset using best protein set identified by filter feature selection. (F) Confusion matrix for classification of PBMC 3-Way Matched Balanced dataset using a combination of best gene and protein sets.

Part B:

We trained classifiers, corresponding to the best performing RNAseq and proteomic ML pipeline configurations from part A, on the entirety of unmatched balanced data. The resulting ML models were then tested in matched balanced data. This would serve as a reference, to which we could later compare the integrated model, as shown in Fig 3.

Part C:

Pairings of the best performing RNAseq and proteomic ML models for each sample type from part B (using their corresponding gene/protein sets) were integrated and evaluated in matched balanced data using cross-validation (Table AA in S1 Text for models tested for the liver samples, and Table AD in S1 Text for the PBMC models tested). The integration was performed by supplying the output prediction probabilities from each pair of RNAseq and proteomic models as input into an integrated model. The pair of candidate gene and candidate protein sets that attained the best classification accuracy was reported as the best combined gene and protein panel. The performance of integrated model in matched balanced data was compared to the performance of separate (RNAseq and proteomic) models in matched balanced data (from part B) as shown in Fig 3.

#### Stage 3 (Intersection)

In the third stage, we examined which genes and proteins matched within the best gene and protein panel. That is, we can consider a protein and a gene that codes for it, as a match.

### Validation in independent liver tissue data

All liver tissue ML models (RNAseq and proteomic) were validated in independent liver tissue validation data. Briefly, the ML model that performed best during nested cross-validation was trained on entirety of our liver tissue data. This trained classifier was then evaluated in independent liver tissue validation data. The methods for independent validation were identical for both RNAseq and proteomic datatypes. The further description of these methods can be found in our previous publication [11] methods.

### Machine learning classifiers

The classifiers used in the individual analysis of the transcriptomic and proteomic data were: k nearest neighbors (kNN), logistic regression (LR), and support vector machine (SVM). For the integrated transcriptomic and proteomic analysis, we used only logistic regression and linear kernel SVM classifiers, due to ease of interpretation. Within the integrated model, the models that directly utilized the RNAseq and proteomic counts were either LR or linear kernel SVM. The classifier that used the prediction probabilities supplied via the RNAseq and proteomic models was LR with default hyperparameters. The LR model has been shown to be well suited for small sample size proteomic data previously [18]. Both LR and SVM classifiers were regularized.

### Feature importance

The combined gene-protein panels for integrated Liver 3-Way and integrated PBMC 3-Way datasets were evaluated for feature importance. Feature importance was evaluated separately for genes and proteins due to the nature of machine learning architecture. The feature importance was evaluated using trained model coefficients. Visualizations of feature importance for integrated Liver 3-Way and integrated PBMC 3-Way datasets can be found in S1 Text.

### Summary of computational methods

Table 4 contains a summary of the computational methods used in the final configurations of the ML models for the RNAseq and Proteomics datasets. Further details can be found in S1 Text.

**Table 4:** Summary of methods used with transcriptomic and proteomic data types.

Data Type	Feature Selection	Feature Sizes	Imputation	ML Classifiers	In-silico Biological Validation
Transcriptomic	Filter (DE, IG)	10, 25, 50, 100, 150, 200, 250, 300, 350, 400, 450, 500	None	LR, kNN, SVM	Enrichr
Proteomic	Filter (DE)	15, 25, 35, 50, 60, 70, 80, 90, 100, 150, 200	Median and Zero (Thresholds: 0%, 5%, and 10%)	LR, kNN, SVM	AGOTOOL

## Results

### Classification of Liver 3-Way Full (AH vs Healthy vs AC)

The gene and protein sets produced via various methods were compared according to classification performance and biological validation scores in order to select the best gene and protein sets. The best gene set contained 33 genes, attained 90% accuracy in main data and 82% accuracy in validation data (Fig 2A and 2B). The best protein set contained 27 proteins, and attained 100% accuracy in main data and 61% accuracy in validation data (Fig 2D and 2E). RNAseq and proteomic data proved similarly effective at classifying our Liver 3-Way samples. However, the best gene set derived from RNAseq data achieved better performance in RNAseq validation data than the best protein set derived from proteomic data achieved in proteomic validation data. The heatmaps of RNAseq and proteomic counts can be found in Figures A-H in S1 Text. The enriched pathways, tissues, and diseases for best gene and protein sets can be found in the Tables E and H in S1 Text. The best gene and protein sets for each dataset are shown in Table 5.

**Table 5:** Best genes and proteins for each dataset. For the integrated datasets, the matching genes and proteins are bolded.

Dataset	Genes	Proteins
Liver 3-Way Full	*AKR1B10*, *C15orf52*, *CFTR*, *CREB3L3*, *CXCL6*, *CYP2A7*, *CYP2B6*, *DBNDD1*, *EEF1A2*, *EPS8L1*, *FAM198A*, *FCGR3B*, *FCN3*, *FITM1*, *GPC3*, *GPNMB*, *HAMP*, *HAO2*, *IGSF9*, *KRT23*, *LCN2*, *LYZ*, *MMP7*, *MT1G*, *PLA2G2A*, *PPP1R1A*, *RGS1*, *S100A8*, *SCTR*, *STAG3*, *TMEM132A*, *TREM2*, *VCAN*.	*ACBP*, *ADH1A*, *ADH1B*, *ADH4*, *ADH6*, *ALBU*, *ARF3*, *CD34*, *CO1A2*, *CP1A2*, *CP3A4*, *CP3A7*, *CRP*, *DDTL*, *ERI3*, *FABPL*, *GSTA1*, *GSTA2*, *GSTM4*, *H2B1C*, *K2C79*, *K2C80*, *LDH6A*, *MFAP4*, *PAL4C*, *SAA1*, *UDB17*.
PBMC 3-Way Full	*ETS2*, *FLVCR2*, *FPR1*, *GRB10*, *IMPA2*, *ITGAM*, *ITGB2*, *LILRA5*, *MYO7A*, *PTGR1*, *RAB31*, *RNASE2*, *SERPINB1*, *SLC36A1*, *ST14*, *TLR4*.	*APOA1*, *BLVRB*, *CATS*, *CSRP1*, *EST1*, *FIBA*, *FIBB*, *FIBG*, *G6B*, *GP1BB*, *GPIX*, *HBD*, *ILK*, *ITA2B*, *ITB3*, *LTBP1*, *MYL9*, *PMGE*, *RAP1A*, *RSU1*, *SDPR*, *SEP11*, *SRC*, *TBA4A*, *TOR4A*, *TSP1*, *URP2*, *VINC*.
Liver 3-Way Matched Balanced Integrated	*ACKR1*, *AKR1B10*, *BBOX1*, *C15orf52*, *CFTR*, ***CLEC4M***, *CREB3L3*, *CSF3R*, *CXCL1*, *CXCL6*, *DCDC2*, *DHODH*, *DHRS2*, *F3*, *FABP4*, *FAM118A*, *FCGR3B*, *FCN3*, *GADD45B*, *GADD45G*, *GPC3*, ***GSTA2***, *HAMP*, *HAO2*, *ID4*, *IGSF9*, *IL7R*, *KRT23*, *LBP*, *LCN2*, *LRG1*, *MARCO*, *MMP7*, *MT1A*, *MT1G*, *MT1H*, *MT1M*, *MT1X*, *MUC13*, *MUC6*, *NRTN*, *PAPLN*, *PID1*, *PLA2G2A*, *PLCB1*, *PPP1R1A*, *S100A12*, *S100A8*, *S100A9*, *SLC13A5*, *SLC22A1*, *SOCS1*, *SPINK1*, *STAG3*, *STMN2*, *TREM2*, *TRIB3*, *VSIG2*, *VTCN1*.	*ACBP*, *ADH1A*, *ADH1B*, *ADH4*, *ADH6*, *ALBU*, *ASSY*, *CD34*, ***CLC4M***, *CO1A2*, *CP1A2 CRP*, *CYB5*, *ERI3*, ***GSTA1***, *HBAZ*, *LDH6A*, *SAA1*, *UDB17*.
PBMC 3-Way Matched Balanced Integrated	*AHSP*, *ALAS2*, *CA1*, *CD177*, *CDK10*, *EHMT1*, *HBD*, *HBM*, *IFI27*, *IL1R2*, *MECP2*, *MMP8*, *MMP9*, ***SELENBP1***, *SLC4A1*, *TANGO2*.	*ACTN1*, *ALBU*, *CCL5*, *CXCL7*, *FHL1*, *FIBA*, *FIBB*, *FIBG*, *FRIL*, *FSTL1*, *GP1BB*, *ILK*, *ITA2B*, *ITB1*, *ITB3*, *LIMS1*, *LYSC*, *MYL9*, *PP14A*, *RAP1A*, *RS4Y1*, ***SBP1***, *SDPR*, *TBA4A*, *TBA8*, *TBB1*, *TPM2*, *TRML1*, *TSN15*, *TSP1*, *URP2*, *VINC*, *VTDB*.

### Classification of PBMC 3-Way Full (AH vs Healthy vs AC)

The best gene set contained 16 genes and attained 83% accuracy in main data (Fig 2C). The best protein set contained 28 proteins and attained 89% accuracy in main data (Fig 2F). RNAseq and proteomic data proved equally effective at classifying our PBMC 3-Way samples. The heatmaps of RNAseq and proteomic counts can be found in Figures I-L in S1 Text. The enriched pathways, tissues, and diseases for best gene and protein sets can be found in the Tables K and N in S1 Text. The best gene and protein sets for each dataset are shown in Table 5.

### Classification of Liver 3-Way Matched Balanced (AH vs Healthy vs AC)

#### Integration of genes and proteins

The best gene set and protein set derived from Liver 3-Way Unmatched Balanced datasets were evaluated in Liver 3-Way Matched Balanced datasets separately and in combination. Using the best gene set of 59 genes we attained 83% classification accuracy within matched balanced RNAseq data (Fig 3A). Using the best protein set of 19 proteins we attained 100% classification accuracy within matched balanced proteomic data (Fig 3B). Using a combination of best gene and protein sets, we attained 96% accuracy in matched balanced integrated data (Fig 3C). Additionally, we generated a one-vs-rest micro-averaged receiver operating characteristic (ROC) curve for the integrated Liver 3-Way model, which resulted in AUC of 1.0 (Figure AE in S1 Text). The constituent transcriptomic (59 genes) and proteomic (19 proteins) models resulted in AUCs of 0.94 and 1.0 respectively (Figures AF and AG in S1 Text).

#### Intersection

Additionally, we examined which biomarkers were shared between the best gene and protein sets of the integrated model with liver tissue. The *CLEC4M-CLC4M*, *GSTA1-GSTA2* were found in common. The *CLEC4M-CLC4M* was a direct match, while the *GSTA1* (protein) was a familial match with *GSTA2* (gene). If the genes and proteins had been selected randomly from among significantly differentially expressed genes and proteins, an expected 0.12 would be shared. Calculation of expected value can be found in S1 Text. Therefore, we have identified more biomarkers in common than expected. Best gene and protein sets were commonly enriched for several different inflammation pathways. The best protein set was more strongly enriched for metabolism pathways than the best gene set (Tables Q and T in S1 Text).

### Classification of PBMC 3-Way Matched Balanced (AH vs Healthy vs AC)

#### Integration of genes and proteins

The best gene and protein sets derived from PBMC 3-Way Unmatched Balanced datasets were evaluated in PBMC 3-Way Matched Balanced datasets separately and in combination. Using the best gene set of 16 genes we attained 74% classification accuracy within matched balanced RNAseq data (Fig 3D). Using the best protein set of 33 proteins we attained 77% classification accuracy within matched balanced proteomic data (Fig 3E). Using a combination of best gene and protein sets, we attained 81% accuracy in matched balanced integrated data (Fig 3F). We also generated a one-vs-rest micro-averaged ROC curve for the integrated PBMC 3-Way model, which resulted in AUC of 0.96 (Figure AK in S1 Text). The constituent transcriptomic (16 genes) and proteomic (33 proteins) models resulted in identical AUCs of 0.89 (Figures AL and AM in S1 Text).

#### Intersection

With the integrated model for PBMCs, the *SELENBP1-SBP1* gene-protein was found in common between the best gene and protein sets. For a random selection from the significantly differentially expressed genes and proteins, we calculated that an expected 0.05 would be shared. Thus, more biomarkers were found to be shared than expected. The best gene and protein sets for PBMCs were mainly enriched for several different inflammation and cancer related pathways (Tables W and Z in S1 Text).

## Discussion

In this study, we used machine learning approaches with transcriptomics and proteomics data from liver tissue and PBMCs to effectively classify samples from participants with alcohol-associated hepatitis (AH), alcohol-associated cirrhosis (AC), and healthy controls. Liver tissue models outperformed PBMC models by a small margin in our data. Both transcriptomic and proteomic liver tissue ML models generalized relatively well in the independent validation data. Overall, the transcriptomic and proteomic models performed similarly well in each sample type.

The integration of proteomic and transcriptomic data did not increase classification accuracy with liver tissue, mainly because the classification accuracy was already high in both data types separately. For PBMCs, on the other hand, the integration improved classification accuracy slightly. While the performance of PBMC biomarkers is less than that of liver tissue biomarkers for classification of ALDs, the integration of multiple -omics data types could help close the gap in the future. To our knowledge, this is the first study in which a combined PBMC gene-protein expression biomarker panel has been identified for distinguishing AH, AC, and healthy controls.

Of special interest are the gene-protein matches present in the combined gene-protein sets identified for Liver 3-Way and PBMC 3-Way Matched Balanced Integrated datasets. All the matched liver tissue genes have been established as relevant biomarkers of liver disease in prior literature. *CLEC4M* has been identified as prognostic liver tissue biomarker of hepatocellular carcinoma [19]. *GSTA1* and *GSTA2* have been previously identified as biomarkers of liver injury (including ethanol injury) and hepatocellular carcinoma respectively [20,21]. Less is known about the role of the matched PBMC genes in liver disease. Differential expression of *SELENBP1* in PBMCs of hepatocellular carcinoma patients has been established previously [22]. The differential expressions of these biomarkers in both transcriptomic and proteomic data increases our confidence in their significance.

The gene-protein panels for Liver 3-Way and PBMC 3-Way integrated datasets were examined using enrichment analysis. The genes and proteins were examined separately. For Liver 3-Way the proteins were overwhelmingly enriched for metabolic pathways, including ethanol metabolism (Table AB in S1 Text). Notably, many of the key liver proteins are alcohol dehydrogenases, some of which have been implicated in alcohol and liver disorders [23,24]. Other notable proteins include *CRP*, *SAA1*, *ALBU*. All of these have been previously established as diagnostic biomarkers of inflammatory liver diseases [25,26,27]. The genes were enriched for homeostasis, metabolism, and inflammatory pathways (Table AC in S1 Text). For PBMC 3-Way both the genes and proteins were enriched for blood processes, immune system functions, and cellular movement (Tables AE and AF in S1 Text). Some of the PBMC proteins have been previously connected to liver disease including *FSTL1*, *TSP1*, *CCL5*, and *TPM2* [28,29,30,31]. Overall, the identified genes and proteins are consistent with previous findings.

We have discussed the importance of using appropriate ML methods for analysis of small sample size RNAseq data [11] previously. Our recommendations for analysis of small sample size proteomic data are largely similar. In addition to the importance of filter feature selection we would like to highlight the importance of nested cross-validation (NCV) and performing feature selection within both inner and outer loops of NCV. The use of nested cross validation is necessary to separate model selection and evaluation if hyperparameter tuning is being done. Meanwhile, it is necessary to perform feature selection within nested cross validation to avoid data leakage and the resulting bias [32]. The use of in-silico biological relevancy (via enrichment analysis) in our pipeline was also important as it decreased overfitting by favoring feature sets that corresponded to existing literature.

The liver tissue proteomics model’s performance in independent validation data was lower than expected. The healthy control samples in independent validation proteomic dataset were collected from two different clinical sources. Most misclassified healthy controls were from one of the two sources. The heterogeneity in healthy samples may explain their unexpectedly poor classification performance. The PBMC models could not be independently validated due to lack of relevant public data. However, the methods used to derive the best biomarkers were identical in both tissues. The integrated models also could not be validated due to lack of appropriate publicly available genomic data in which both RNAseq and proteomics were available for the same individuals. A larger sample size and an independent integrated validation cohort are needed to further investigate these biomarkers.

Integrating two -omics datatypes further amplified the challenges we encountered in our earlier work [11]. The number of genes and proteins for each sample is much larger than the number of samples in our dataset. This makes data prone to overfitting, since a complex model can perfectly separate a small number of samples. Some of the other challenges were ensuring that the integrated model did not have a bias toward transcriptomic or proteomic features, performing feature selection with integrated gene and protein expression data, and addressing partial matching between our transcriptomic and proteomic samples (most were obtained from the same individuals, but some were not).

Overall, the integration of proteomic and transcriptomic data from liver tissue and PBMCs for ALD proved promising in two aspects. In the case of PBMCs in our study, combining transcriptomic and proteomic biomarkers was more effective than using either type of biomarkers alone for classification. Additionally, by examining both transcriptomic and proteomic data, we were able to identify gene-protein pairs that were significantly differentially expressed in both domains and were thus more likely to be relevant to the liver disease conditions in question. The possibility of using PBMCs to distinguish among alcohol-associated liver diseases is encouraging, and the relevant biomarkers warrant further examination.

## Supporting information

S1 Text Supplemental methods and supplemental results for this study. (PDF)
