# Supplementary material for: Identification of integrated proteomics and transcriptomics signature of alcohol-associated liver disease using machine learning
Source: PLOS Digit Health. 2024 Feb 9;3(2):e0000447. doi: 10.1371/journal.pdig.0000447 (PMC10857706; doi:10.1371/journal.pdig.0000447)
Supplement: S1 Text — (PDF) [file pdig.0000447.s001.pdf]

SUPPORTING INFORMATION

Identification of integrated proteomics and transcriptomics signature of alcohol-associated liver disease using machine learning

Authors: Stanislav Listopad[1,#a*], Christophe Magnan[1], Le Z. Day[2], Aliya Asghar[3], Andrew Stolz[4], John A. Tayek[5], Zhang-Xu Liu[4], Jon M. Jacobs[2], Timothy R. Morgan[3], Trina M. Norden-Krichmar[1,6]*

Author Affiliations:

[1]Department of Computer Science, University of California, Irvine, California, United States of America

[2]Biological Sciences Division and Environmental and Molecular Sciences Division, Pacific Northwest National Laboratory, Richland, Washington, United States of America

[3]Medical and Research Services, VA Long Beach Healthcare System, Long Beach, California, United States of America

[4]Division of Gastrointestinal & Liver Diseases, Department of Medicine, Keck School of Medicine, University of Southern California, Los Angeles, California, United States of America

[5]Lundquist Institute for Biomedical Innovation at Harbor-UCLA Medical Center, Department of Internal Medicine, David Geffen School of Medicine, University of California Los Angeles, Torrance, California, United States of America

[6]Department of Epidemiology and Biostatistics, University of California, Irvine, California, United States of America

[#a]Current Address: Department of Neuroscience, Scripps Research, La Jolla, California, United States of America

Table of Contents:

Contents

1. **SUPPLEMENTARY METHODS** 5
a. Inclusion and Exclusion Criteria (RNAseq and Proteomics): 5
b. Sample Collection and Processing (RNAseq and Proteomics): 6
c. RNAseq alignment (RNAseq): 6
d. Feature Transformation (RNAseq and Proteomics): 6
e. Nested Cross-Validation Setup (RNAseq and Proteomics): 6
f. Hyper-parameter Tuning (RNAseq and Proteomics): 7
g. Feature Selection Strategies (RNAseq and Proteomics): 8
h. Differential Expression (DE) Feature Selection (RNAseq and Proteomics): 8
i. Information Gain (IG) Feature selection (RNAseq): 9
j. Imputation (Proteomics): 9
k. Feature Sizes (RNAseq and Proteomics): 9
l. Performance Metrics (RNAseq and Proteomics): 10
m. Machine Learning Classifiers (RNAseq and Proteomics): 10
n. Sample Size Calculation (RNAseq and Proteomic): 10
o. Enrichr and AGOTOOL Libraries (RNAseq and Proteomics): 11
p. Regular Expression (Regex) Patterns for Enrichment Analysis (RNAseq and Proteomics): 12
q. Impact of Outlier Gene (Feature) Removal – Variance, Intersection, and Union Filtering (RNAseq and Proteomics): 12
r. Summary of Computational Methods (RNAseq and Proteomics): 13
s. Candidate Gene and Protein Sets (RNAseq and Proteomics): 14
t. Best Gene Set Selection (RNAseq): 15
u. Best Protein Set Selection (Proteomics): 16
v. Codebase (RNAseq and Proteomics): 16
2. SUPPLEMENTAL RESULTS 17
a. Liver 3-Way Full (AH vs Healthy vs AC) 17
i. TRANSCRIPTOMIC SECTION for Liver 3-Way Full dataset: 17
ii. PROTEOMIC SECTION for Liver 3-Way Full dataset 24
c. PBMC 3-Way Full (AH vs Healthy vs AC) 31
i. TRANSCRIPTOMIC SECTION for PBMCs 3-Way Full dataset: 31
ii. PROTEOMIC SECTION for PBMC 3-Way Full dataset: 34

c. Liver 3-Way Unmatched Balanced (AH vs Healthy vs AC)....38
 i. TRANSCRIPTOMIC SECTION for Liver 3-Way Unmatched Balanced dataset: 38
 ii. PROTEOMIC SECTION for Liver 3-Way Unmatched Balanced dataset:....47
d. PBMC 3-Way Unmatched Balanced (AH vs Healthy vs AC) 55
 i. TRANSCRIPTOMIC SECTION for PBMC 3-Way Unmatched Balanced dataset: 55
 ii. PROTEOMIC SECTION for PBMC 3-Way Unmatched Balanced dataset:....61
e. Integrated Analysis of LV 3-Way (AH vs Healthy vs AC)....66
f. Integrated Analysis of PBMC 3-Way (AH vs Healthy vs AC)....75
g. Intersection Analysis of LV 3-Way Matched Balanced Integrated (AH vs Healthy vs AC)....83
h. Intersection Analysis of PBMC 3-Way Matched Balanced Integrated (AH vs Healthy vs AC)....83
SUPPLEMENTARY REFERENCES 84

List of abbreviations:

AC: alcohol-associated cirrhosis

AH: alcohol-associated hepatitis

BMI: body mass index

CT: healthy controls

DE: differential expression

DF: Maddrey's discriminant function

FPKM: fragments per kilobase of exon model per million reads mapped

FS: feature selection

IG: information gain

IRB: institutional review board

kNN: k-nearest neighbors

LR: logistic regression

LTCDS: Liver Tissue Cell Distribution System

LV: liver (tissue name)

MCC: Matthew's Correlation Coefficient

MELD: Model for End-Stage Liver Disease

ML: machine learning

NCV: nested cross-validation

PBMC: peripheral blood mononuclear cells

RNA: ribonucleic acid

RNA-seq: RNA sequencing

SCAHC: Southern California Alcoholic Hepatitis Consortium

SVM: support vector machine

1. SUPPLEMENTARY METHODS

Sections **a-c** below briefly describe the collection and processing of the samples from our internal RNAseq and proteomics data sets.

The study was approved by the Department of Veterans Affairs VA Long Beach Healthcare Systems Institutional Review Board (IRB# 1254), by the Human Subjects Committee, Los Angeles Biomedical Research Institute (Project No. 20607-0), University of Southern California Health Sciences Campus Institutional Review Board (Project # HS-13-00815), and by the University of California, Irvine Institutional Review Board, HS #2016-3064. All participants signed written consents prior to providing biospecimens.

Liver tissue and PBMC RNAseq data is being deposited in dbGaP [1]. For information about the independent RNA-seq liver tissue dataset used for external validation, please refer to GSE142530 [2]. Our liver tissue proteomic dataset and independent validation dataset can be found in MassIVE repository (accession number MSV000089168) [3]. PBMC proteomic data is pending deposition in MassIVE.

a. Inclusion and Exclusion Criteria (RNAseq and Proteomics):

A detailed description of the SCAHC consortium inclusion and exclusion criteria has been described previously [1]. The diagnosis for a subset of the alcohol-associated hepatitis patients were biopsy-confirmed at the clinical sites where biopsy is standard-of-care. The alcohol-associated cirrhosis patients were not biopsy confirmed. The diagnosis of alcohol-associated cirrhosis was based on a history of decompensation (e.g., ascites, variceal bleeding),

evidence of nodular liver on imaging (e.g., CT scan or MRI), or portal hypertension (e.g., collateral vessels in the abdomen on CT or MRI).

b. Sample Collection and Processing (RNAseq and Proteomics):

The methods used to process liver tissue and blood samples for RNA-sequencing are described in [1]. The methods used to process liver tissue samples and perform liquid chromatography-tandem mass spectrometry are described in [3]. The methods used to process PBMC samples, for mass spectrometry, are nearly identical to the methods used to process plasma samples in the following publication [4].

c. RNAseq alignment (RNAseq):

For full RNAseq methods, please refer to our previous publication [1]. Briefly, we used hg38 (GRCh38 assembly) human reference genome, downloaded from the UCSC Genome Browser. ChrM was not included in the assembly. We used Ensembl release 91 (Dec 2017) annotation. We used STAR 2.6.0 [5] aligner with default settings (STARCQ).

d. Feature Transformation (RNAseq and Proteomics):

RNAseq counts were transformed using ln(1+count) formula. The proteomic counts did not exhibit same properties as RNAseq counts and were not log transformed.

e. Nested Cross-Validation Setup (RNAseq and Proteomics):

As described in [1], we utilized nested cross-validation to attain the estimates of classification performance for various feature selection (FS) strategies, classifiers, and feature sizes within our data. The best feature (gene and protein) sets selected for each of the datasets were then validated in the independent validation set. The nested cross-validation was implemented in the standard configuration with $k = 5$ in both the inner and outer loops. The outer

loop was used for model evaluation (i.e., classification performance), while the inner loop was used for model selection (i.e., hyper-parameter tuning). The feature selection was done within both inner and outer loops. That is FS was done for each training set in inner and outer loops. This means that effectively there were 30 training sets (25 in inner loop, 5 in outer loop) as part of a single nested cross-validation execution. Feature selection occurred for each of these training sets.

For RNAseq, one of our classification strategies relied on differential expression as computed by Cuffdiff [7], while the other classification strategy was information gain. The proteomic differential expression was computed using INFERNORDN, otherwise the same nested cross validation procedure was applied to both gene and protein expression data [8].

f. Hyper-parameter Tuning (RNAseq and Proteomics):

The hyper-parameter tuning was performed using grid search. The following sets of hyper-parameters were used for each classifier:

- Logistic Regression
    - C: 0.5, 1.0, 2.0, 3.0, 4.0, 5.0.
    - Class Weight: None, Balanced.
    - Solver: Newton-cg, LBFGS, Liblinear, Saga.
- K Nearest Neighbors
    - N Neighbors: 3, 5, 7, 9.
    - Weights: Uniform, Distance.
    - Metric: Euclidean, Manhattan, Chebyshev, Minkowski.
- Support Vector Machine

- Kernel: Linear, Poly, RBF.
- C: 0.5, 1.0, 2.0, 3.0, 4.0, 5.0.
- Class Weight: None, Balanced.
- Degree: 1, 3, 5.
- Gamma: Scale, Auto.

For the integrated analysis portion of the study, only the linear kernel support vector machine was used, as mentioned in the "Machine Learning Classifiers" subsection of the main text methods.

g. Feature Selection Strategies (RNAseq and Proteomics):

Based on preliminary analysis we have identified filter feature selection to be best suited for small sample size RNAseq data. The two filter feature selection methods we selected are: differential expression and information gain.

For proteomic data we immediately settled on using filter feature selection in the form of differential expression. The RNAseq and proteomic data are similar in sample (~10s) and feature sizes (~10,000s). Therefore, we assumed that filter feature selection would be the best approach in both types of data.

h. Differential Expression (DE) Feature Selection (RNAseq and Proteomics):

RNAseq:

For every RNAseq training set all pairwise comparisons were filtered by normalized FPKM (> 1.0) and q-values (< 0.05). All of the genes belonging to each pairwise comparison were then sorted by absolute log2(fold change) value, and the top gene for each pairwise comparison was taken. If that gene was not already in the top genes list, the gene was added to the list. The process was repeated until the desired number of genes was reached.

Proteomics:

The INFERNORDN was used to generate fold changes and q-values for proteomic counts. The results were filtered by q-value (< 0.05). Additionally, depending on imputation threshold, entries that were missing data for too many samples were filtered out. The pairwise DE selection procedure described above was used for proteins as well.

i. Information Gain (IG) Feature selection (RNAseq):

For every training set, the genes within normalized RNA-seq counts were ranked using the scikit-learn's mutual_info_classif function.

j. Imputation (Proteomics):

We used median and replacement with zero imputation strategies. Median: replace missing values using the median along each column (feature, in this case protein). Zero: replace all missing values with zeros.

We only imputed values for proteins that were missing data for small number of samples. The following imputation thresholds were used 0%, 5%, and 10%. That is values for a given protein were only imputed if < threshold % of total samples were missing data. Threshold of 0% means no imputation took place and all proteins with missing values were removed.

k. Feature Sizes (RNAseq and Proteomics):

RNAseq:

We refer to the number of features selected during filter feature selection as "feature size". The feature sizes used with DE & IG feature selection were: 2, 3, 4, 5, 10, 15, 20, 25, and, 50 for LV 2-Way dataset and 10, 25, 50, 100, 150, 200, 250, 300, 350, 400, 450, and 500 for the other three datasets. The feature sizes denote the number of features selected within each training set. We found during preliminary testing that we required at least 5-10 features per training set to

attain reasonable classification performance and that we generally did not see benefit in using more than 500 features per training set. The maximum feature size was also influenced by our power size calculation (that is number of significantly differentially expressed genes within our datasets).

Proteomics:

The feature sizes for proteomic data were largely based on our findings when dealing with RNAseq data. The following feature sizes were selected: 15, 25, 35, 50, 60, 70, 80, 90, 100, 150, and 200.

l. Performance Metrics (RNAseq and Proteomics):

We reported our results using confusion matrices, per-class accuracy, and total accuracy.

m. Machine Learning Classifiers (RNAseq and Proteomics):

For RNAseq and Proteomic classification we decided to use logistic regression (LR), k nearest neighbors (kNN), and support vector machine (SVM) classifiers based on preliminary analysis.

n. Sample Size Calculation (RNAseq and Proteomic):

We expected there to be <= 450 significantly differentially expressed genes (SDEGs) in our RNAseq data based on preliminary power size calculation. Further information regarding sample size calculation for RNAseq data can be found in [1]. In proteomic data we primarily relied on q-value as output by INFERNORDN to establish significance of differentially expressed proteins.

o. Enrichr and AGOTOOL Libraries (RNAseq and Proteomics):

The genes and proteins selected during feature selection were computationally evaluated using gene and protein enrichment analysis via Enrichr [9] and AGOTOOL [10] respectively with pathway, tissue, and disease libraries listed below. Custom code was written using regular expressions to match: a) immune system pathways; b) cell types that comprise blood and liver tissues; c) diseases included the conditions within this study (AH, AC) along with several other liver and blood disorders.

In order to attain the top three Enrichr and AGOTOOL hit tables (Tables C-F in S1 Text) we performed the following steps. Enrichr/AGOTOOL hits for the best gene/protein sets, after matching using the regular expressions, were sorted by adjusted p-value with a cutoff of 0.05. We removed entries with redundant term names or genes/proteins. We then displayed up to three top entries for each category: pathway, tissue, disease.

Enrichr Libraries used:

Pathways: 'BioPlanet_2019', 'WikiPathways_2019_Human', 'KEGG_2019_Human', 'GO_Biological_Process_2018'.

Tissues: 'ARCHS4_Tissues', 'Human_Gene_Atlas'.

Diseases: 'Disease_Perturbations_from_GEO_up', 'Disease_Perturbations_from_GEO_down'.

AGOTOOL Libraries used:

Pathways: ‘GO biological process’, ‘KEGG’, ‘WikiPathways’.

Tissues: ‘Brenda Tissue Ontology’.

Diseases: ‘Disease Ontology’.

p. Regular Expression (Regex) Patterns for Enrichment Analysis (RNAseq and Proteomics):

The regular expression (regex) patterns used for filtering the results returned by Enrichr and AGOTOOL are identical and specified in [1].

q. Impact of Outlier Gene (Feature) Removal – Variance, Intersection, and Union Filtering (RNAseq and Proteomics):

RNAseq:

During our prior analysis of RNA-seq data [1], we discovered several aspects of RNA sequencing that presented challenges to classification performance. One challenge involved expression of non-coding genes that were presumed to be removed via poly(A)-selection. A second challenge that we observed involved genes with aberrant expression that poorly distinguish between the study conditions. Therefore, we developed three strategies for removing these genes: Variance, Intersection, and Union filtering. Variance filtering was implemented by removing genes in which the RNA-seq counts for at least one sample were further than a standard deviation multiplied by the threshold from the mean in any of the conditions (AH, CT, etc.). Throughout the study, we used three threshold values: 2.5, 3.0, and 3.5. The filtered-out genes were not used in the subsequent feature selection process. The Union filter built upon the Variance filter by removing all genes that were either highly variant (as defined above) *or* non-coding as determined by ENSEMBL database's gene "biotype" column. The Intersection filter was similar to the Union filter, except that only the genes that were both highly variant *and* non-coding were removed. In addition to improving the odds of successful classification, the outlier feature filtering was also found to improve in silico biological validation of identified gene

signatures, since protein coding genes are more extensively annotated than non-coding ones. These three filters also removed all genes whose counts were mostly zeroes across all samples.

Proteomics:

The concept of coding and non-coding did not apply to proteins. For proteomics, we simply used variance filter with standard deviation thresholds of 2.5 and 3.0.

r. Summary of Computational Methods (RNAseq and Proteomics):

RNAseq:

Table A in S1 Text: The RNAseq methods used within the study.

Methods	Feature Selection	Outlier Feature Removal	ML Classifiers
Final Configuration	Filter (DE, IG).	Intersection and Union filtering. (Standard deviation thresholds: 2.5, 3.0, 3.5)	LR, kNN, SVM.

All stages of the analysis included the following method configurations for each of the datasets: 2 feature selection strategies (DE, IG), 2 outlier feature removal strategies (Intersection, Union) each paired with three different thresholds (2.5, 3.0, 3.5), and 3 ML classifiers (LR, kNN, SVM). This resulted in a total of 36 configurations. For each configuration there was also a range of possible feature sizes as described in the feature size section above. The nested cross-validation ML metrics were recorded for each of these configurations, for each feature size.

Proteomics:

For proteomics data the settings were further narrowed down to 1 feature selection strategy, 2 outlier feature removal strategies, and 3 classifiers (Table B in S1 Text).

Table B in S1 Text: The proteomic methods used within the study.

Methods	Feature Selection	Outlier Feature Removal	ML Classifiers
Final Configuration	Filter (DE).	None and Variance filtering (Standard deviation thresholds: 2.5, 3.0)	LR, kNN, SVM.

s. Candidate Gene and Protein Sets (RNAseq and Proteomics):

Besides classification, our pipeline was also designed to identify characteristic gene and protein expression signatures to diagnose liver disease using liver tissue and PBMC genomic data. Within nested cross-validation, feature selection was performed for every training set in both inner and outer loops, resulting in 30 total feature sets (5 in outer, 25 in inner) for each feature size. The feature sets selected in the inner loops are not relevant, since the inner loop was only used for hyper-parameter tuning. Therefore, we developed a method of merging the feature sets produced for each of the outer loop training sets. The strategy used was as follows: if a given feature appeared in N out of the 5 (k = 5 in outer loop) gene sets it was added to the merged feature set. After examining the results, we determined that N = 4 and N = 5 yielded our best results for the RNA-seq data, and N = 3, N=4, and N=5 were best for proteomic data. The candidate gene sets were analyzed using Enrichr to establish their biological relevancies, while the candidate protein sets were analyzed using AGOTool. The classification accuracy attained from the associated instance of the nested cross-validation of each candidate feature set was also examined.

t. Best Gene Set Selection (RNAseq):

From the candidate gene sets attained by running the multiple different method configurations, we used the following strategy to select a single best candidate gene set for each dataset. We examined the combination of candidate gene set's size, classification performance, and biological relevancy metrics. The algorithm for picking best gene sets is described below.

1) The candidate gene set size was restricted between 5 (genes per pairwise comparison) to 100 total genes, if possible. Gene set sizes of between 100 and 200 were also considered, if suitable performance was not attained with smaller gene sets. The LV 3-Way dataset contains 3 pairwise comparisons. Therefore, the candidate gene set sizes, using the guidelines above, are as follows: 15-100 genes for LV 3-Way. These guidelines were developed to minimize the chance of under- or overfitting.
2) Biological relevancy as indicated by Enrichr was prioritized over the classification accuracy. That is, gene sets with highest number of pathway, tissue, and disease hits were examined in detail first. Gene sets were only considered if they included at least 10 pathway, 1 tissue, and 3 disease hits. The tissue, pathway, and disease hits were examined to verify that they were relevant to the disease groups.
3) Total and per-class classification accuracies were considered after the in silico biological relevancy. In general, only gene sets within 10% of the best recorded performance (for a given dataset) were considered.

Once a single gene set that best satisfied all 3 criteria was selected, it was used to generate the heatmaps, confusion matrices, and pathway analysis. The liver tissue gene sets selected from our data set were evaluated with the independent validation dataset.

u. Best Protein Set Selection (Proteomics):

The process for selecting the best protein set was identical to best gene set selection, except one additional criterion was added to the best gene set algorithm. In particular, the protein sets generated by configurations with least imputation were preferred. Additionally, the enrichment analysis was performed with AGOTool, rather than Enrichr.

v. Codebase (RNAseq and Proteomics):

Github: https://github.com/staslist/Liver-Disease-Diagnostic-Proteomic-And-Transcriptomic

The repository contains the code used to perform the analysis. Directories and sample names have been removed from the codebase.

2. SUPPLEMENTAL RESULTS

To keep the Supplemental Results section concise, only the tables containing the best performing feature sets are listed herein for each dataset.

a. Liver 3-Way Full (AH vs Healthy vs AC)

i. TRANSCRIPTOMIC SECTION for Liver 3-Way Full dataset:

Classification Accuracies:

kNN / Union:

Table C in S1 Text: The classification performances of k-nearest neighbors model across a range of feature sizes (10-500) and feature selection techniques (differential expression with standard deviation thresholds of 2.5, 3.0, 3.5). The accuracy corresponding to best gene set is highlighted in green.

Feature Size	DE 2.5	DE 3	DE 3.5
10	0.82	0.86	0.83
25	0.85	0.86	0.83
50	0.86	0.85	0.9
100	0.88	0.9	0.85
150	0.88	0.9	0.9
200	0.9	0.9	0.9
250	0.88	0.9	0.91
300	0.88	0.88	0.9
350	0.88	0.88	0.9
400	0.9	0.88	0.88
450	0.9	0.9	0.88
500	0.9	0.9	0.9

Enrichr In Silico Biological Validation:

Union / 5 out of 5 Merge:

Table D in S1 Text: The Enrichr hits Union / 5 out of 5 Merge configuration in LV 3-Way dataset.

Feature Size	DE 2.5	DE 3	DE 3.5
10 - 0/1/0	NA	23/0/8	NA
25 - 2/4/4	4/0/6	34/1/5	30/1/4
50 - 5/8/10	16/0/0	34/4/10	13/4/11
100 - 12/19/21	2/4/0	11/4/13	10/4/14
150 - 19/32/33	0/4/7	9/5/15	**13/5/17**
200 - 27/44/46	1/4/6	6/5/19	2/5/21
250 - 37/55/58	1/4/9	4/5/28	2/5/19
300 - 43/65/71	0/5/9	4/6/30	5/6/26
350 - 54/74/84	1/5/11	4/6/28	5/6/30
400 - 76/89/94	4/5/17	2/6/31	7/6/29
450 - 93/95/109	5/6/24	2/6/31	5/6/32
500 - 111/115/117	12/6/22	11/7/35	11/6/33

Best gene set for Liver 3-Way Full dataset: AKR1B10, C15orf52, CFTR, CREB3L3, CXCL6, CYP2A7, CYP2B6, DBNDD1, EEF1A2, EPS8L1, FAM198A, FCGR3B, FCN3, FITM1, GPC3, GPNMB, HAMP, HAO2, IGSF9, KRT23, LCN2, LYZ, MMP7, MT1G, PLA2G2A, PPP1R1A, RGS1, S100A8, SCTR, STAG3, TMEM132A, TREM2, VCAN – 33 genes.

Heatmaps for Liver 3-Way Full RNAseq dataset:

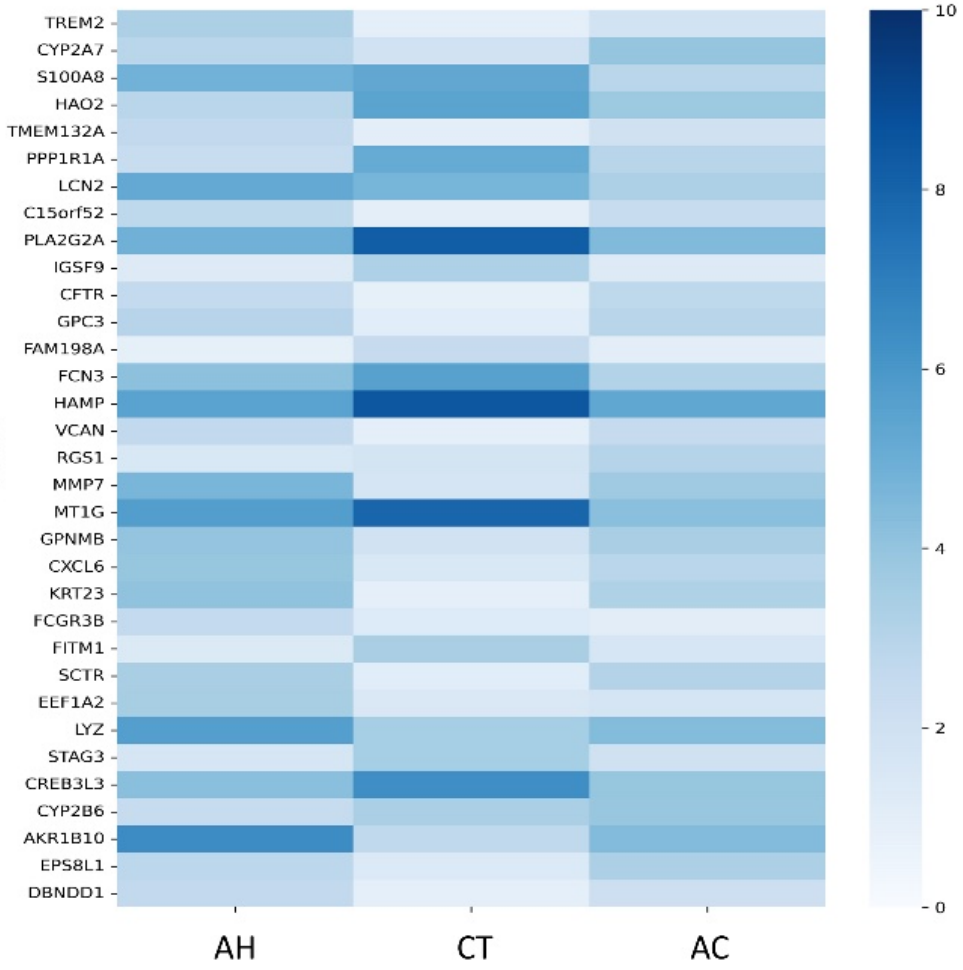

Figure A in S1 Text: Heatmap of RNAseq counts for Liver 3-Way Full dataset averaged per condition.

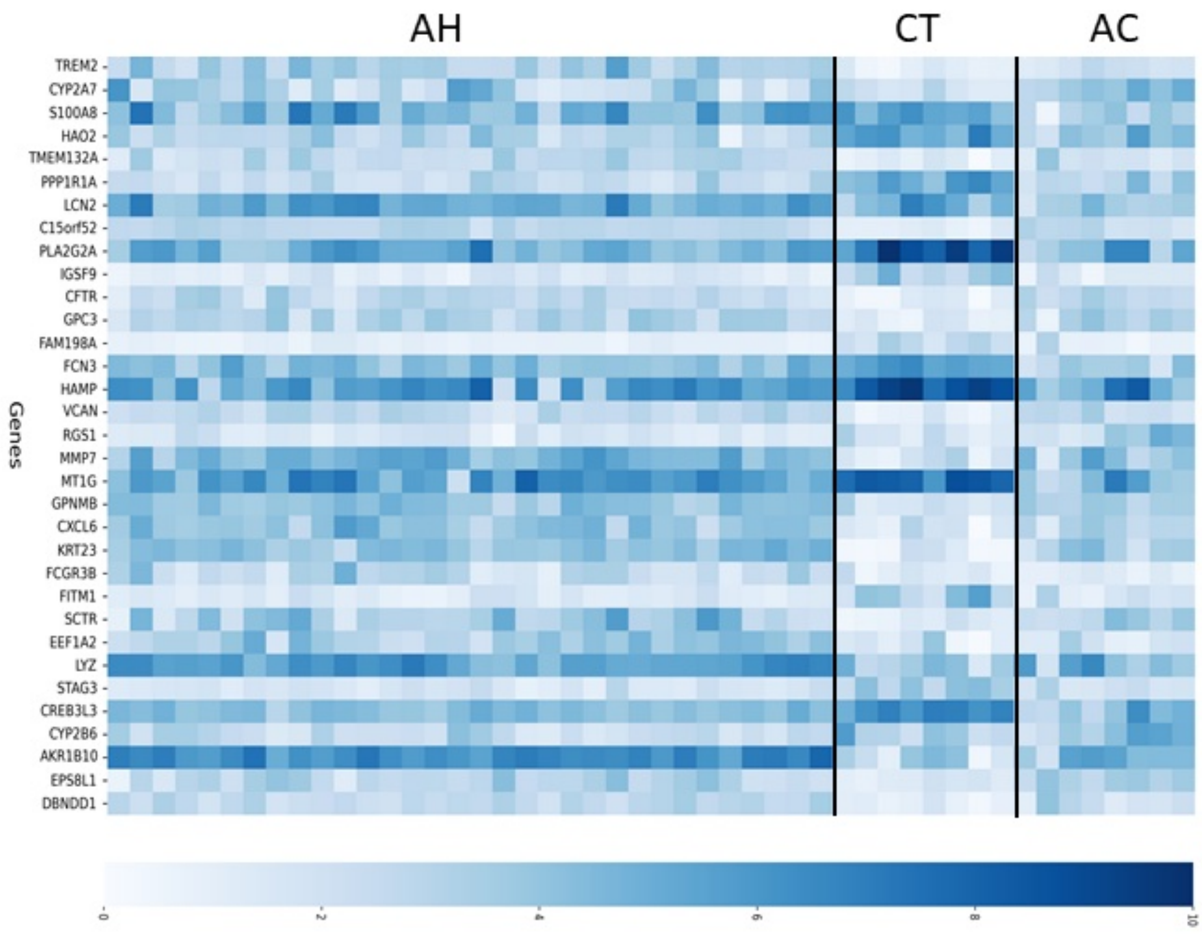

Figure B in S1 Text: Heatmap of RNAseq counts for Liver 3-Way dataset.

Heatmaps for Liver 3-Way Full RNAseq external validation dataset:

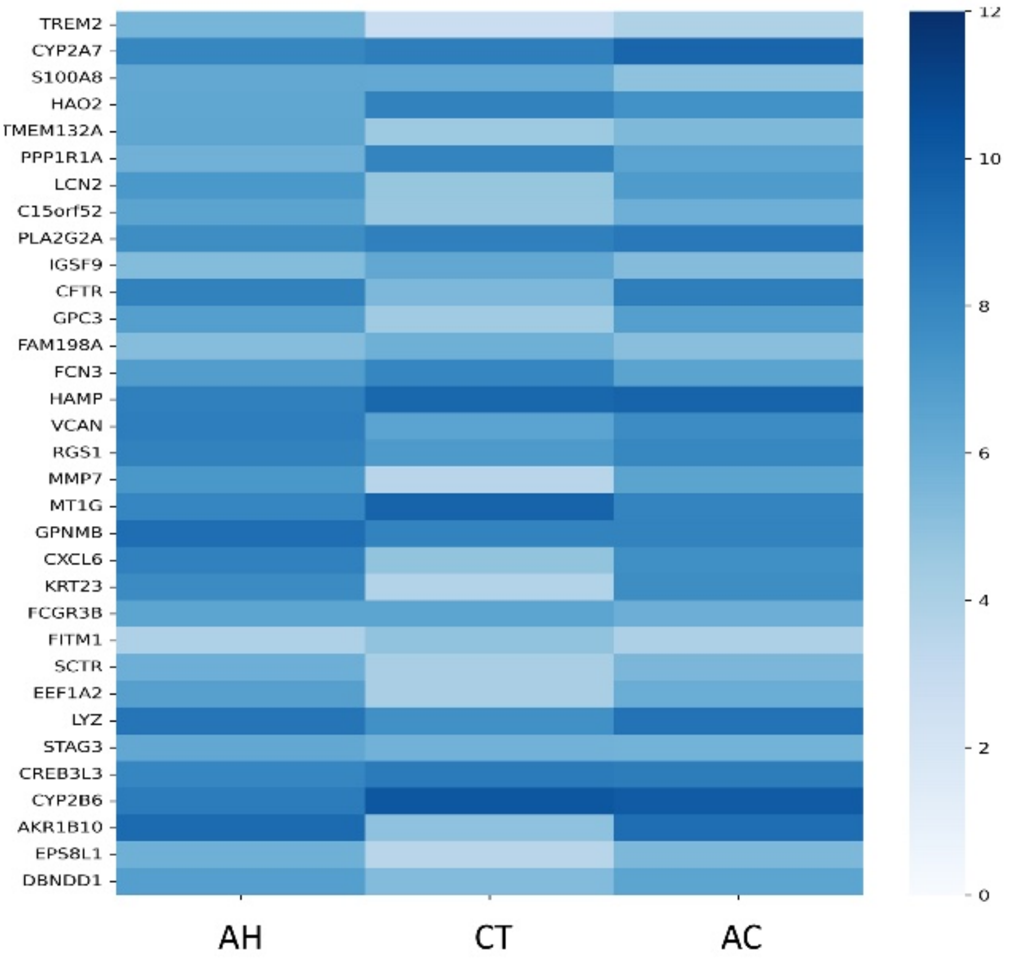

Figure C in S1 Text: Heatmap of RNAseq counts for independent liver validation dataset averaged per condition.

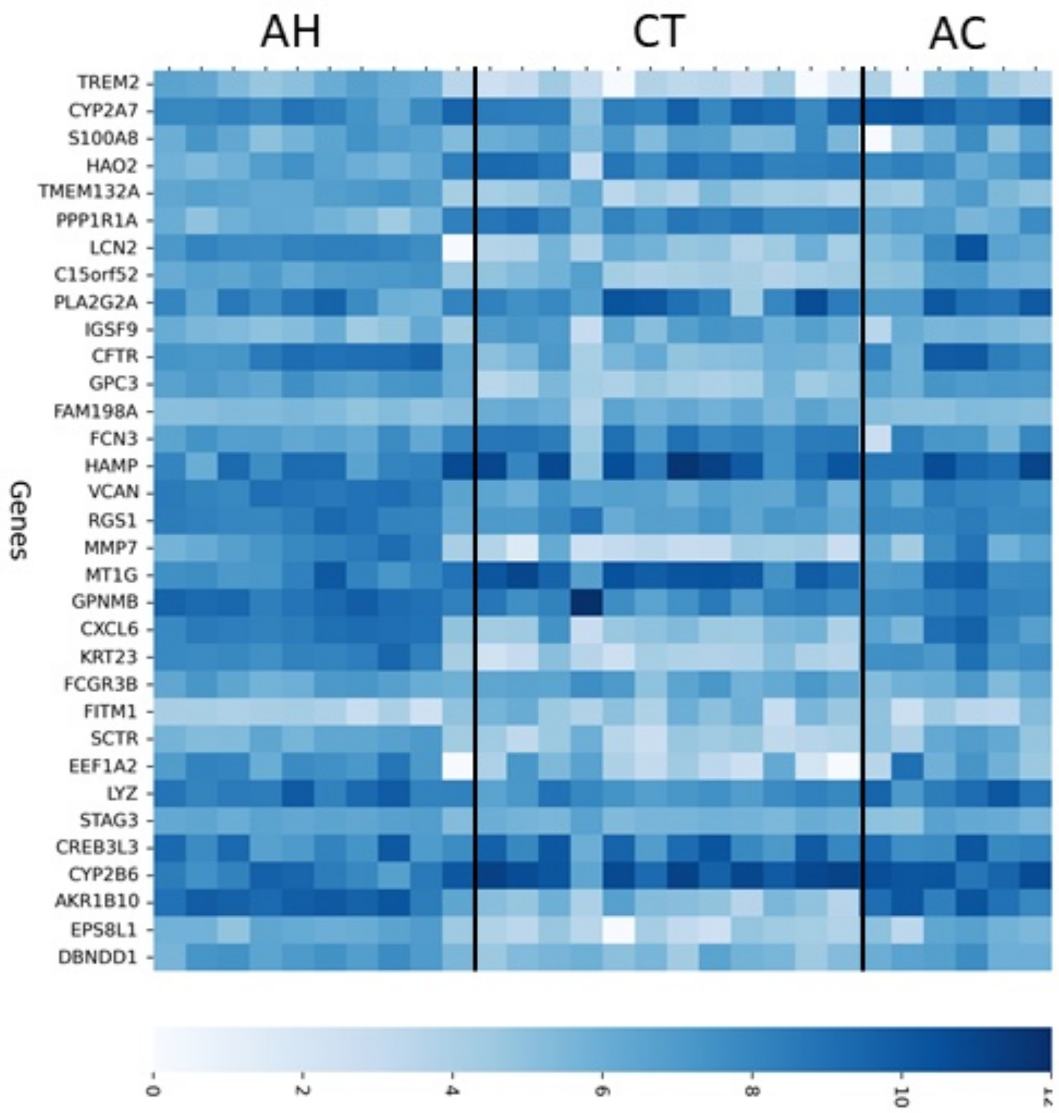

Figure D in S1 Text: Heatmap of RNAseq counts for independent liver validation dataset.

Enrichr:

Table E in S1 Text: Top Enrichr hits for Liver 3-Way Full dataset (RNAseq).

Pathway		
Term	Adjusted P-Value	Genes
Oncostatin M	1.81e-02	CXCL6;AKR1B10;LCN2;HAMP;S100A8
IL-17 signaling pathway	2.20e-02	CXCL6;LCN2;S100A8
Endogenous Toll-like receptor signaling	2.59e-02	VCAN;S100A8
Tissue		
HEPATOCYTE	2.53e-07	FCN3;PLA2G2A;SCTR;FITM1;KRT23;TREM2;IGSF9;FAM198A;DBNDD1;CYP2A7;AKR1B10;CYP2B6;PPP1R1A;CREB3L3;LCN2;GPC3;MT1G;HAO2
LIVER (BULK TISSUE)	4.02e-05	FCN3;PLA2G2A;SCTR;FITM1;IGSF9;FAM198A;CYP2A7;AKR1B10;CYP2B6;CREB3L3;GPC3;MT1G;HAMP;HAO2;CFTR
OMENTUM	1.06e-04	CXCL6;FCN3;MMP7;PLA2G2A;TREM2;IGSF9;FAM198A;PPP1R1A;GPNMB;RGS1;GPC3;MT1G;EPS8L1;S100A8
Disease		
Alcoholic Hepatitis human	8.50e-09	CXCL6;VCAN;MMP7;AKR1B10;GPNMB;PLA2G2A;EEF1A2;LCN2;KRT23;TREM2
hepatocellular carcinoma human	4.53e-05	CYP2A7;CXCL6;FCN3;MMP7;PPP1R1A;MT1G;HAMP;S100A8
Carcinoma, Hepatocellular human	9.65e-05	FCN3;CYP2B6;PPP1R1A;MT1G;HAMP;HAO2;S100A8

ii. PROTEOMIC SECTION for Liver 3-Way Full dataset

Classification Accuracies:

Logistic Regression:

DE None:

Table F in S1 Text: The classification performances of k-nearest neighbors model across a range of feature sizes (15-200) and imputation techniques (median and zero based methods with imputation thresholds of 0, 0.05, 0.1). The features were selected using differential expression.

	Median 0	Median 0.05	Median 0.1	Zero 0	Zero 0.05	Zero 0.1
15	1	0.98	0.98	1	0.98	0.98
25	0.98	0.98	0.98	0.98	0.98	0.98
35	1	1	1	1	1	1
50	1	1	1	1	1	1
60	0.98	1	1	0.98	1	1
70	0.98	0.98	0.98	0.98	0.98	0.98
80	1	1	1	1	1	1
90	1	1	1	1	1	1
100	1	1	1	1	1	1
150	1	1	1	1	1	1
200	1	1	1	1	1	1

Biological Validation (in-silico):

Note that DE used unimputed data to pick top proteins. Therefore, there will be no difference in validation between different imputation methods.

FS Test = 3

DE None:

Table G in S1 Text: The AGOTOOL hits 3 out of 5 Merge configuration in LV 3-Way dataset. The features were selected using differential expression.

	0	0.05	0.1
15 – 11/12/12	7/1/0	7/1/0	7/1/0
25 – 19/19/19	15/2/0	12/2/0	12/2/0
35 – 27/26/26	22/4/1	22/4/2	22/4/2
50 – 40/40/40	17/4/2	17/4/2	17/4/2
60 – 50/51/51	18/3/2	18/3/2	18/3/2
70 – 56/57/57	24/4/2	24/4/2	24/4/2
80 – 62/62/62	24/4/2	23/4/2	23/4/2
90 – 69/69/69	31/4/2	27/4/2	27/4/2
100 – 78/78/78	35/2/2	35/2/2	35/2/2
150 – 117/118/118	44/4/2	45/4/2	45/4/2
200 – 149/149/149	54/4/2	54/4/2	54/4/2

Best protein set for Liver 3-Way Full dataset:
ACBP_HUMAN, ADH1A_HUMAN, ADH1B_HUMAN, ADH4_HUMAN, ADH6_HUMAN, ALBU_HUMAN, ARF3_HUMAN, CD34_HUMAN, CO1A2_HUMAN, CP1A2_HUMAN, CP3A4_HUMAN, CP3A7_HUMAN, CRP_HUMAN, DDTL_HUMAN, ERI3_HUMAN, FABPL_HUMAN, GSTA1_HUMAN, GSTA2_HUMAN, GSTM4_HUMAN, H2B1C_HUMAN, K2C79_HUMAN, K2C80_HUMAN, LDH6A_HUMAN, MFAP4_HUMAN, PAL4C_HUMAN, SAA1_HUMAN, UDB17_HUMAN – 27 proteins.

Heatmaps for Liver 3-Way Full Proteomic:

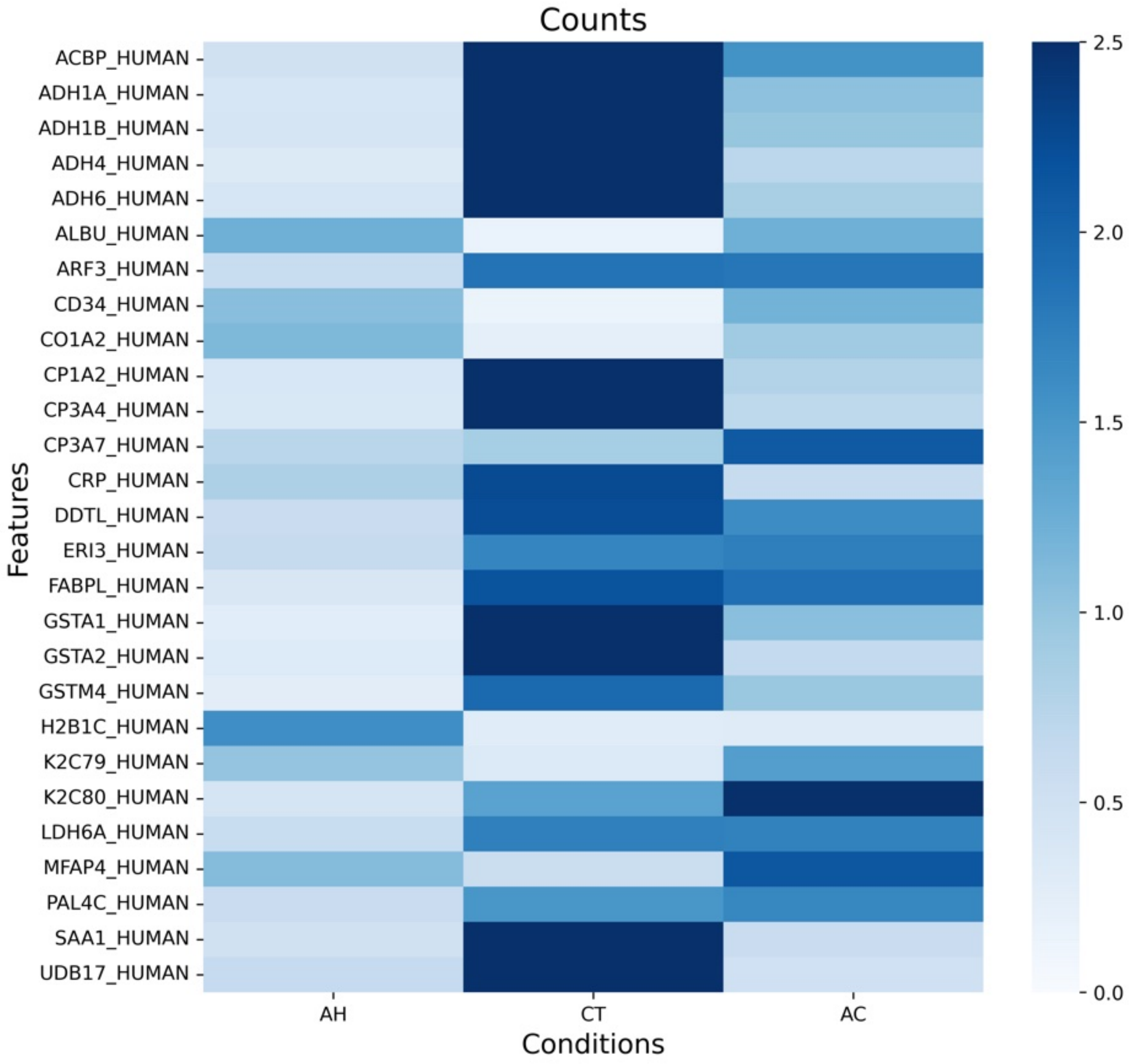

Figure E in S1 Text: Heatmap of proteomic counts for Liver 3-Way Full dataset averaged per condition.

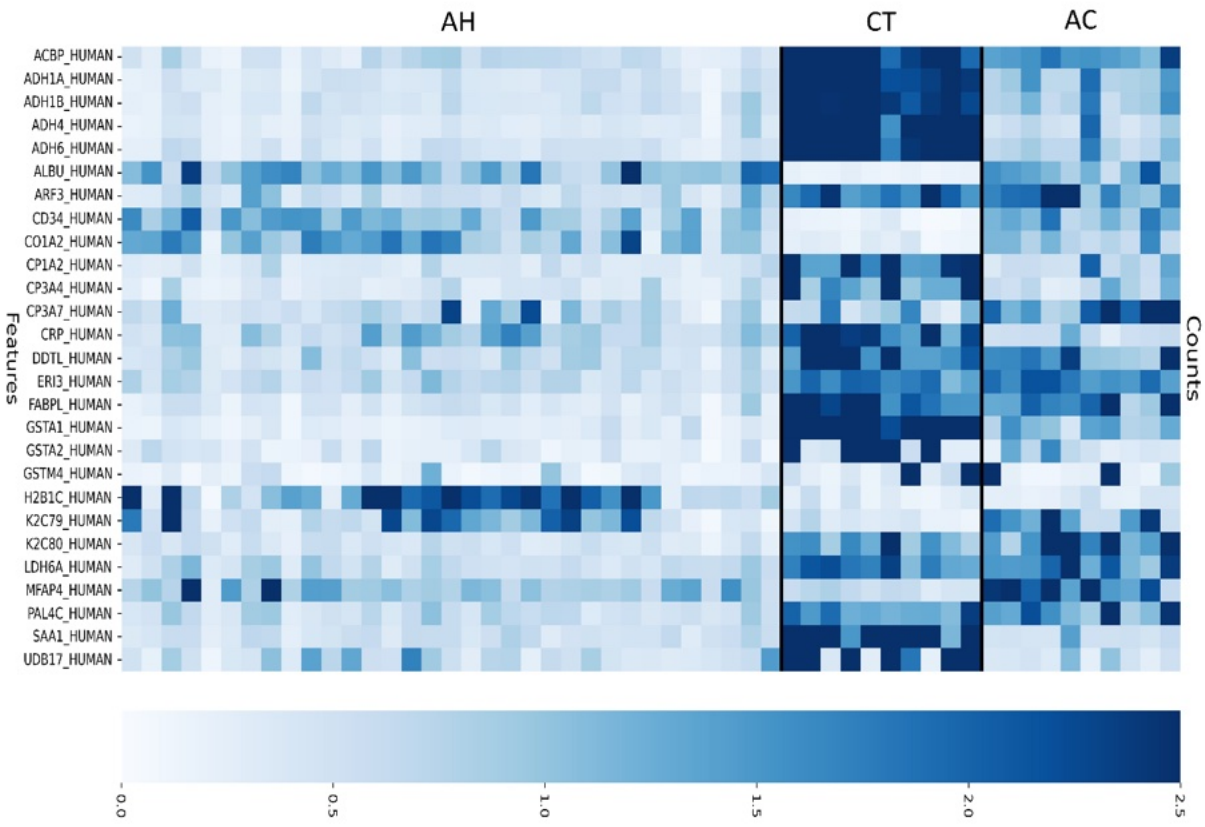

Figure F in S1 Text: Heatmap of proteomic counts for Liver 3-Way Full dataset.

Heatmaps for Liver 3-Way Full Proteomic external validation dataset:

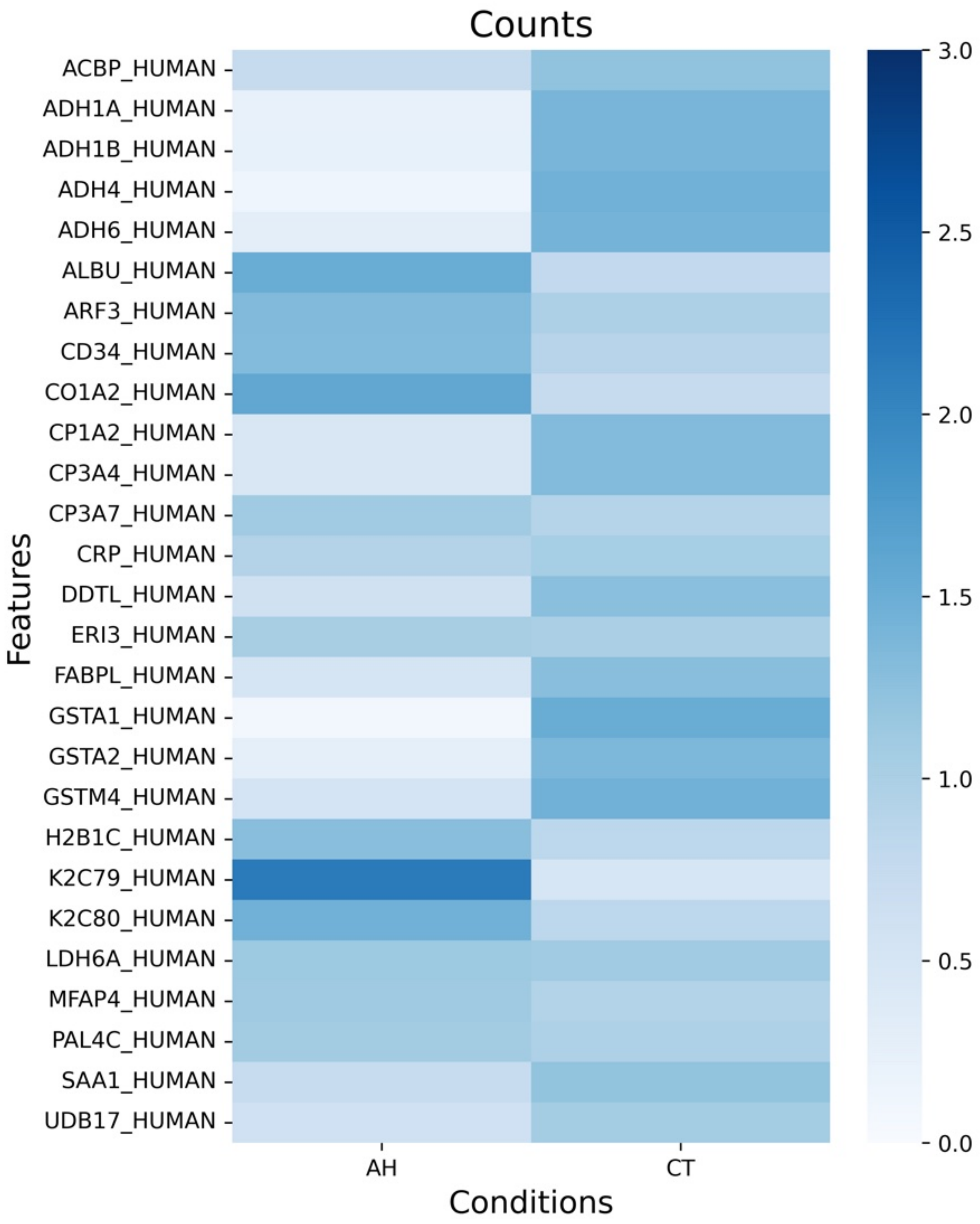

Figure G in S1 Text: Heatmap of proteomic counts for independent liver validation dataset averaged per condition.

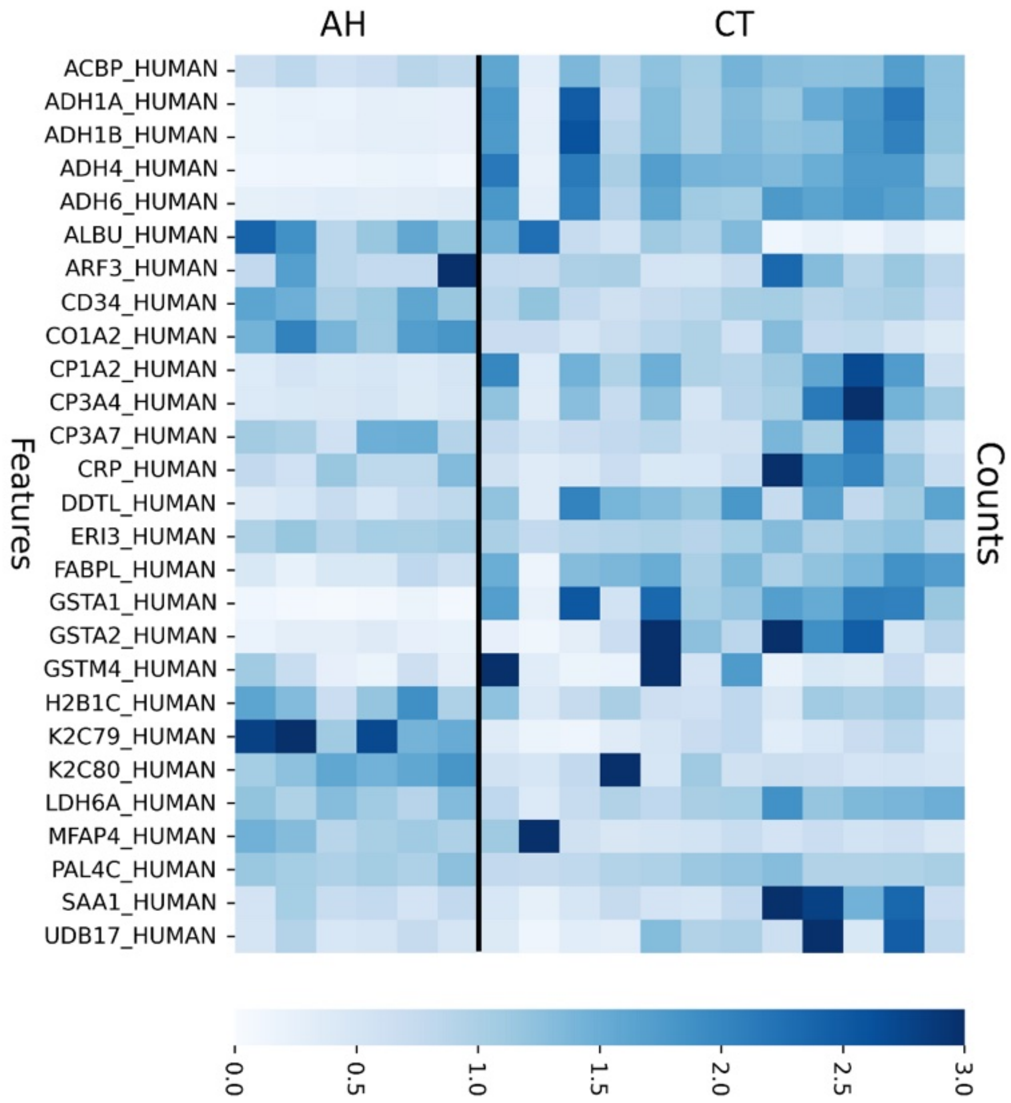

Figure H in S1 Text: Heatmap of proteomic counts for independent liver validation dataset.

AGOTOOL:

Table H in S1 Text: Top AGOTOOL hits for Liver 3-Way Full dataset (Proteomics).

Pathway		
Term	Adjusted P-Value	Proteins
Drug metabolism - cytochrome P450	1.29352e-05	ADH1A_HUMAN;ADH1B_HUMAN;ADH4_HUMAN;ADH6_HUMAN;CP1A2_HUMAN;CP3A4_HUMAN;GSTA1_HUMAN;GSTA2_HUMAN;GSTM4_HUMAN;UDB17_HUMAN
Tyrosine metabolism	1.29352e-05	ADH1A_HUMAN;ADH1B_HUMAN;ADH4_HUMAN;ADH6_HUMAN
Steroid hormone biosynthesis	5.97799e-05	CP1A2_HUMAN;CP3A4_HUMAN;CP3A7_HUMAN;UDB17_HUMAN
Tissue		
Liver	0.00106099	ACBP_HUMAN;ADH1A_HUMAN;ADH1B_HUMAN;ADH4_HUMAN;ADH6_HUMAN;ALBU_HUMAN;CO1A2_HUMAN;CP1A2_HUMAN;CP3A4_HUMAN;CRP_HUMAN;FABPL_HUMAN;GSTA1_HUMAN;GSTA2_HUMAN;SAA1_HUMAN;UDB17_HUMAN
Venous blood	0.0101731	ALBU_HUMAN;CRP_HUMAN
Hepatocyte	0.0273589	ALBU_HUMAN;CP3A4_HUMAN
Disease		
Alcohol dependence	0.0328179	ADH1B_HUMAN;ADH4_HUMAN

c. PBMC 3-Way Full (AH vs Healthy vs AC)

i. TRANSCRIPTOMIC SECTION for PBMCs 3-Way Full dataset:

Classification Accuracies:

LR / Union:

Table I in S1 Text: The classification performances of logistic regression model across a range of feature sizes (10-500) and feature selection techniques (differential expression and information gain with standard deviation thresholds of 2.5, 3.0, 3.5). The accuracy corresponding to best gene set is highlighted in green.

Feature Size	DE 2.5	DE 3	DE 3.5	IG 2.5	IG 3	IG 3.5
10	0.65	0.69	0.74	0.78	0.75	0.77
25	0.77	0.77	0.77	0.84	0.85	0.82
50	0.81	0.80	0.81	0.83	0.81	0.81
100	0.76	0.81	0.83	0.86	0.86	0.80
150	0.85	0.82	0.82	0.86	0.84	0.82
200	0.81	0.79	0.82	0.85	0.83	0.83
250	0.83	0.80	0.82	0.88	0.88	0.82
300	0.82	0.81	0.82	0.86	0.82	0.84
350	0.80	0.82	0.83	0.84	0.84	0.83
400	0.81	0.80	0.83	0.86	0.83	0.86
450	0.82	0.83	0.84	0.83	0.85	0.89
500	0.79	0.84	0.83	0.84	0.85	0.86

Enrichr In Silico Biological Validation:

Union / 4 out of 5 Merge:

Table J in S1 Text: The Enrichr hits Union / 4 out of 5 Merge configuration in PBMC 3-Way dataset.

Feature Size	DE 2.5	DE 3	DE 3.5	IG 2.5	IG 3	IG 3.5
10 – 5/8/7/2/3/1	12/4/4	25/3/5	24/4/5	0/1/1	8/1/0	3/1/1
25 – 10/17/19/5/8/6	26/4/14	19/6/6	18/4/7	10/2/1	15/2/0	13/3/1
50 – 20/32/38/16/20/16	45/5/13	35/6/15	15/6/16	82/6/3	7/4/1	12/4/3
100 – 43/65/78/33/48/36	15/7/11	30/11/16	33/11/15	20/6/3	11/9/4	14/7/5
150 – 69/102/114/48/75/49	24/8/15	48/12/20	48/12/21	20/5/3	35/10/6	16/8/6
200 – 99/142/152/67/106/74	40/9/15	66/14/19	50/12/21	15/5/12	26/10/6	26/9/5
250 – 117/176/192/85/135/91	41/9/13	68/14/22	74/13/21	14/5/11	22/10/8	36/10/6
300 – 140/204/229/111/164/102	52/11/13	58/14/22	69/14/22	14/5/10	24/11/14	38/11/7

Best gene set for PBMC 3-Way Full dataset: ETS2, FLVCR2, FPR1, GRB10, IMPA2, ITGAM, ITGB2, LILRA5, MYO7A, PTGR1, RAB31, RNASE2, SERPINB1, SLC36A1, ST14, TLR4 – 16 genes.

Heatmaps for PBMC 3-Way Full RNAseq dataset:

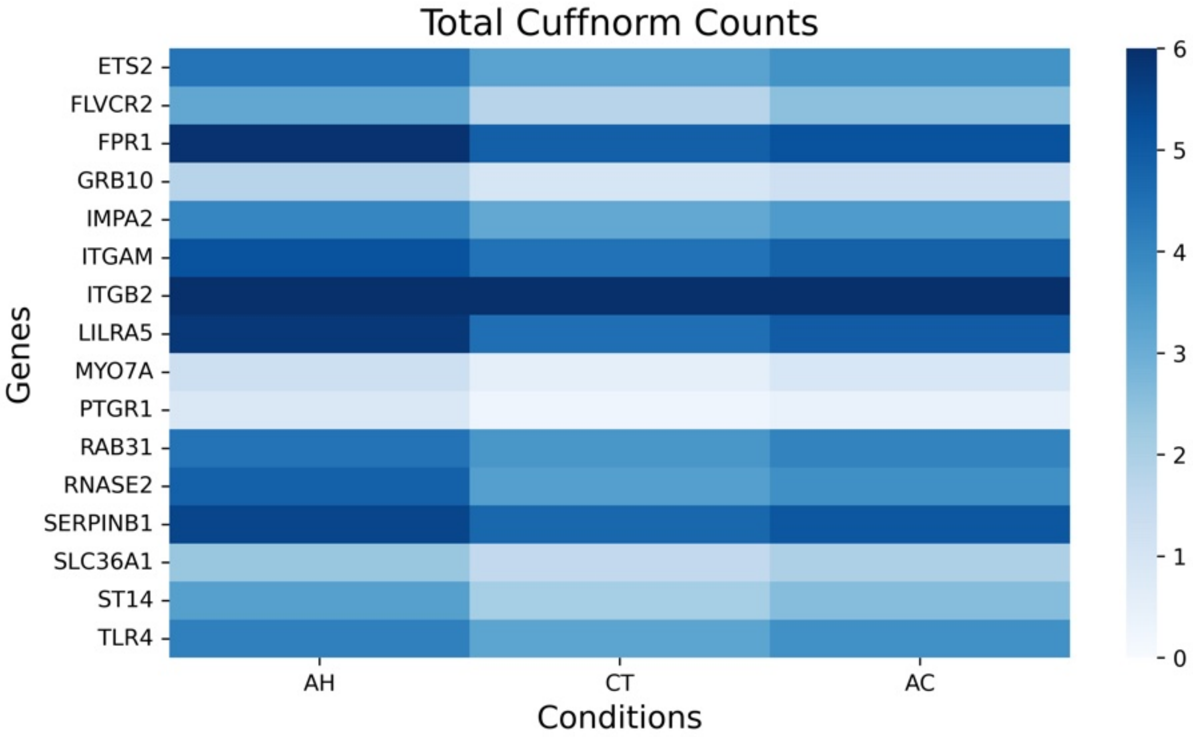

Figure I in S1 Text: Heatmap of RNAseq counts for PBMC 3-Way Full dataset averaged per condition.

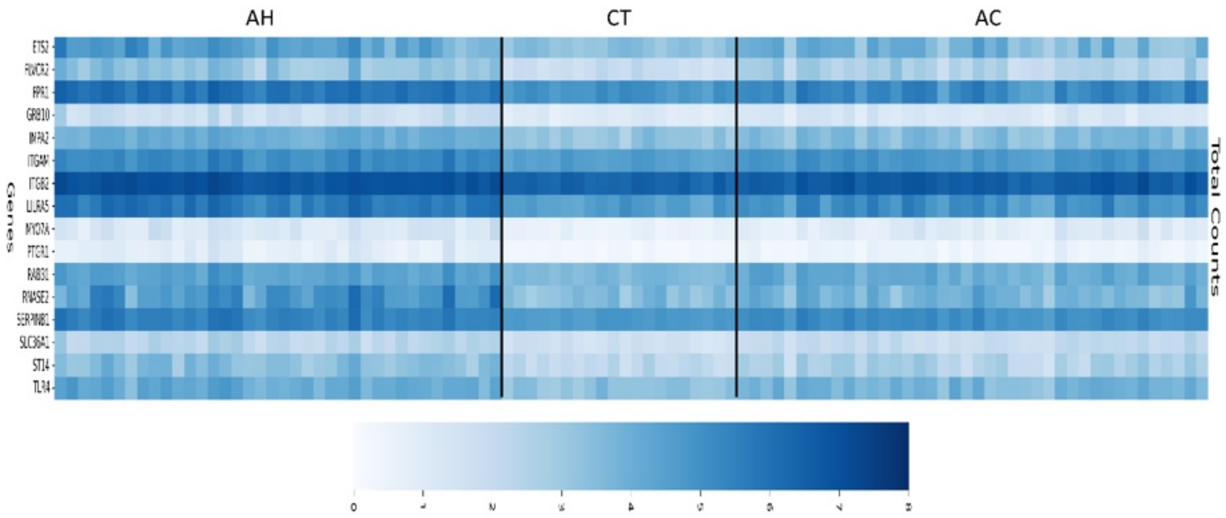

Figure J in S1 Text: Heatmap of RNAseq counts for PBMC 3-Way Full dataset.

Enrichr:

Table K in S1 Text: Top Enrichr hits for PBMC 3-Way Full dataset (RNAseq).

Pathway		
Term	Adjusted P-Value	Genes
toll-like receptor 4 signaling pathway	5.12e-05	ITGAM;ITGB2;TLR4
neutrophil degranulation	8.87e-05	SERPINB1;ITGAM;RAB31;ITGB2;FPR1;RNASE2
Interleukin-2 signaling pathway	3.71e-03	ITGAM;RAB31;ITGB2;TLR4;ETS2
Tissue		
MACROPHAGE	9.46e-06	SLC36A1;ST14;ITGAM;RAB31;ITGB2;FPR1;FLVCR2;MYO7A;RNASE2;TLR4;LILRA5
PERIPHERAL BLOOD	3.78e-04	SLC36A1;ST14;ITGAM;ITGB2;FPR1;FLVCR2;RNASE2;TLR4;LILRA5
CD14+ Monocytes	1.80e-03	SERPINB1;RAB31;FPR1;LILRA5
Disease		
Septic Shock human	4.14e-07	SERPINB1;RAB31;FPR1;GRB10;RNASE2;TLR4;ETS2;LILRA5
familial combined hyperlipidemia human	1.26e-05	IMPA2;ITGB2;FPR1;RNASE2;TLR4;ETS2
familial hypercholesterolemia human	2.45e-03	ITGAM;ITGB2;FPR1;RNASE2

ii. PROTEOMIC SECTION for PBMC 3-Way Full dataset:

Classification Accuracies:

Logistic Regression:

DE Variance (2.5):

Table L in S1 Text: The classification performances of logistic regression model across a range of feature sizes (15-200) and imputation techniques (median and zero based methods with imputation thresholds of 0, 0.05, 0.1). The features were selected using differential expression and variance 2.5 filter.

	Median 0	Median 0.05	Median 0.1	Zero 0	Zero 0.05	Zero 0.1
15	0.8	0.8	0.8	0.8	0.8	0.8
25	0.85	0.85	0.85	0.85	0.85	0.85
35	0.87	0.87	0.87	0.87	0.87	0.87
50	0.87	0.87	0.87	0.87	0.87	0.87
60	0.86	0.86	0.89	0.86	0.86	0.86
70	0.85	0.85	0.86	0.85	0.85	0.85
80	0.87	0.87	0.87	0.87	0.87	0.87
90	0.87	0.87	0.87	0.87	0.87	0.87
100	0.87	0.87	0.86	0.87	0.87	0.86
150	0.87	0.87	0.87	0.87	0.87	0.87
200	0.85	0.85	0.85	0.85	0.85	0.87

Biological Validation (in-silico):

FS Test = 4:

DE Variance (2.5):

Table M in S1 Text: The AGOTOOL hits 4 out of 5 Merge configuration in PBMC 3-Way dataset. The features were selected using differential expression and variance 2.5 filter.

	0	0.05	0.1
15 - 3/3/3	11/0/0	11/0/0	11/0/0
25 - 7/7/7	7/2/0	7/2/0	7/2/0
35 - 15/15/15	17/2/0	17/2/0	17/2/0
50 - 20/20/20	14/3/0	14/3/0	14/3/0
60 - 28/28/28	23/4/0	23/4/0	23/4/0
70 - 32/32/32	23/3/0	23/3/0	23/3/0
80 - 38/38/38	27/5/0	27/5/0	27/5/0
90 - 43/43/43	25/7/0	25/7/0	25/7/0
100 - 49/49/49	26/6/0	26/6/0	26/6/0
150 - 76/76/75	26/8/0	26/8/0	26/8/0
200 - 106/106/105	37/9/0	37/9/0	34/9/0

Best protein set for PBMC 3-Way Full dataset: APOA1_HUMAN, BLVRB_HUMAN, CATS_HUMAN, CSRP1_HUMAN, EST1_HUMAN, FIBA_HUMAN, FIBB_HUMAN, FIBG_HUMAN, G6B_HUMAN, GP1BB_HUMAN, GPIX_HUMAN, HBD_HUMAN, ILK_HUMAN, ITA2B_HUMAN, ITB3_HUMAN, LTBP1_HUMAN, MYL9_HUMAN, PMGE_HUMAN, RAP1A_HUMAN, RSU1_HUMAN, SDPR_HUMAN, SEP11_HUMAN, SRC_HUMAN, TBA4A_HUMAN, TOR4A_HUMAN, TSP1_HUMAN, URP2_HUMAN, VINC_HUMAN – 28 proteins.

Heatmaps for PBMC 3-Way Full Proteomics dataset:

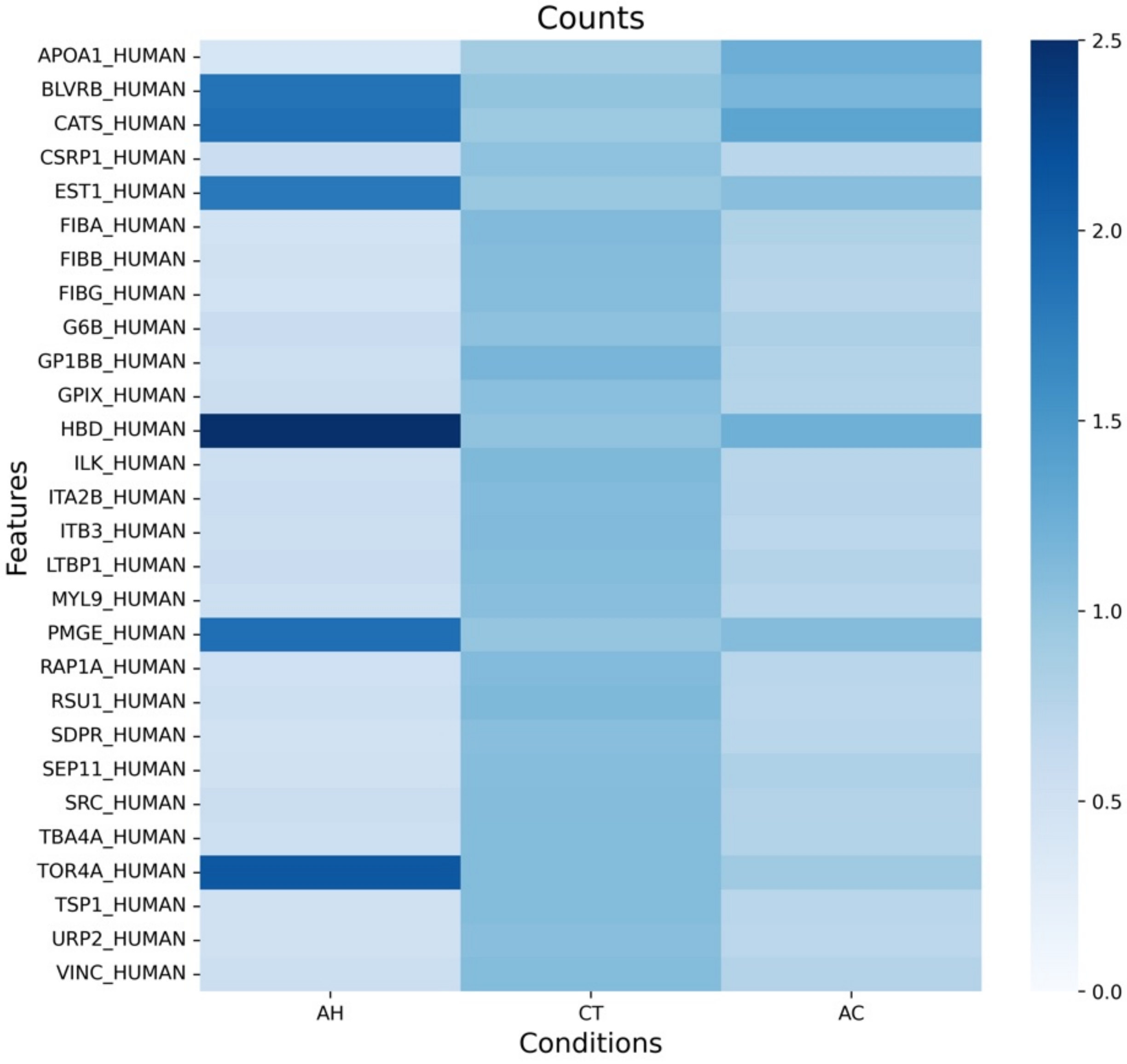

Figure K in S1 Text: Heatmap of proteomic counts for PBMC 3-Way dataset averaged per condition.

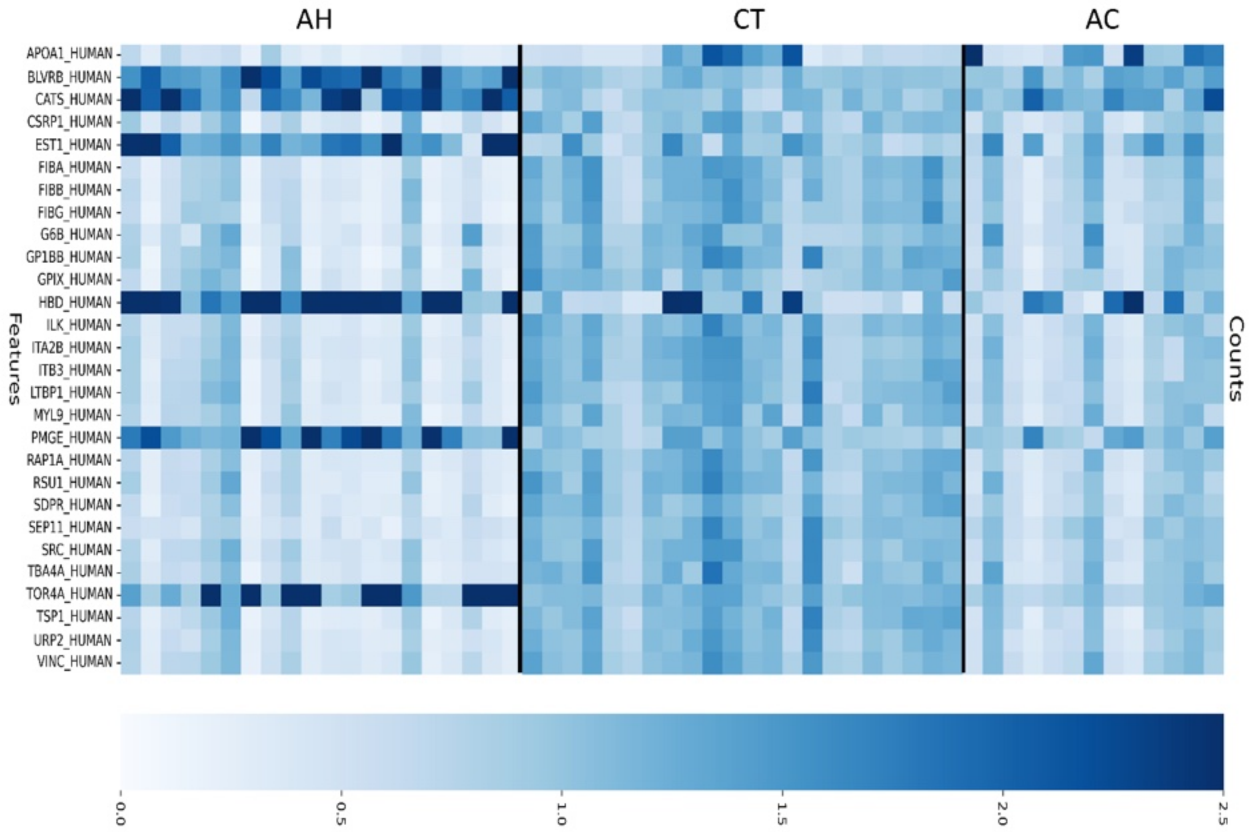

Figure L in S1 Text: Heatmap of proteomic counts for PBMC 3-Way Full dataset.

AGOTOOL:

Table N in S1 Text: Top AGOTOOL hits for PBMC 3-Way Full dataset (Proteomics).

Pathway		
Term	Adjusted P-Value	Proteins
Platelet activation	1.21e-4	FIBA_HUMAN;FIBB_HUMAN;FIBG_HUMAN;GP1BB_HUMAN;GPIX_HUMAN;ITA2B_HUMAN;ITB3_HUMAN;RAP1A_HUMAN;SRC_HUMAN;URP2_HUMAN
Complement system	2.13e-4	APOA1_HUMAN;FIBA_HUMAN;FIBB_HUMAN;FIBG_HUMAN;ITA2B_HUMAN;ITB3_HUMAN;TSP1_HUMAN
Fibrin complement receptor 3 signaling pathway	2.13e-4	FIBA_HUMAN;FIBB_HUMAN;FIBG_HUMAN;SRC_HUMAN
Tissue		
Blood plasma	2.16e-4	APOA1_HUMAN;FIBA_HUMAN;FIBB_HUMAN;FIBG_HUMAN;G6B_HUMAN;GP1BB_HUMAN;GPIX_HUMAN;ITA2B_HUMAN;ITB3_HUMAN;LTBP1_HUMAN;RAP1A_HUMAN;RSU1_HUMAN;SEP11_HUMAN;SRC_HUMAN;TBA4A_HUMAN;TSP1_HUMAN;URP2_HUMAN;VINC_HUMAN
Blood	2.37e-4	APOA1_HUMAN;BLVRB_HUMAN;CATS_HUMAN;EST1_HUMAN;FIBA_HUMAN;FIBB_HUMAN;FIBG_HUMAN;G6B_HUMAN;GP1BB_HUMAN;GPIX_HUMAN;HBD_HUMAN;ITA2B_HUMAN;ITB3_HUMAN;LTBP1_HUMAN;RAP1A_HUMAN;RSU1_HUMAN;SEP11_HUMAN;SRC_HUMAN;TBA4A_HUMAN;TSP1_HUMAN;URP2_HUMAN;VINC_HUMAN
Liver	1.52e-2	APOA1_HUMAN;BLVRB_HUMAN;CATS_HUMAN;EST1_HUMAN;FIBA_HUMAN;FIBB_HUMAN;FIBG_HUMAN;SRC_HUMAN;TBA4A_HUMAN;TSP1_HUMAN;VINC_HUMAN
Disease		

c. Liver 3-Way Unmatched Balanced (AH vs Healthy vs AC)

i. TRANSCRIPTOMIC SECTION for Liver 3-Way Unmatched Balanced dataset:

Classification Performance:

SVM / Union:

Table O in S1 Text: The classification performances of support vector machine model across a range of feature sizes (10-500) and feature selection techniques (differential expression and information gain with standard deviation thresholds of 2.5, 3.0, 3.5). The accuracy corresponding to best gene set is highlighted in green.

Feature Size	DE 2.5	DE 3	DE 3.5	IG 2.5	IG 3	IG 3.5
10	0.55	0.55	0.55	0.66	0.68	0.61
25	0.66	0.66	0.66	0.66	0.6	0.61
50	0.55	0.55	0.55	0.61	0.55	0.56
100	0.55	0.55	0.55	0.61	0.55	0.61
150	0.55	0.55	0.55	0.5	0.61	0.5
200	0.71	0.71	0.71	0.5	0.5	0.5
250	0.6	0.6	0.6	0.56	0.55	0.55
300	0.61	0.61	0.61	0.5	0.55	0.55
350	0.61	0.61	0.61	0.6	0.5	0.55
400	0.61	0.61	0.61	0.6	0.55	0.5
450	0.61	0.61	0.61	0.5	0.56	0.5
500	0.66	0.66	0.66	0.55	0.61	0.61

Enrichr In Silico Biological Validation:

Union / 5 out of 5 Merge:

Table P in S1 Text: The Enrichr hits Union / 5 out of 5 Merge configuration in Liver 3-Way Unmatched Balanced dataset.

Feature Size	DE 2.5	DE 3	DE 3.5	IG 2.5	IG 3	IG 3.5
10 – 2/2/2/0/0/0	22/2/18	22/2/18	22/2/18			
25 – 5/5/5/1/0/0	38/2/9	38/2/9	38/2/9	1/0/1		
50 – 9/9/9/2/2/1	25/2/14	25/2/14	25/2/14	1/0/1	1/0/1	1/0/1
100 – 31/31/31/2/3/3	25/8/29	25/8/29	25/8/29	1/0/1	6/0/0	0/0/0
150 – 45/45/45/3/3/7	27/6/24	27/6/24	27/6/24	0/0/0	6/0/0	5/0/0
200 – 59/59/59/7/8/9	28/6/28	28/6/28	28/6/28	5/0/0	11/0/0	6/0/0
250 – 79/79/79/11/18/16	32/6/29	32/6/29	32/6/29	0/0/0	0/0/0	10/0/0
300 – 96/96/96/17/25/21	23/6/32	23/6/32	23/6/32	0/0/0	4/0/0	3/0/0
350 – 120/120/120/26/31/30	25/6/40	25/6/40	25/6/40	3/0/0	6/0/0	3/0/0
400 – 138/138/138/38/38/39	27/6/42	27/6/42	27/6/42	1/0/0	3/0/0	2/0/0
450 – 160/160/160/48/48/45	33/6/45	33/6/45	33/6/45	5/0/0	3/0/0	4/0/0
500 – 180/180/180/60/62/56	31/6/42	31/6/42	31/6/42	5/0/0	4/0/0	2/0/0

Best gene set for Liver 3-Way Unmatched Balanced dataset: ACKR1, AKR1B10, BBOX1, C15orf52, CFTR, CLEC4M, CREB3L3, CSF3R, CXCL1, CXCL6, DCDC2, DHODH, DHRS2, F3, FABP4, FAM118A, FCGR3B, FCN3, GADD45B, GADD45G, GPC3, GSTA2, HAMP, HAO2, ID4, IGSF9, IL7R, KRT23, LBP, LCN2, LRG1, MARCO, MMP7, MT1A, MT1G, MT1H, MT1M, MT1X, MUC13, MUC6, NRTN, PAPLN, PID1, PLA2G2A, PLCB1, PPP1R1A, S100A12, S100A8, S100A9, SLC13A5, SLC22A1, SOCS1, SPINK1, STAG3, STMN2, TREM2, TRIB3, VSIG2, VTCN1 – 59 Genes.

Table Q in S1 Text: Top Enrichr hits for Liver 3-Way Unmatched Balanced dataset (RNAseq).

Term	Adjusted P-Value	Genes
**Pathway**		
Oncostatin M	1.32e-09	CXCL6;SLC22A1;BBOX1;CXCL1;F3;AKR1B10;SOCS1;LCN2;S100A12;MT1H;S100A9;HAMP;S100A8
cellular transition metal ion homeostasis	1.23e-06	MT1A;MT1M;LCN2;MT1G;MT1H;MT1X;HAMP
positive regulation of inflammatory response	4.20e-06	CREB3L3;PLA2G2A;S100A12;LBP;S100A9;S100A8
**Tissue**		
LIVER (BULK TISSUE)	2.08e-14	FCN3;PID1;CLEC4M;SLC22A1;MT1M;CXCL1;MT1X;VTCN1;IGSF9;CREB3L3;GPC3;NRTN;LBP;SLC13A5;HAO2;MUC6;SPINK1;GADD45B;PLA2G2A;BBOX1;DCDC2;DHRS2;DHODH;MARCO;MT1A;AKR1B10;LRG1;GSTA2;MT1G;MT1H;TRIB3;HAMP;CFTR
HEPATOCYTE	7.34e-12	FCN3;PID1;SLC22A1;MT1M;KRT23;TREM2;IGSF9;CREB3L3;GPC3;MUC13;NRTN;LBP;SLC13A5;HAO2;MUC6;SPINK1;GADD45B;PLA2G2A;BBOX1;DCDC2;GADD45G;MT1A;AKR1B10;LRG1;PPP1R1A;GSTA2;LCN2;MT1G;MT1H;TRIB3
OMENTUM	1.21e-08	CXCL6;FCN3;PID1;MT1M;CXCL1;TREM2;MT1X;VTCN1;IGSF9;SOCS1;VSIG2;GPC3;LBP;MMP7;GADD45B;PLA2G2A;GADD45G;MARCO;MT1A;FABP4;PPP1R1A;ID4;MT1G;ACKR1;S100A9;S100A8
**Disease**		
hepatocellular carcinoma human	1.84e-14	CXCL6;FCN3;MMP7;GADD45B;SLC22A1;MT1M;BBOX1;MT1X;MARCO;MT1A;LRG1;PPP1R1A;VSIG2;MT1G;MT1H;S100A9;HAMP;S100A8
alcoholic hepatitis human	6.95e-12	FCN3;CLEC4M;GADD45B;MT1M;BBOX1;MT1X;DHRS2;GADD45G;MARCO;PPP1R1A;MT1G;S100A12;MT1H;HAO2;HAMP
hepatocellular carcinoma human	3.77e-11	FCN3;SLC22A1;MT1M;BBOX1;MT1X;LRG1;PPP1R1A;MT1G;MT1H;LBP;S100A9;HAO2;HAMP;S100A8

Confusion Matrix for Liver 3-Way Unmatched Balanced dataset:

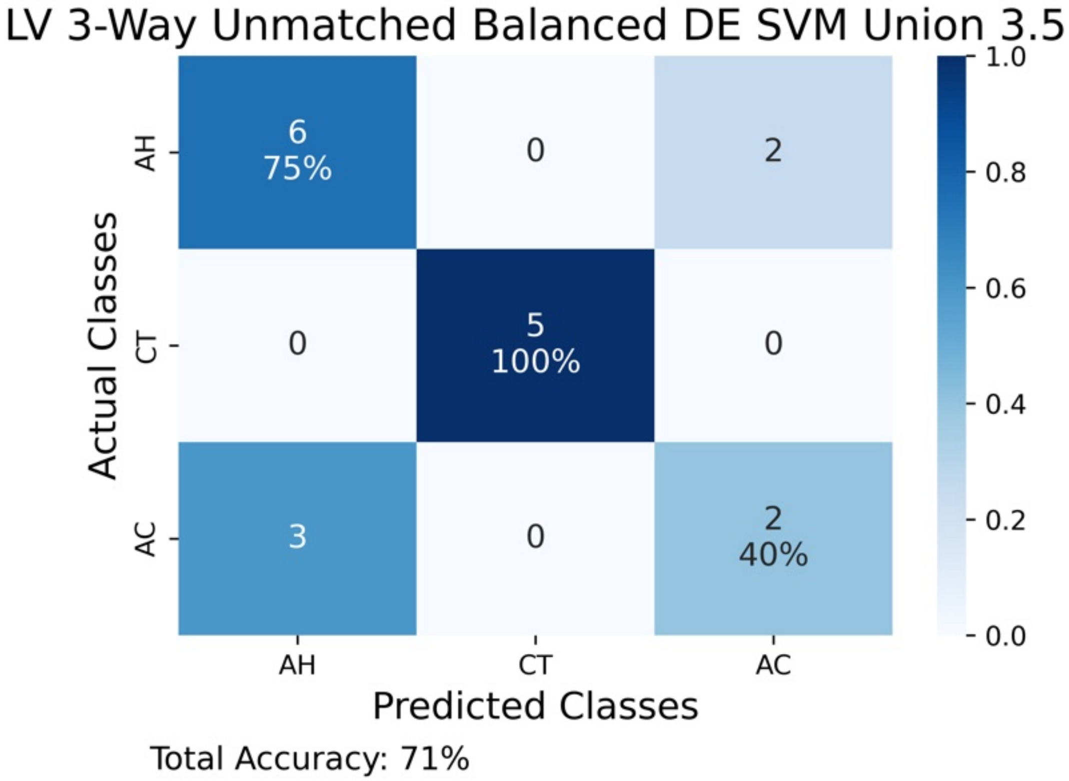

Figure M in S1 Text: Confusion matrix for nested cross-validated classification of Liver 3-Way Unmatched Balanced RNAseq dataset.

Confusion Matrix in Validation Data for Liver 3-Way Unmatched Balanced dataset:

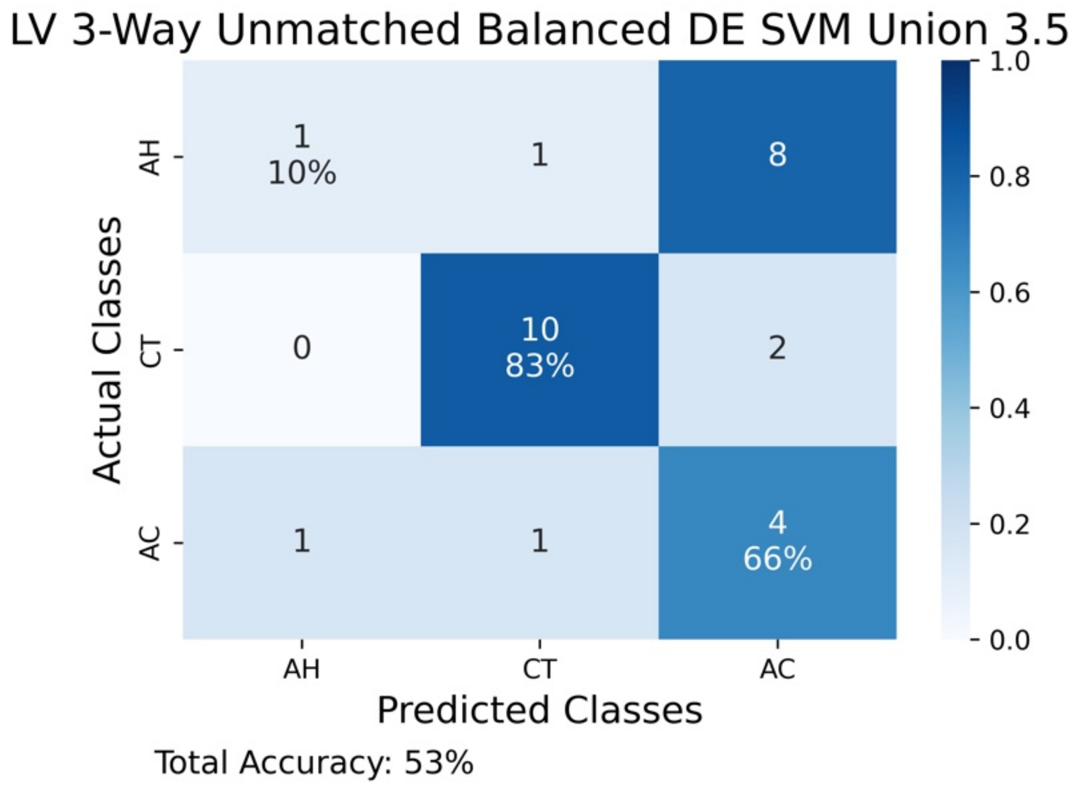

Figure N in S1 Text: Confusion matrix for classification of Liver 3-Way Unmatched Balanced RNAseq dataset in independent validation data.

Heatmaps in Liver 3-Way Unmatched Balanced RNAseq dataset:

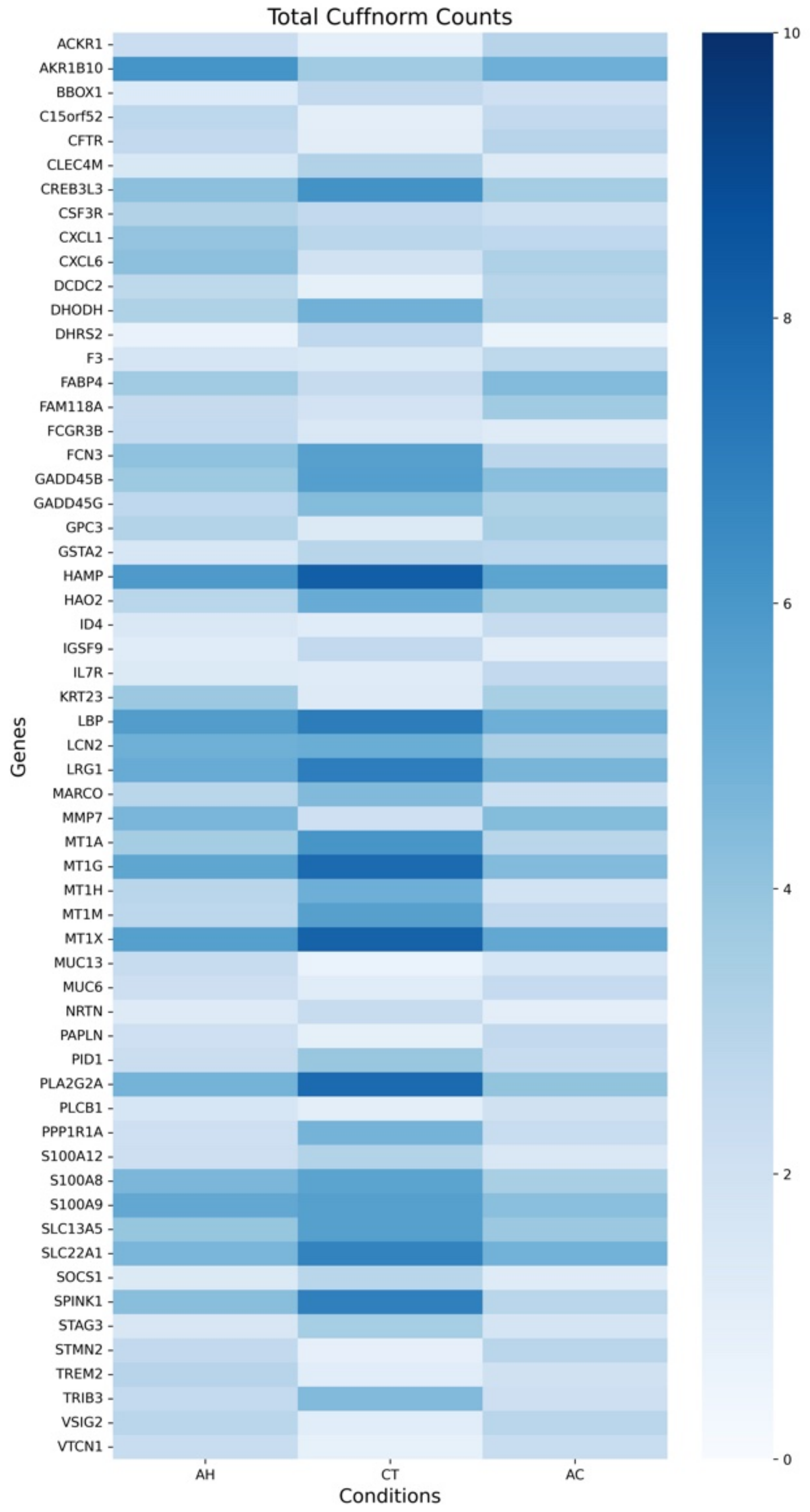

Figure O in S1 Text: Heatmap of RNAseq counts for Liver 3-Way Unmatched Balanced dataset averaged per condition.

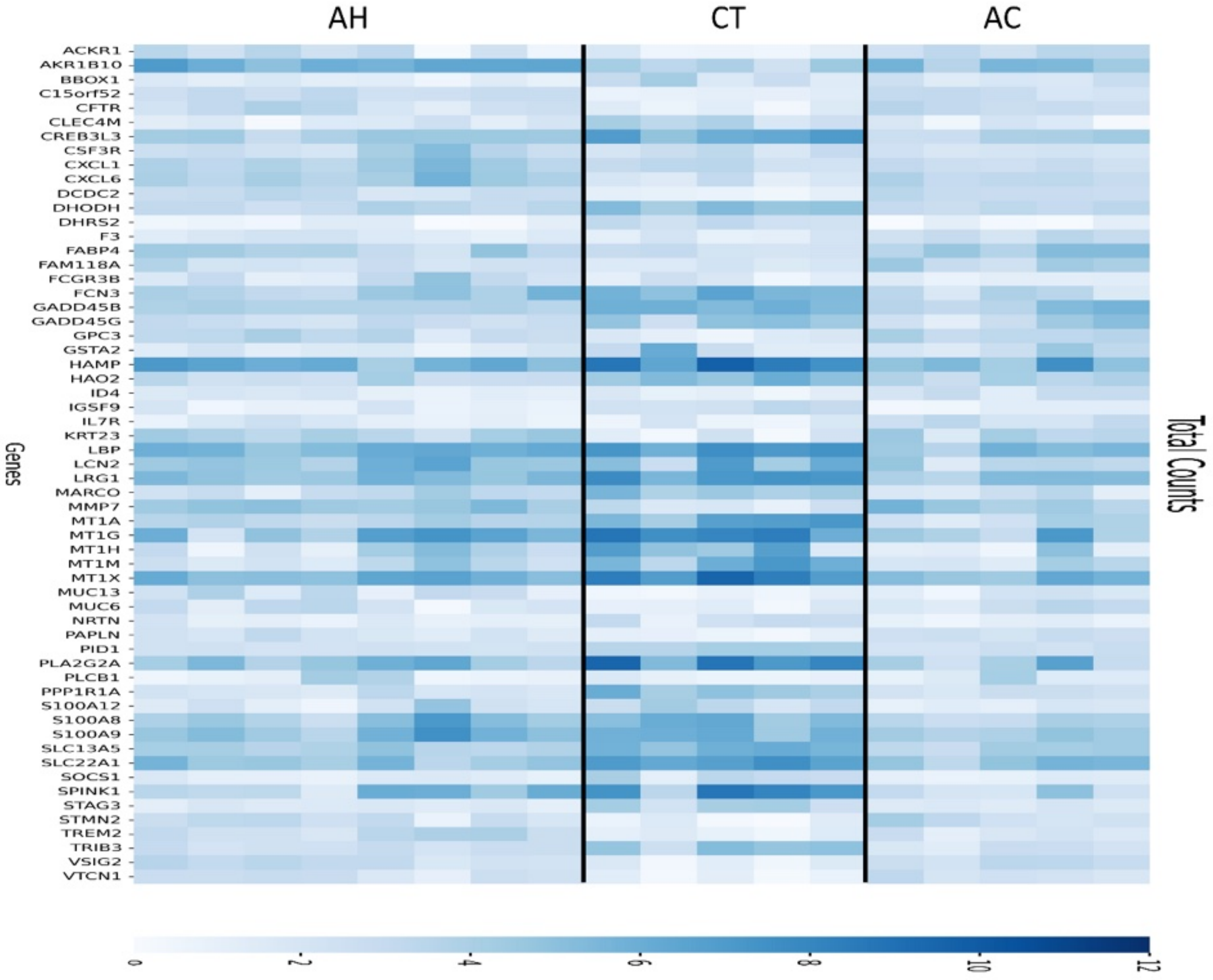

Figure P in S1 Text: Heatmap of RNAseq counts for Liver 3-Way Unmatched Balanced dataset.

Heatmaps in Liver 3-Way Unmatched Balanced RNAseq external validation dataset:

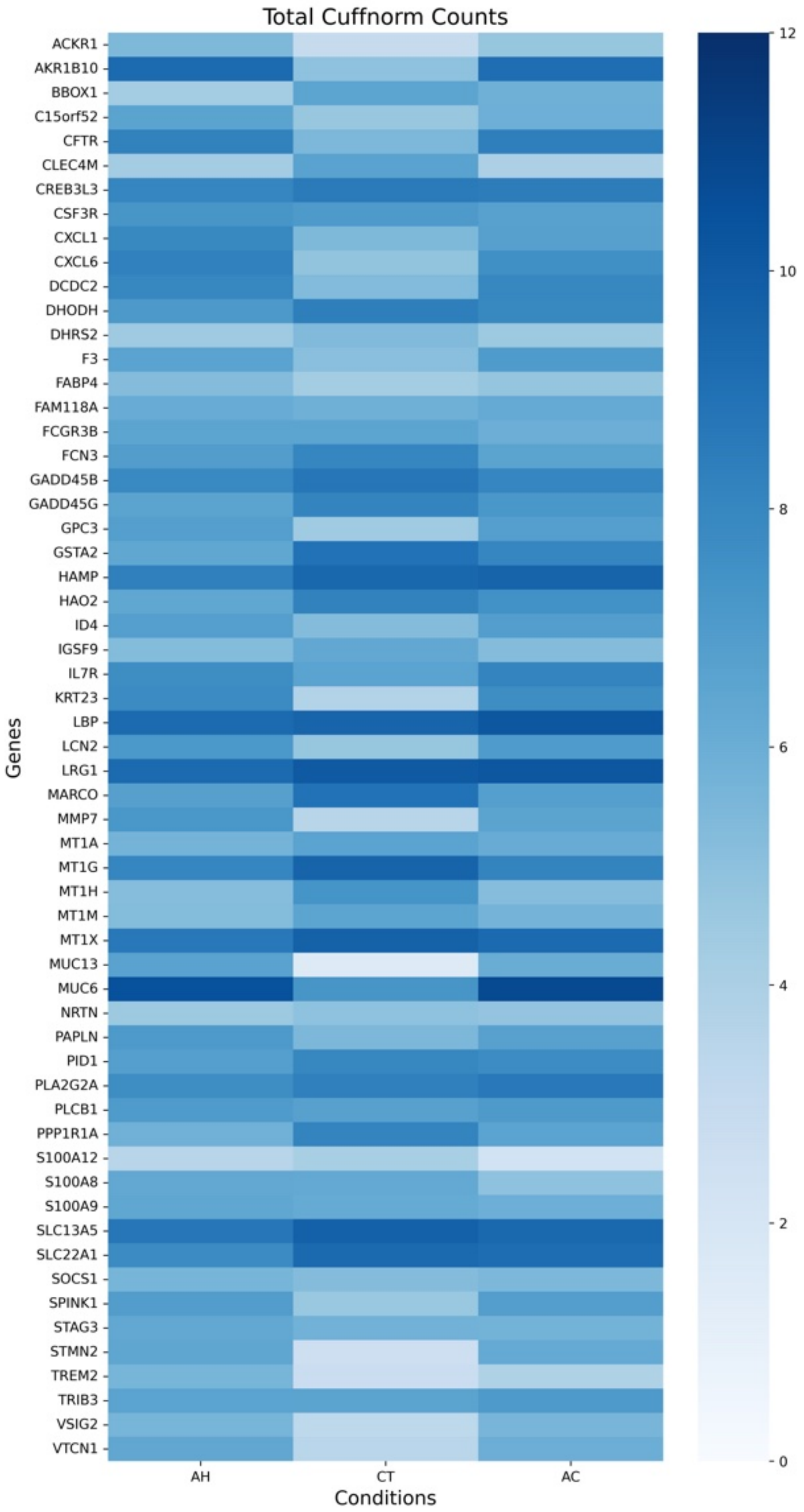

Figure Q in S1 Text: Heatmap of RNAseq counts for independent liver validation dataset averaged per condition.

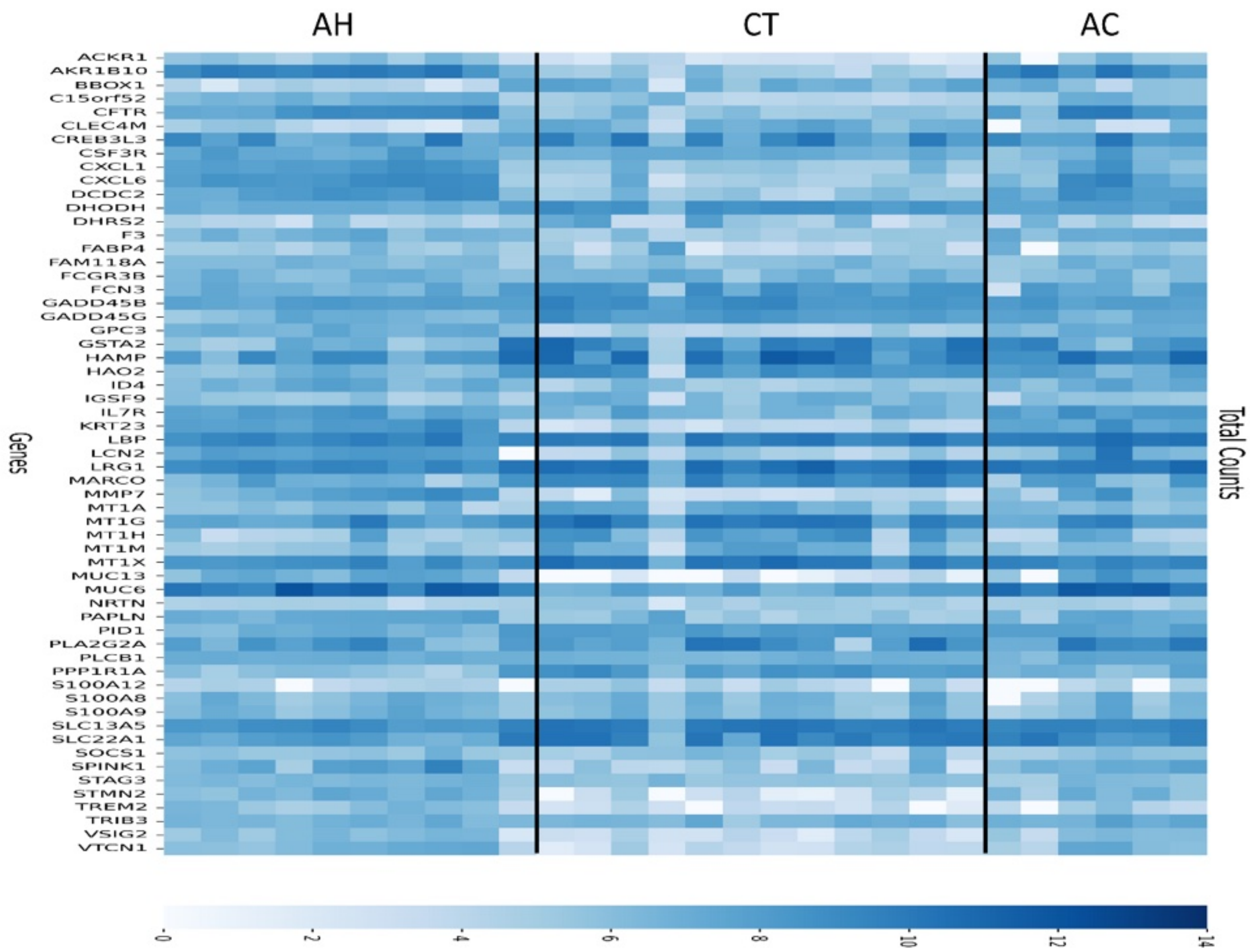

Figure R in S1 Text: Heatmap of RNAseq counts for independent liver validation dataset.

ii. PROTEOMIC SECTION for Liver 3-Way Unmatched Balanced dataset:

Classification Performance:

Support Vector Machine:

DE None:

Table R in S1 Text: The classification performances of support vector machine model across a range of feature sizes (15-200) and imputation techniques (median and zero based methods with imputation thresholds of 0, 0.05, 0.1). The features were selected using differential expression.

	Median 0	Median 0.05	Median 0.1	Zero 0	Zero 0.05	Zero 0.1
15	0.93	0.93	0.93	0.93	0.93	0.93
25	0.97	0.97	0.97	0.97	0.97	0.97
35	0.97	0.97	0.97	0.97	0.97	0.97
50	1	1	1	1	1	1
60	0.97	0.97	0.97	0.97	0.97	0.97
70	0.97	0.97	0.97	0.97	0.97	0.97
80	0.97	0.97	0.97	0.97	0.97	0.97
90	0.97	0.97	0.97	0.97	0.97	0.97
100	0.97	0.97	0.97	0.97	0.97	0.97
150	0.93	0.93	0.93	0.93	0.93	0.93
200	0.93	0.93	0.93	0.93	0.93	0.93

Biological Validation (in-silico):

FS Test = 3:

DE None:

Table S in S1 Text: The AGOTOOL hits 3 out of 5 Merge configuration in LV 3-Way Unmatched Balanced dataset. The features were selected using differential expression.

	0	0.05	0.1
15 - 10/10/10	6/1/0	6/1/0	6/1/0
25 - 14/14/14	11/4/1	11/4/1	11/4/1
35 - 19/19/19	13/4/3	13/4/3	13/4/3
50 - 33/33/33	16/4/2	16/4/2	16/4/2
60 - 41/41/41	25/4/1	25/4/1	25/4/1
70 - 45/45/45	24/4/1	24/4/1	24/4/1
80 - 51/51/51	25/4/2	25/4/2	25/4/2
90 - 58/58/58	30/4/2	30/4/2	30/4/2
100 - 66/65/65	35/4/2	36/4/2	36/4/2
150 - 103/103/103	48/2/2	48/2/2	48/2/2
200 - 145/145/145	65/3/3	65/3/3	65/3/3

Best protein set for Liver 3-Way Unmatched Balanced dataset: ACBP_HUMAN, ADH1A_HUMAN, ADH1B_HUMAN, ADH4_HUMAN, ADH6_HUMAN, ALBU_HUMAN, ASSY_HUMAN, CD34_HUMAN, CLC4M_HUMAN, CO1A2_HUMAN, CP1A2_HUMAN, CRP_HUMAN, CYB5_HUMAN, ERI3_HUMAN, GSTA1_HUMAN, HBAZ_HUMAN, LDH6A_HUMAN, SAA1_HUMAN, UDB17_HUMAN – 19 proteins.

Table T in S1 Text: Top AGOTOOL hits for Liver 3-Way Unmatched Balanced dataset (Proteomics).

Pathway		
Term	Adjusted P-Value	Proteins
Tyrosine metabolism	9.05e-06	ADH1A_HUMAN;ADH1B_HUMAN;ADH4_HUMAN;ADH6_HUMAN
Retinol metabolism	4.40e-05	ADH1A_HUMAN;ADH1B_HUMAN;ADH4_HUMAN;ADH6_HUMAN;CP1A2_HUMAN;UDB17_HUMAN
Drug metabolism - cytochrome P450	4.44e-05	ADH1A_HUMAN;ADH1B_HUMAN;ADH4_HUMAN;ADH6_HUMAN;CP1A2_HUMAN;GSTA1_HUMAN;UDB17_HUMAN
Tissue		
Liver	5.51e-04	ACBP_HUMAN;ADH1A_HUMAN;ADH1B_HUMAN;ADH4_HUMAN;ADH6_HUMAN;ALBU_HUMAN;ASSY_HUMAN;CLC4M_HUMAN;CO1A2_HUMAN;CP1A2_HUMAN;CRP_HUMAN;CYB5_HUMAN;GSTA1_HUMAN;SAA1_HUMAN;UDB17_HUMAN
Erythrocyte	2.95e-3	ALBU_HUMAN;CRP_HUMAN;CYB5_HUMAN
Venous blood	4.25e-3	ALBU_HUMAN;CRP_HUMAN
Disease		
Alcohol dependence	1.33e-2	ADH1B_HUMAN;ADH4_HUMAN
Alcohol use disorder	2.06e-2	ADH1B_HUMAN;ADH4_HUMAN
Hepatobiliary disease	4.05e-2	ALBU_HUMAN;CRP_HUMAN;SAA1_HUMAN

Confusion Matrix for Liver 3-Way Unmatched Balanced Proteomic dataset:

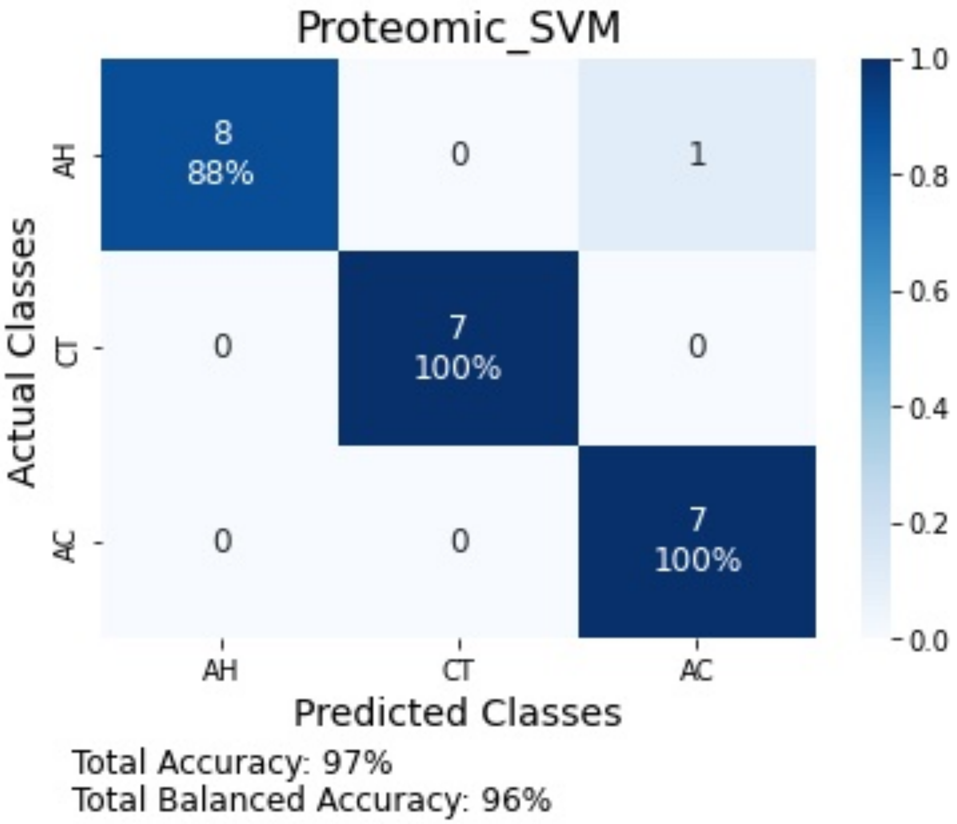

Figure S in S1 Text: Confusion matrix for nested cross-validated classification of Liver 3-Way Unmatched Balanced proteomic dataset.

Confusion Matrix in Independent Validation Data of Liver 3-Way Unmatched Balanced Proteomic dataset:

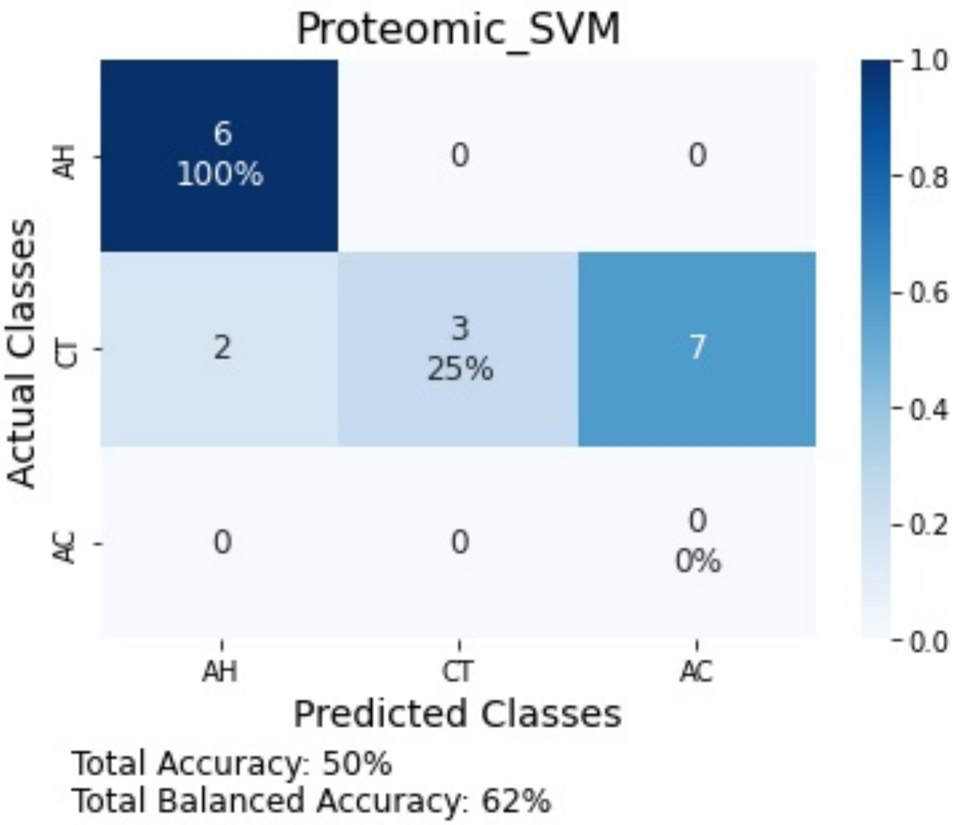

Figure T in S1 Text: Confusion matrix for classification of Liver 3-Way Unmatched Balanced proteomic dataset in independent validation data.

Heatmaps for Liver 3-Way Unmatched Balanced Proteomic dataset:

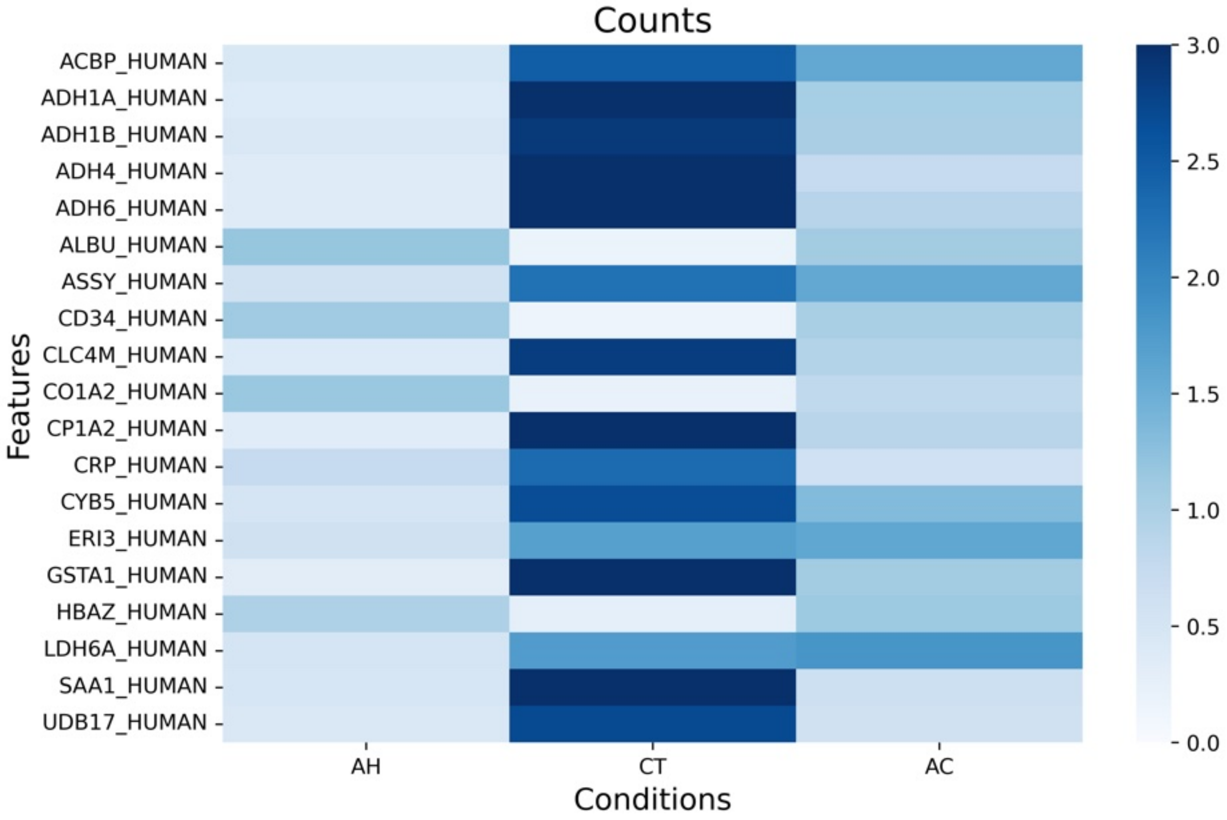

Figure U in S1 Text: Heatmap of proteomic counts for Liver 3-Way Unmatched Balanced dataset averaged per condition.

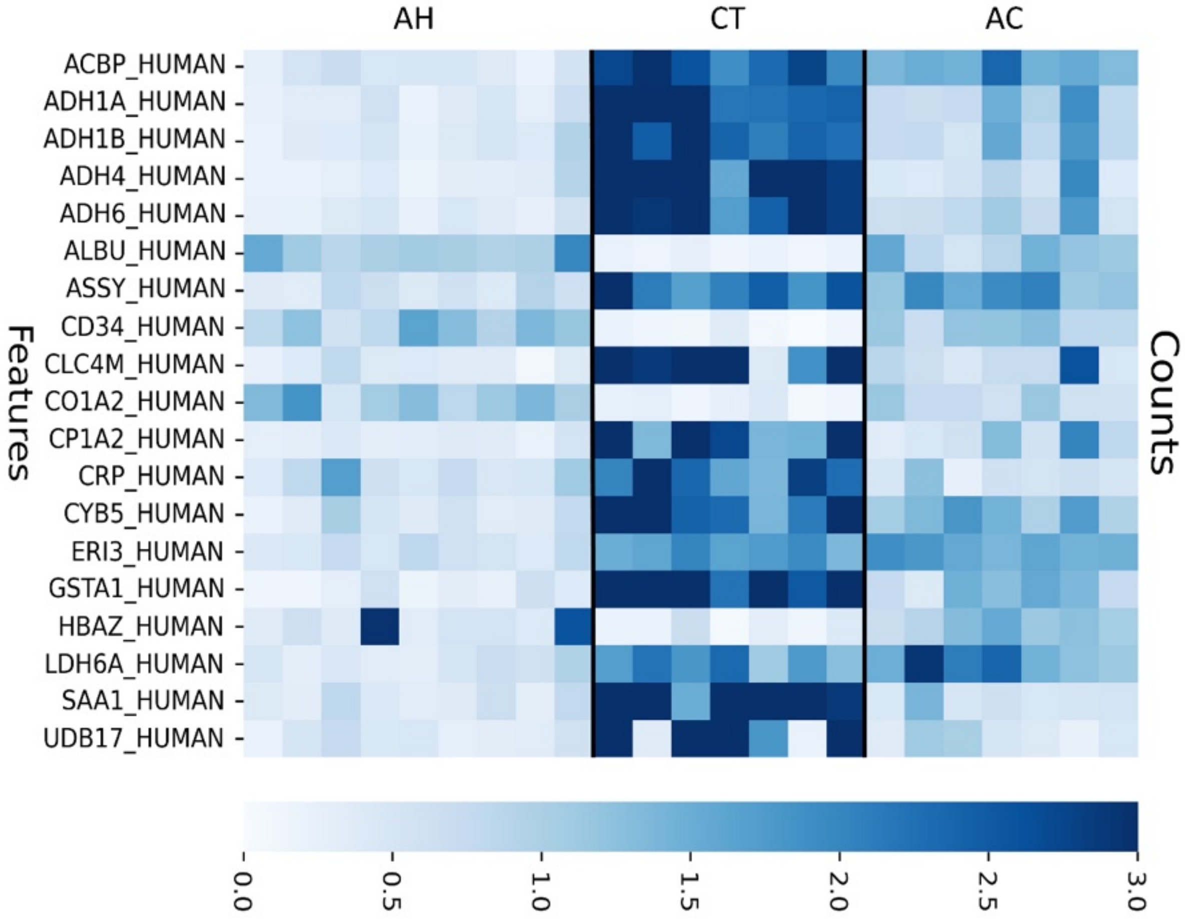

Figure V in S1 Text: Heatmap of proteomic counts for Liver 3-Way Unmatched Balanced dataset.

Heatmaps in Liver 3-Way Unmatched Balanced Proteomic external validation dataset:

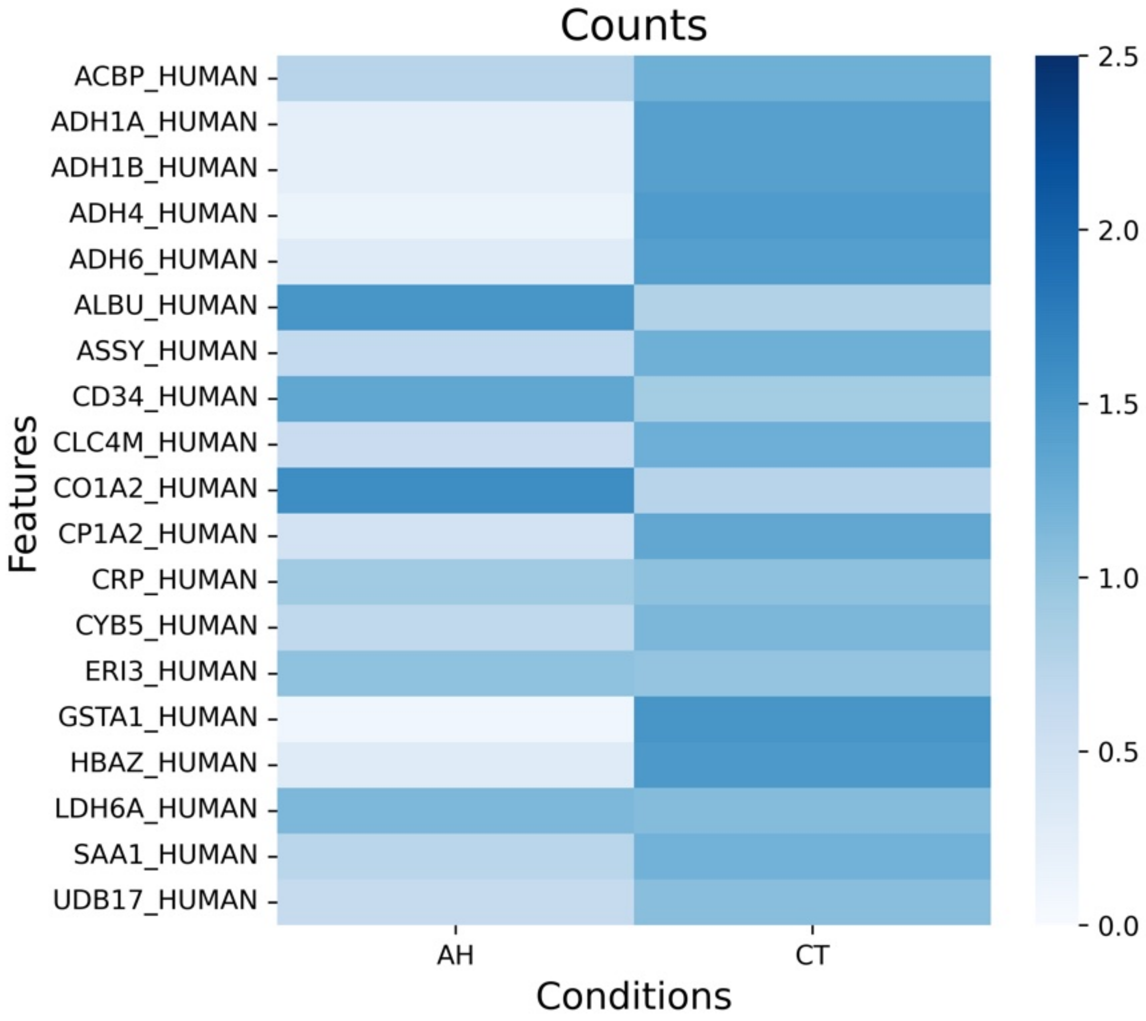

Figure W in S1 Text: Heatmap of proteomic counts for independent liver validation dataset averaged per condition.

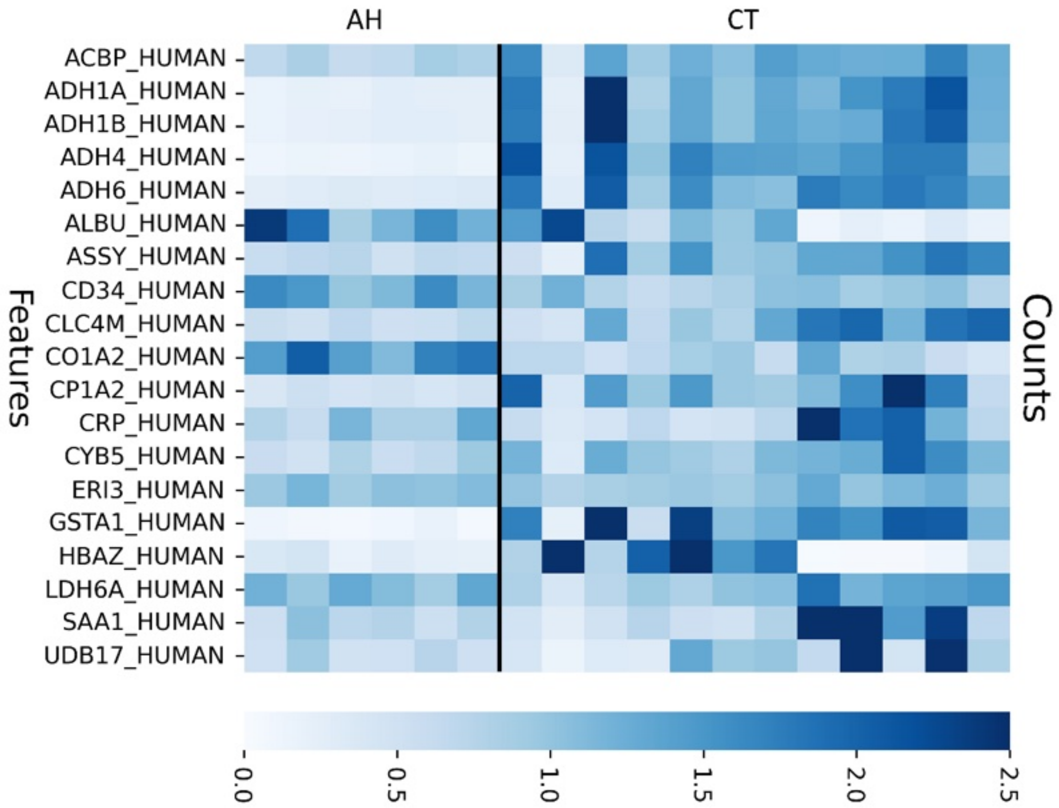

Figure X in S1 Text: Heatmap of proteomic counts for independent liver validation dataset.

d. PBMC 3-Way Unmatched Balanced (AH vs Healthy vs AC)

i. TRANSCRIPTOMIC SECTION for PBMC 3-Way Unmatched Balanced dataset:

Classification Performance:

SVM / Union:

Table U in S1 Text: The classification performances of support vector machine model across a range of feature sizes (10-500) and feature selection techniques (differential expression and information gain with standard deviation thresholds of 2.5, 3.0, 3.5). The accuracy corresponding to best gene set is highlighted in green.

Feature Size	DE 2.5	DE 3	DE 3.5	IG 2.5	IG 3	IG 3.5
10	0.72	0.70	0.65	0.76	0.73	0.70
25	0.69	0.66	0.70	0.76	0.74	0.74
50	0.77	0.69	0.72	0.76	0.74	0.72
100	0.72	0.67	0.77	0.77	0.77	0.76
150	0.72	0.69	0.70	0.74	0.74	0.76
200	0.72	0.68	0.73	0.78	0.75	0.77
250	0.69	0.65	0.78	0.73	0.71	0.76
300	0.72	0.74	0.72	0.77	0.69	0.73
350	0.68	0.71	0.69	0.77	0.68	0.73
400	0.70	0.69	0.67	0.77	0.74	0.74
450	0.70	0.67	0.67	0.74	0.76	0.74
500	0.71	0.69	0.64	0.78	0.74	0.76

Enrichr In Silico Biological Validation:

Union / 4 out of 5 Merge:

Table V in S1 Text: The Enrichr hits Union / 4 out of 5 Merge configuration in PBMC 3-Way Unmatched Balanced dataset.

Feature Size	DE 2.5	DE 3	DE 3.5	IG 2.5	IG 3	IG 3.5
10 – 3/7/7/1/2/2	28/2/2	18/4/6	19/2/4	4/1/5	5/3/4	5/3/4
25 – 11/17/14/4/5/5	35/2/13	28/6/6	18/5/5	23/0/1	13/0/0	13/0/0
50 – 21/38/39/12/14/14	45/7/9	32/7/15	24/8/15	5/1/2	39/4/1	0/4/1
100 – 41/64/72/21/34/32	52/8/18	37/12/24	41/11/20	0/0/1	7/6/3	7/6/2
150 – 69/107/111/37/54/51	65/7/14	74/12/24	65/12/22	1/3/1	5/8/2	8/9/2
200 – 108/146/157/58/74/74	42/13/16	95/14/25	87/12/30	1/4/4	11/10/3	5/9/3
250 – 130/171/193/78/89/96	58/13/20	84/13/28	113/14/28	21/4/5	15/10/4	7/10/3
300 – 155/213/227/94/118/116	79/14/16	110/15/28	92/14/29	11/4/5	11/9/5	6/9/6
350 – 186/251/267/109/144/149	71/12/18	105/15/31	107/15/28	11/4/5	13/11/6	10/11/8
400 – 201/289/305/126/169/176	100/11/20	122/15/33	106/15/31	20/4/4	26/12/8	16/12/10
450 – 218/324/347/147/203/205	93/11/21	140/16/30	134/15/36	22/4/4	28/12/11	14/12/12
500 – 235/364/393/169/225/229	99/11/25	159/16/38	139/15/34	12/5/4	31/11/12	17/10/9

Best gene set for PBMC 3-Way Unmatched Balanced dataset: ADM, ALAS2, C1QA, DYSF, ELANE, FAM20A, FCGR1A, HBD, HP, IGHV1-69, IGHV4-4, IGKV1D-13, LCN2, MEFV, MMP9, MSR1, PGLYRP1, RETN, RNASE2, S100P, VSIG4 – 21 genes.

Table W in S1 Text: Top Enrichr hits for PBMC 3-Way Unmatched Balanced dataset (RNAseq).

Term	Adjusted P-Value	Genes
**Pathway**		
phagocytosis	2.33e-05	MSR1;IGHV4-4;IGHV1-69;FCGR1A;ELANE
neutrophil degranulation	2.33e-05	LCN2;S100P;RETN;RNASE2;PGLYRP1;MMP9;ELANE
phagocytosis, engulfment	1.46e-04	MSR1;IGHV4-4;IGHV1-69;FCGR1A
**Tissue**		
PERIPHERAL BLOOD	1.54e-07	ALAS2;IGHV4-4;DYSF;HBD;IGHV1-69;RETN;MEFV;RNASE2;MMP9;IGKV1D-13;LCN2;S100P;FCGR1A;PGLYRP1
GRANULOCYTE	1.57e-05	DYSF;LCN2;ADM;S100P;VSIG4;FAM20A;MEFV;RNASE2;FCGR1A;PGLYRP1;MMP9;ELANE
NEUTROPHIL	1.11e-04	DYSF;LCN2;ADM;S100P;RETN;MEFV;RNASE2;FCGR1A;PGLYRP1;MMP9;ELANE
**Disease**		
Septic Shock human	2.76e-12	C1QA;HP;DYSF;LCN2;ADM;S100P;RETN;VSIG4;FAM20A;RNASE2;PGLYRP1;MMP9
familial combined hyperlipidemia human	6.17e-05	DYSF;LCN2;ADM;S100P;MEFV;RNASE2
hepatitis C virus related hepatocellular carcinoma human	1.17e-03	DYSF;LCN2;S100P;HBD;MMP9

Confusion matrix for PBMC 3-Way Unmatched Balanced RNAseq dataset:

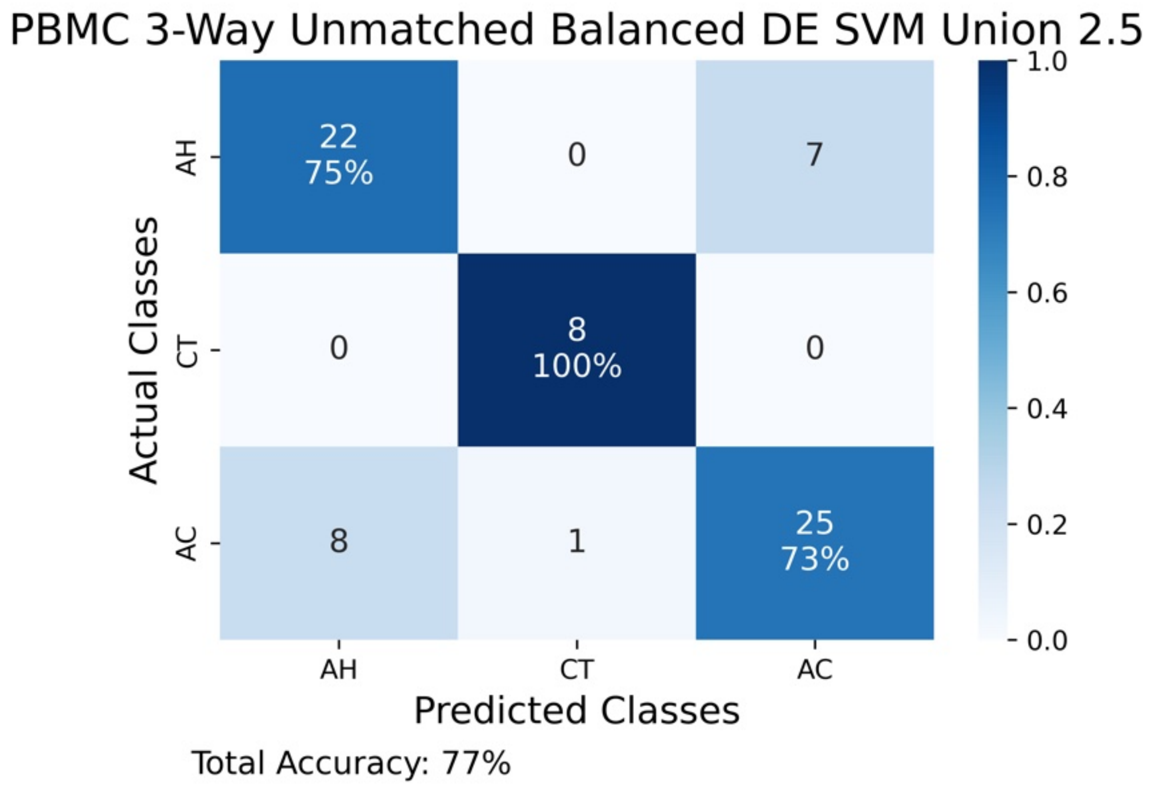

Figure Y in S1 Text: Confusion matrix for nested cross-validated classification of PBMC 3-Way Unmatched Balanced RNAseq dataset.

Heatmaps for PBMC 3-Way Unmatched Balanced RNAseq dataset:

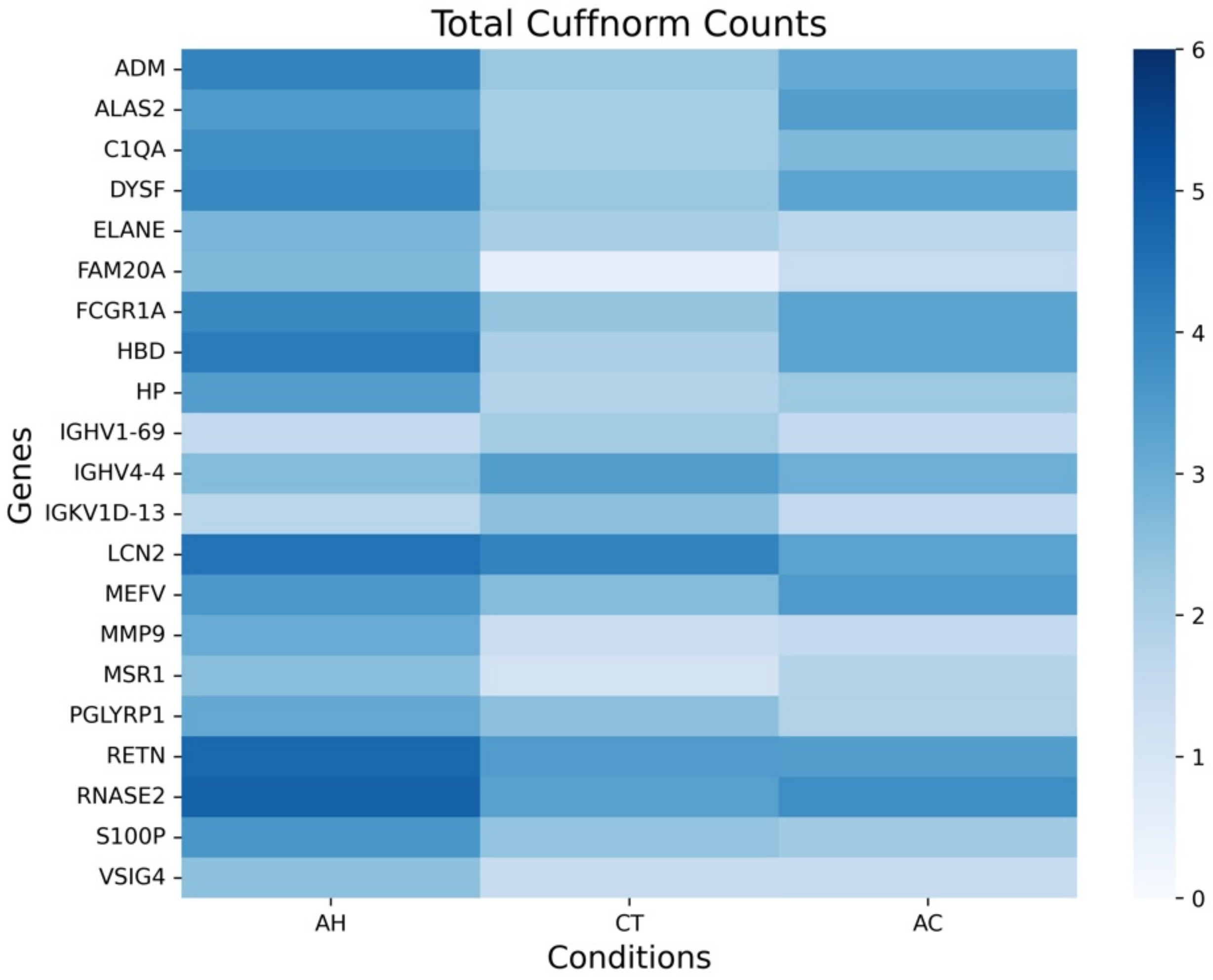

Figure Z in S1 Text: Heatmap of RNAseq counts for PBMC 3-Way Unmatched Balanced dataset averaged per condition.

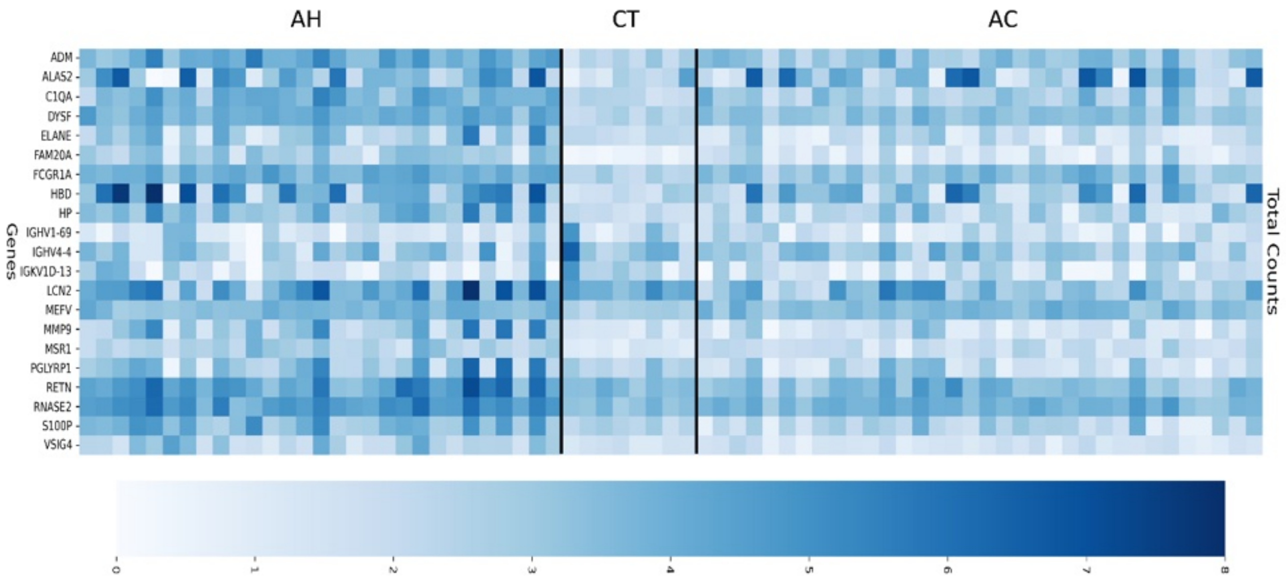

Figure AA in S1 Text: Heatmap of RNAseq counts for PBMC 3-Way Unmatched Balanced dataset.

ii. PROTEOMIC SECTION for PBMC 3-Way Unmatched Balanced dataset:

Classification Performance:

Logistic Regression:

DE Variance (2.5):

Table X in S1 Text: The classification performances of logistic regression model across a range of feature sizes (15-200) and imputation techniques (median and zero based methods with imputation thresholds of 0, 0.05, 0.1). The features were selected using differential expression and variance 2.5 filter.

	Median 0	Median 0.05	Median 0.1	Zero 0	Zero 0.05	Zero 0.1
15	0.72	0.72	0.72	0.72	0.72	0.72
25	0.76	0.76	0.76	0.76	0.76	0.76
35	0.74	0.74	0.74	0.74	0.74	0.74
50	0.74	0.74	0.74	0.74	0.74	0.74
60	0.82	0.82	0.82	0.82	0.82	0.82
70	0.86	0.86	0.86	0.86	0.86	0.86
80	0.86	0.86	0.86	0.86	0.86	0.86
90	0.89	0.89	0.85	0.89	0.89	0.89
100	0.93	0.93	0.93	0.93	0.93	0.93
150	0.89	0.89	0.89	0.89	0.89	0.89
200	0.93	0.93	0.93	0.93	0.93	0.93

Biological Validation (in-silico):

FS Test = 5:

DE Variance (2.5):

Table Y in S1 Text: The AGOTOOL hits 5 out of 5 Merge configuration in PBMC 3-Way Unmatched Balanced dataset. The features were selected using differential expression and variance 2.5 filter.

	0	0.05	0.1
15 - 5/5/5	10/3/0	10/3/0	10/3/0
25 - 9/9/9	13/3/0	13/3/0	13/3/0
35 - 17/17/17	15/4/0	15/4/0	15/4/0
50 - 23/23/23	16/5/0	16/5/0	16/5/0
60 - 29/29/29	16/5/0	16/5/0	16/5/0
70 - 33/33/33	17/7/0	17/7/0	17/7/0
80 - 39/39/39	20/6/0	20/6/0	20/6/0
90 - 48/48/48	23/6/0	23/6/0	23/6/0
100 - 53/53/53	21/6/0	21/6/0	21/6/0
150 - 71/71/71	22/7/0	22/7/0	22/7/0
200 - 103/103/102	21/8/0	21/8/0	21/8/0

Best protein set for PBMC 3-Way Unmatched Balanced dataset: ACTN1_HUMAN, ALBU_HUMAN, CCL5_HUMAN, CXCL7_HUMAN, FHL1_HUMAN, FIBA_HUMAN, FIBB_HUMAN, FIBG_HUMAN, FRIL_HUMAN, FSTL1_HUMAN, GP1BB_HUMAN, ILK_HUMAN, ITA2B_HUMAN, ITB1_HUMAN, ITB3_HUMAN, LIMS1_HUMAN, LYSC_HUMAN, MYL9_HUMAN, PP14A_HUMAN, RAP1A_HUMAN, RS4Y1_HUMAN, SBP1_HUMAN, SDPR_HUMAN, TBA4A_HUMAN, TBA8_HUMAN, TBB1_HUMAN, TPM2_HUMAN, TRML1_HUMAN, TSN15_HUMAN, TSP1_HUMAN, URP2_HUMAN, VINC_HUMAN, VTDB_HUMAN – 33 proteins.

Top AGOTOOL Hits:

Table Z in S1 Text: Top AGOTOOL hits for PBMC 3-Way Unmatched Balanced dataset (RNAseq).

Pathway		
Term	Adjusted P-Value	Proteins
Platelet activation	2.34e-05	FIBA_HUMAN;FIBB_HUMAN;FIBG_HUMAN;GP1BB_HUMAN;ITA2B_HUMAN;ITB1_HUMAN;ITB3_HUMAN;RAP1A_HUMAN;URP2_HUMAN
Complement system	2.89e-04	ALBU_HUMAN;FIBA_HUMAN;FIBB_HUMAN;FIBG_HUMAN;ITA2B_HUMAN;ITB3_HUMAN;TSP1_HUMAN
COVID-19, thrombosis and anticoagulation	2.89e-04	FIBA_HUMAN;FIBB_HUMAN;FIBG_HUMAN
Tissue		
Blood platelet	2.67e-04	ACTN1_HUMAN;ALBU_HUMAN;CXCL7_HUMAN;FIBA_HUMAN;FIBB_HUMAN;FIBG_HUMAN;GP1BB_HUMAN;ITA2B_HUMAN;ITB3_HUMAN;LIMS1_HUMAN;PP14A_HUMAN;RAP1A_HUMAN;TBA4A_HUMAN;TBB1_HUMAN;TRML1_HUMAN;TSP1_HUMAN;URP2_HUMAN;VINC_HUMAN
Blood	2.91e-04	ACTN1_HUMAN;ALBU_HUMAN;CCL5_HUMAN;CXCL7_HUMAN;FHL1_HUMAN;FIBA_HUMAN;FIBB_HUMAN;FIBG_HUMAN;FRIL_HUMAN;GP1BB_HUMAN;ITA2B_HUMAN;ITB3_HUMAN;LIMS1_HUMAN;LYSC_HUMAN;PP14A_HUMAN;RAP1A_HUMAN;TBA4A_HUMAN;TBB1_HUMAN;TRML1_HUMAN;TSP1_HUMAN;URP2_HUMAN;VINC_HUMAN
Blood plasma	2.91e-04	ACTN1_HUMAN;ALBU_HUMAN;CXCL7_HUMAN;FHL1_HUMAN;FIBA_HUMAN;FIBB_HUMAN;FIBG_HUMAN;GP1BB_HUMAN;ITA2B_HUMAN;ITB3_HUMAN;LIMS1_HUMAN;PP14A_HUMAN;RAP1A_HUMAN;TBA4A_HUMAN;TBB1_HUMAN;TRML1_HUMAN;TSP1_HUMAN;URP2_HUMAN;VINC_HUMAN
Disease		
NA	NA	NA
NA	NA	NA
NA	NA	NA

Confusion Matrix for PBMC 3-Way Unmatched Balanced Proteomics dataset:

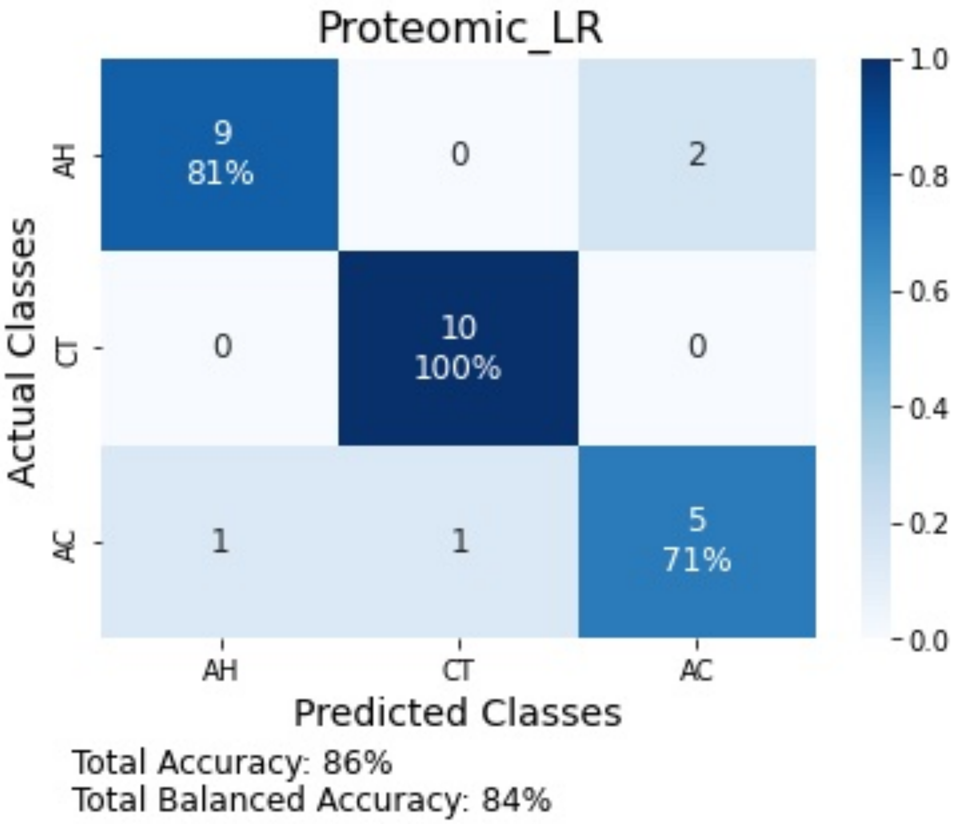

Figure AB in S1 Text: Confusion matrix for nested cross-validated classification of PBMC 3-Way Unmatched Balanced proteomic dataset.

Heatmaps for PBMC 3-Way Unmatched Balanced Proteomic dataset:

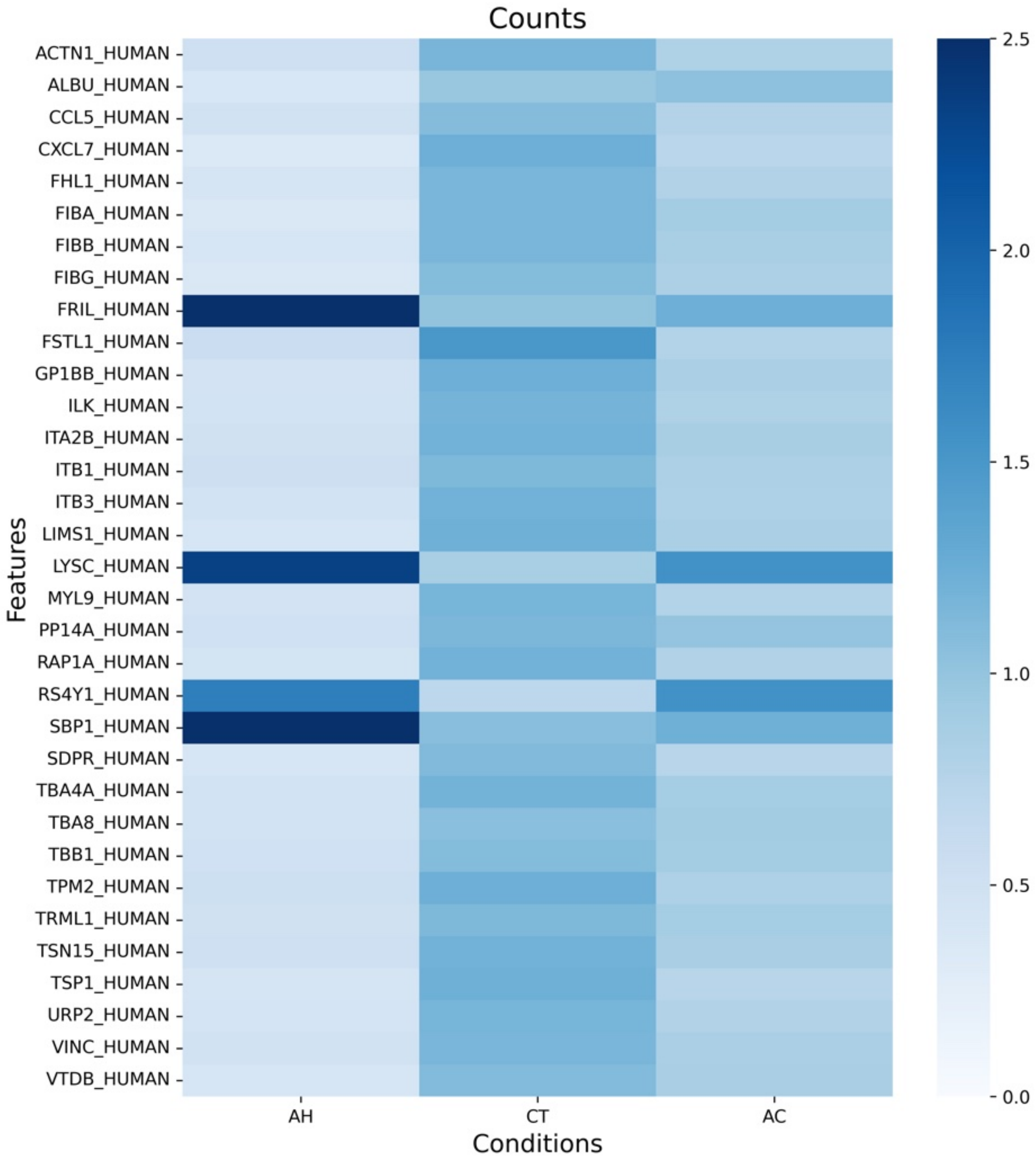

Figure AC in S1 Text: Heatmap of proteomic counts for PBMC 3-Way Unmatched Balanced dataset averaged per condition.

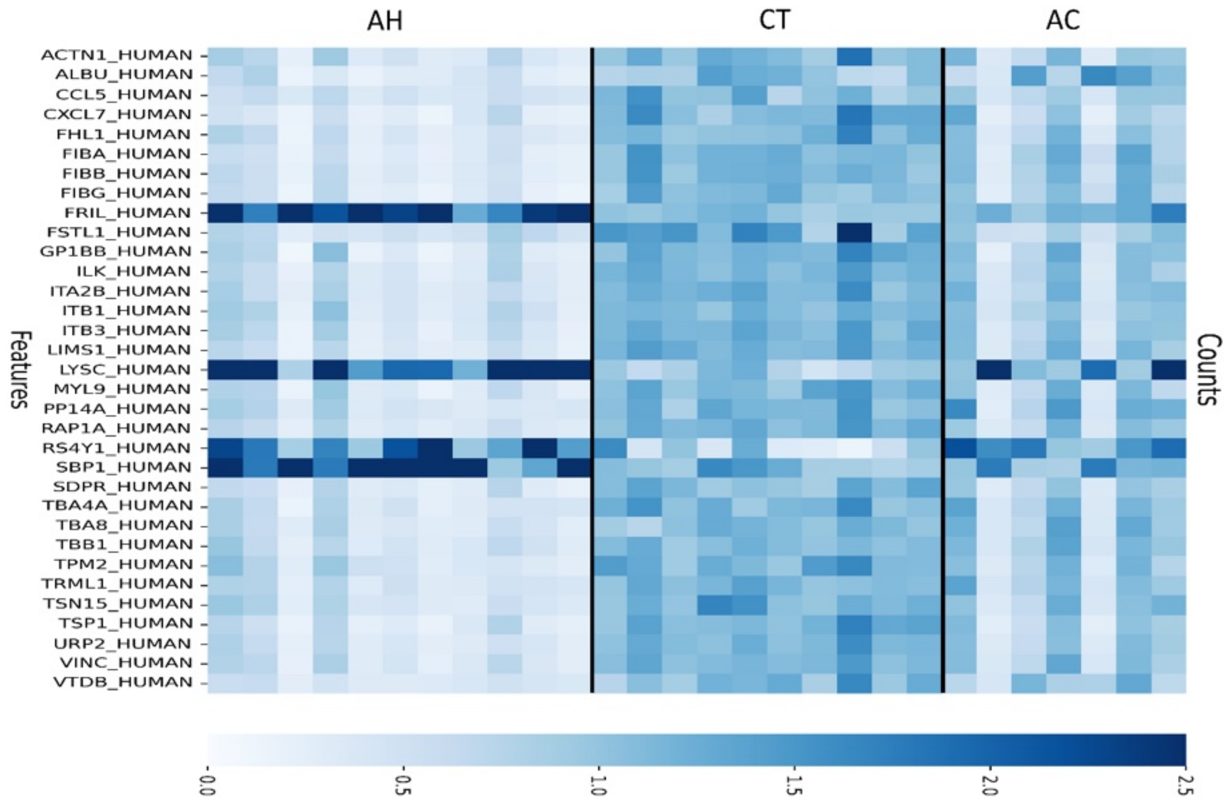

Figure AD in S1 Text: Heatmap of proteomic counts for PBMC 3-Way Unmatched Balanced dataset.

e. Integrated Analysis of LV 3-Way (AH vs Healthy vs AC)

For integrated analysis section we will be using logistic regression and linear kernel SVM models only for speed of execution and convenience of interpretation (allows for model-based feature ranking). The LR configurations are picked based on their performance in unmatched balanced datasets. These configurations are also executed with linear SVMs based on the assumption that LR and linear SVM are similar.

Table AA in S1 Text: Some of the best performing transcriptomic and proteomic configurations from Liver 3-Way Unmatched Balanced datasets were paired together for integrated analysis. The best performing pair is highlighted in green. Columns are proteomic pipeline configurations, rows are transcriptomic pipeline configurations.

	DE None 35 LR FS Test 3 (19) (13/4/3)	DE None 50 LR FS Test 4 (24) (15/3/2)	DE None 35 SVM FS Test 3 (19) (13/4/3)	DE None 50 SVM FS Test 4 (24) (15/3/2)
Union 100 DE 3.5 LR FS Test 5. (31) (25/8/29)	0.93 (RNA: 0.13, Prot: 1.0)	0.96 (RNA: 0.13, Prot: 1.0)	0.83	0.83
Union 150 DE 3.5 LR FS Test 5. (45) (27/6/24)	0.9	0.96 (RNA: 0.2, Prot: 1.0)	0.9	0.9
Union 200 DE 3.5 LR FS Test 5. (59) (28/6/28)	0.96 (RNA: 0.83, Prot: 1.0)	0.96 (RNA: 0.83, Prot: 1.0)	0.96 (RNA: 0.83, Prot: 1.0)	0.96 (RNA: 0.83, Prot: 1.0)
Union 100 DE 3.5 SVM FS Test 5. (31) (25/8/29)	0.9	0.96 (RNA: 0.2 ,Prot: 1.0)	0.9	0.9
Union 150 DE 3.5 SVM FS Test 5. (45) (27/6/24)	0.9	0.93	0.9	0.9
Union 200 DE 3.5 SVM FS Test 5. (59) (28/6/28)	0.93	0.96	0.9	0.9

ROC Curve of Best Integrated Protein and Gene Set for Liver Tissue:

The receiver operating characteristic curves were generated using one-vs-rest micro-averaging scheme as described in scikit-learn package: scikit-learn.org/stable/auto_examples/model_selection/plot_roc. The classes were not weighted by sample size.

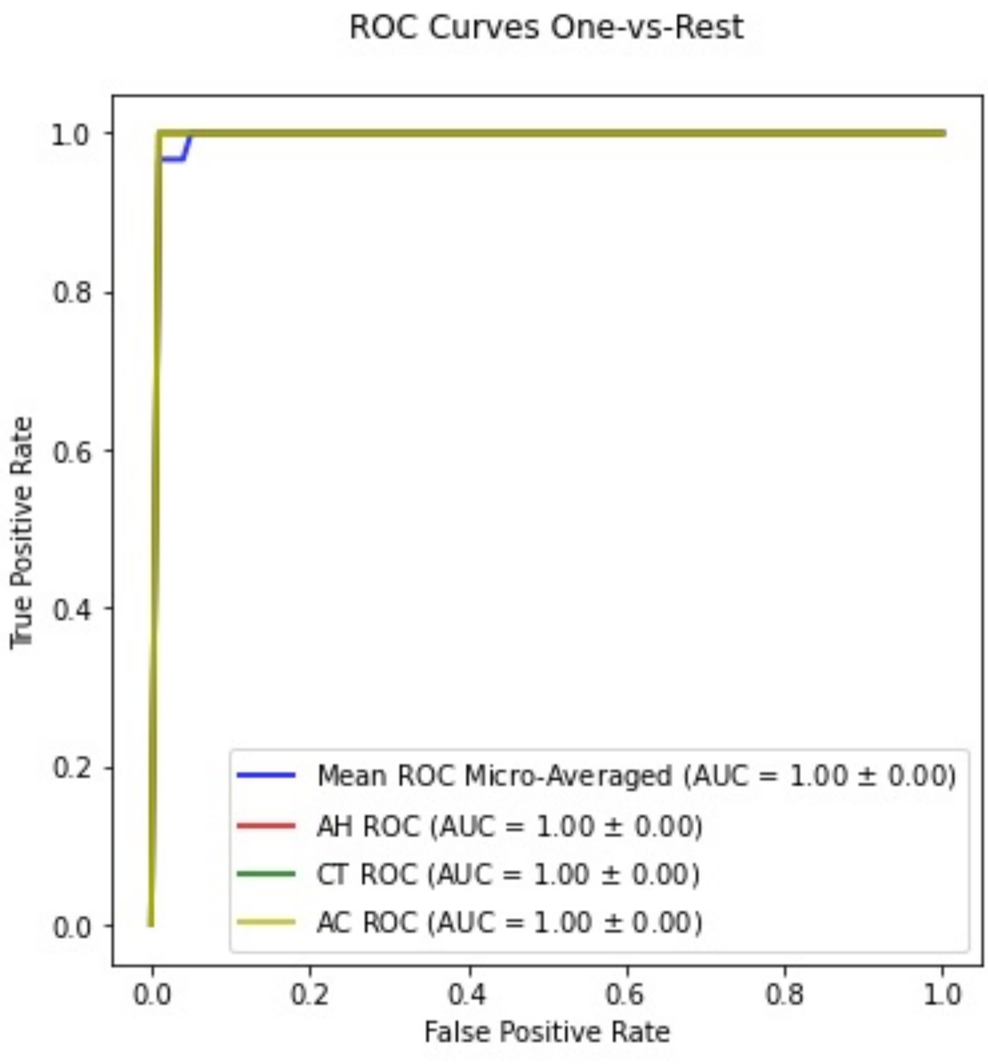

Figure AE in S1 Text: The receiver operating characteristic curve of the best integrated gene-protein Liver 3-Way model.

Per-class Sensitivities and Specificities of Best Integrated Protein and Gene Set:

AH sensitivity = 1

AH specificity = 0.83

CT sensitivity = 1

CT specificity = 1

AC sensitivity = 0.66

AC specificity = 1

ROC Curve of Best Gene Set for Liver Tissue:

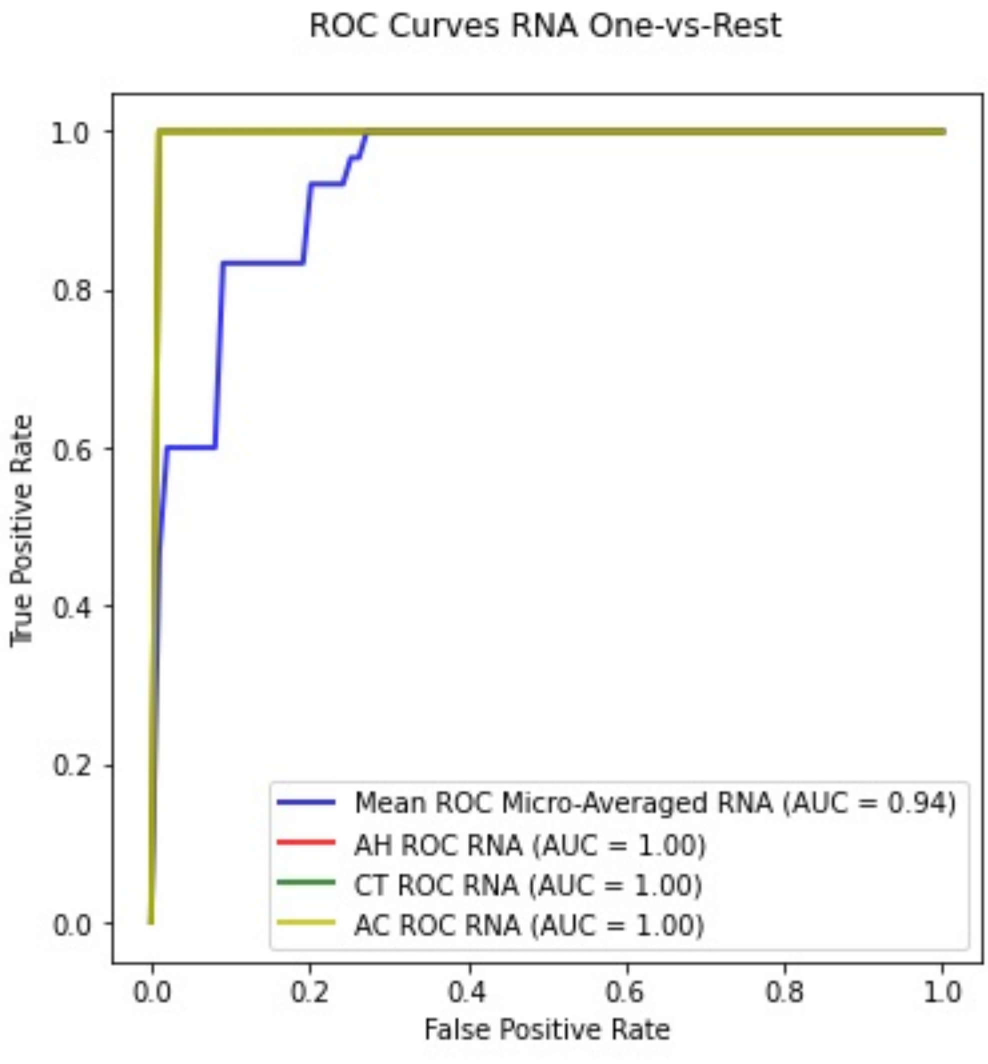

Figure AF in S1 Text: The receiver operating characteristic curve of the best transcriptomic Liver 3-Way model.

Per-class Sensitivities and Specificities of Best Gene Set:

AH sensitivity = 0.79

AH specificity = 1

CT sensitivity = 1

CT specificity = 1

AC sensitivity = 1

AC specificity = 0.81

ROC Curve of Best Protein Set for Liver Tissue:

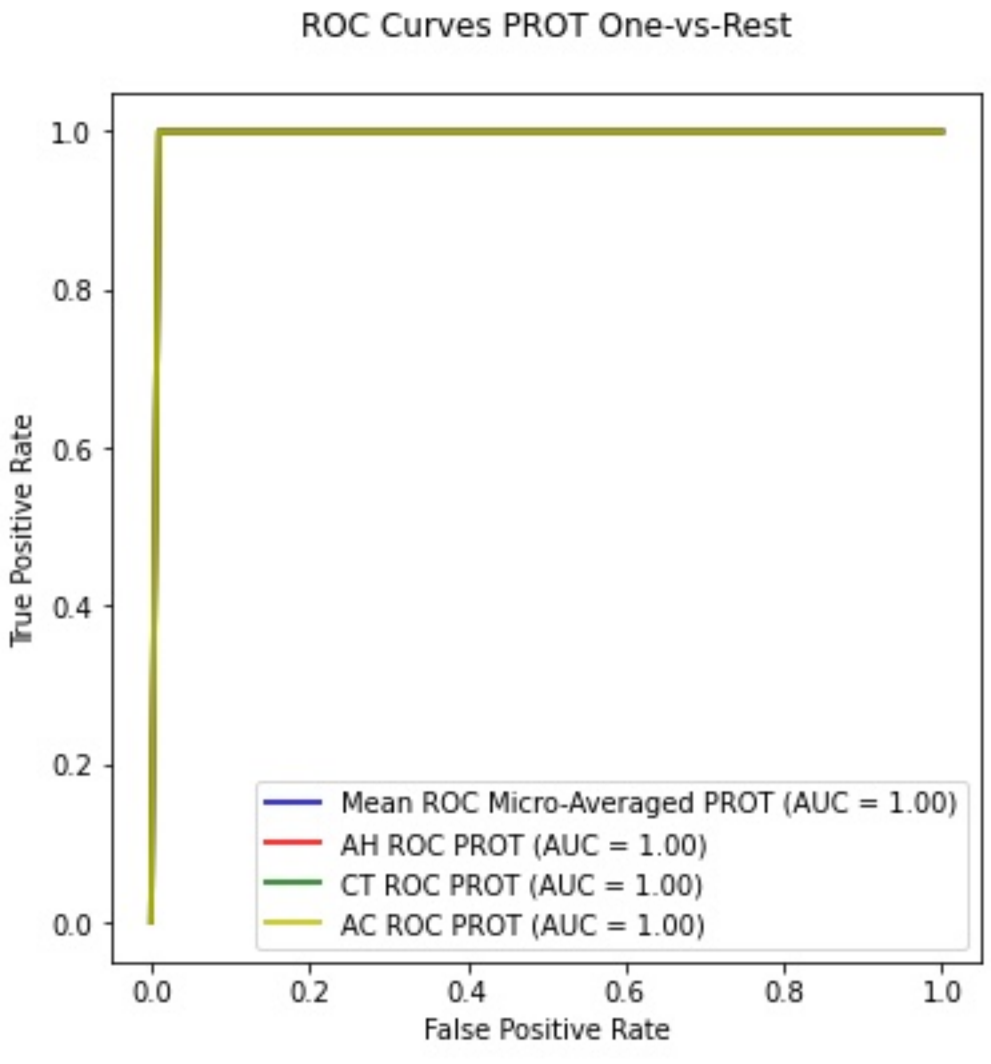

Figure AG in S1 Text: The receiver operating characteristic curve of the best proteomic Liver 3-Way model.

Per-class Sensitivities and Specificities of Best Protein Set:

AH sensitivity = 1

AH specificity = 1

CT sensitivity = 1

CT specificity = 1

AC sensitivity = 1

AC specificity = 1

Best protein set for Integrated Liver 3-Way analysis:

ACBP_HUMAN, ADH1A_HUMAN, ADH1B_HUMAN, ADH4_HUMAN, ADH6_HUMAN, ALBU_HUMAN, ASSY_HUMAN, CD34_HUMAN, CLC4M_HUMAN, CO1A2_HUMAN, CP1A2_HUMAN, CRP_HUMAN, CYB5_HUMAN, ERI3_HUMAN, GSTA1_HUMAN, HBAZ_HUMAN, LDH6A_HUMAN, SAA1_HUMAN, UDB17_HUMAN – 19 proteins.

Splitting best proteins by their differential expression status in each pairwise comparison of corresponding unmatched balanced dataset:

AH vs CT: ACBP, ADH1A, ADH1B, ADH4, ADH6, ALBU, ASSY, CD34, CLC4M, CO1A2, CP1A2, CRP, CYB5, ERI3, GSTA1, LDH6A, SAA1, UDB17.

AH vs AC: ACBP, ADH1A, ADH1B, ADH6, ASSY, CYB5, ERI3, GSTA1, LDH6A.

CT vs AC: ACBP, ADH1A, ADH1B, ADH4, ADH6, ALBU, CD34, CLC4M, CO1A2, CRP, CYB5, GSTA1, HBAZ, SAA1.

Table AB in S1 Text: Top 20 AGOTOOL hits per each pairwise comparison of Liver 3-Way Integrated dataset. The pathways were sorted by adjusted p-value.

AH vs CT	AH vs AC	CT vs AC
tyrosine metabolism	glycolysis / gluconeogenesis	tyrosine metabolism
fatty acid degradation	drug metabolism - cytochrome P450	fatty acid degradation
drug metabolism - cytochrome P450	metabolism of xenobiotics by cytochrome P450	retinol metabolism
metabolism of xenobiotics by cytochrome P450	chemical carcinogenesis	drug metabolism - cytochrome P450
retinol metabolism	tyrosine metabolism	metabolism of xenobiotics by cytochrome P450
glycolysis / gluconeogenesis	fatty acid degradation	glycolysis / Gluconeogenesis
chemical carcinogenesis	fatty acid omega-oxidation	chemical carcinogenesis
ethanol metabolic process	retinol metabolism	retinol metabolic process
terpenoid metabolic process	ethanol oxidation	olefinic compound metabolic process
retinol metabolic process	carboxylic acid metabolic process	fatty acid omega-oxidation
metabolic pathways	olefinic compound metabolic process	ethanol oxidation

olefinic compound metabolic process	retinoic acid metabolic process	monocarboxylic acid metabolic process
monocarboxylic acid metabolic process	retinol metabolic process	retinoic acid metabolic process
ethanol oxidation	monocarboxylic acid metabolic process	vitamin B12 metabolism
carboxylic acid metabolic process	metabolic pathways	folate metabolism
hormone metabolic process	cellular lipid metabolic process	selenium micronutrient network
fatty acid omega-oxidation		complement system
lipid metabolic process		cellular lipid metabolic process
retinoic acid metabolic process		
cellular lipid metabolic process		

Protein ranking by model coefficients for Liver 3-Way Integrated dataset:

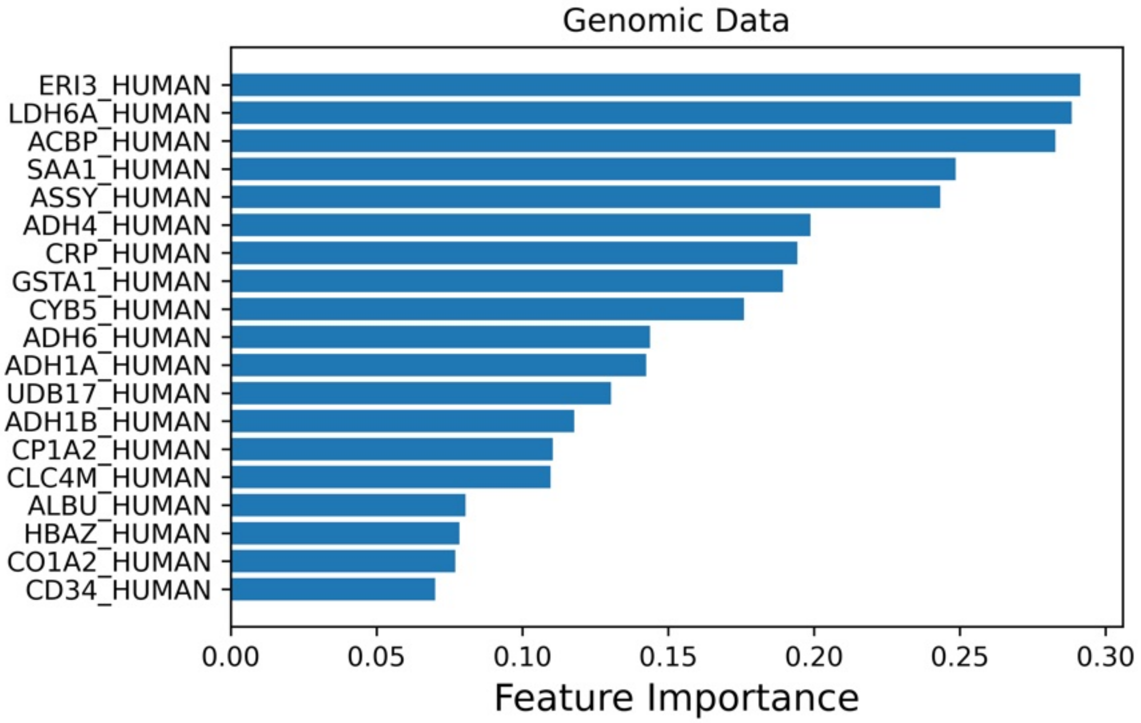

Figure AH in S1 Text: Top ranked proteins for integrated analysis of Liver 3-Way.

Best gene set for Integrated Liver 3-Way Analysis:ACKR1, AKR1B10, BBOX1, C15orf52, CFTR, CLEC4M, CREB3L3, CSF3R, CXCL1, CXCL6, DCDC2, DHODH, DHRS2, F3, FABP4, FAM118A, FCGR3B, FCN3, GADD45B, GADD45G, GPC3, GSTA2, HAMP, HAO2, ID4, IGSF9, IL7R, KRT23, LBP, LCN2, LRG1, MARCO, MMP7, MT1A, MT1G, MT1H, MT1M, MT1X, MUC13, MUC6, NRTN, PAPLN, PID1, PLA2G2A, PLCB1, PPP1R1A, S100A12, S100A8, S100A9, SLC13A5, SLC22A1, SOCS1, SPINK1, STAG3, STMN2, TREM2, TRIB3, VSIG2, VTCN1 – 59 genes.

Splitting best genes by their differential expression status in each pairwise comparison of corresponding unmatched balanced dataset:

AH vs CT: ACKR1, AKR1B10, BBOX1, C15orf52, CFTR, CLEC4M, CREB3L3, CSF3R, CXCL1, CXCL6, DCDC2, DHODH, DHRS2, FABP4, FAM118A, FCGR3B, FCN3, GADD45B, GADD45G, GPC3, GSTA2, HAMP, HAO2, IGSF9, KRT23, LBP, LRG1, MARCO, MMP7, MT1A, MT1G, MT1H, MT1M, MT1X, MUC13, MUC6, NRTN, PAPLN, PID1, PLA2G2A, PLCB1, PPP1R1A, SLC13A5, SLC22A1, SOCS1, SPINK1, STAG3, STMN2, TREM2, TRIB3, VSIG2, VTCN1.

AH vs AC: AKR1B10, BBOX1, CSF3R, CXCL1, CXCL6, F3, FAM118A, FCGR3B, FCN3, GADD45B, GADD45G, GSTA2, HAMP, ID4, IL7R, LCN2, MUC13, S100A12, S100A8, S100A9, SPINK1, TREM2.

CT vs AC: ACKR1, AKR1B10, BBOX1, C15orf52, CFTR, CLEC4M, CREB3L3, CXCL6, DCDC2, DHODH, DHRS2, F3, FABP4, FAM118A, FCN3, GPC3, HAMP, HAO2, ID4, IGSF9, IL7R, KRT23, LBP, LCN2, LRG1, MARCO, MMP7, MT1A, MT1G, MT1H, MT1M, MT1X, MUC13, MUC6, NRTN, PAPLN, PID1, PLA2G2A, PLCB1, PPP1R1A, S100A12, S100A8, S100A9, SLC13A5, SLC22A1, SOCS1, SPINK1, STAG3, STMN2, TRIB3, VSIG2, VTCN1.

Table AC in S1 Text: Top 20 Enrichr hits per each pairwise comparison of Liver 3-Way Integrated dataset. The pathways were sorted by adjusted p-value.

AH vs CT	AH vs AC	CT vs AC
cellular response to zinc ion	oncostatin M	cellular zinc ion homeostasis
cellular response to copper ion	IL-17 signaling pathway	oncostatin M
response to copper ion	neutrophil chemotaxis	cellular response to zinc ion
cellular response to cadmium ion	granulocyte chemotaxis	cellular response to copper ion
cellular zinc ion homeostasis	neutrophil migration	cellular transition metal ion homeostasis
zinc ion homeostasis	neutrophil mediated immunity	response to copper ion
response to cadmium ion	defense response to fungus	cellular response to cadmium ion
response to zinc ion	neutrophil degranulation	zinc ion homeostasis
zinc homeostasis	neutrophil activation involved in immune response	response to cadmium ion
cellular transition metal ion homeostasis	antimicrobial humoral immune response mediated by antimicrobial peptide	response to zinc ion
mineral absorption	leukocyte aggregation	positive regulation of inflammatory response
cellular response to metal ion	activation of MAPKKK activity	zinc homeostasis
cellular divalent inorganic cation homeostasis	cytokine-mediated signaling pathway	mineral absorption
oncostatin M	positive regulation of inflammatory response	cellular response to metal ion
copper homeostasis	positive regulation of defense response	cellular divalent inorganic cation homeostasis
negative regulation of growth	TGF-beta regulation of extracellular matrix	copper homeostasis
activation of MAPKKK activity	defense response to bacterium	neutrophil chemotaxis
epithelial structure maintenance	positive regulation of response to external stimulus	defense response to fungus
maintenance of gastrointestinal epithelium	positive regulation of intracellular signal transduction	granulocyte chemotaxis
regulation of fertilization	inflammatory response	negative regulation of growth

Gene ranking by model coefficients for Liver 3-Way Integrated dataset:

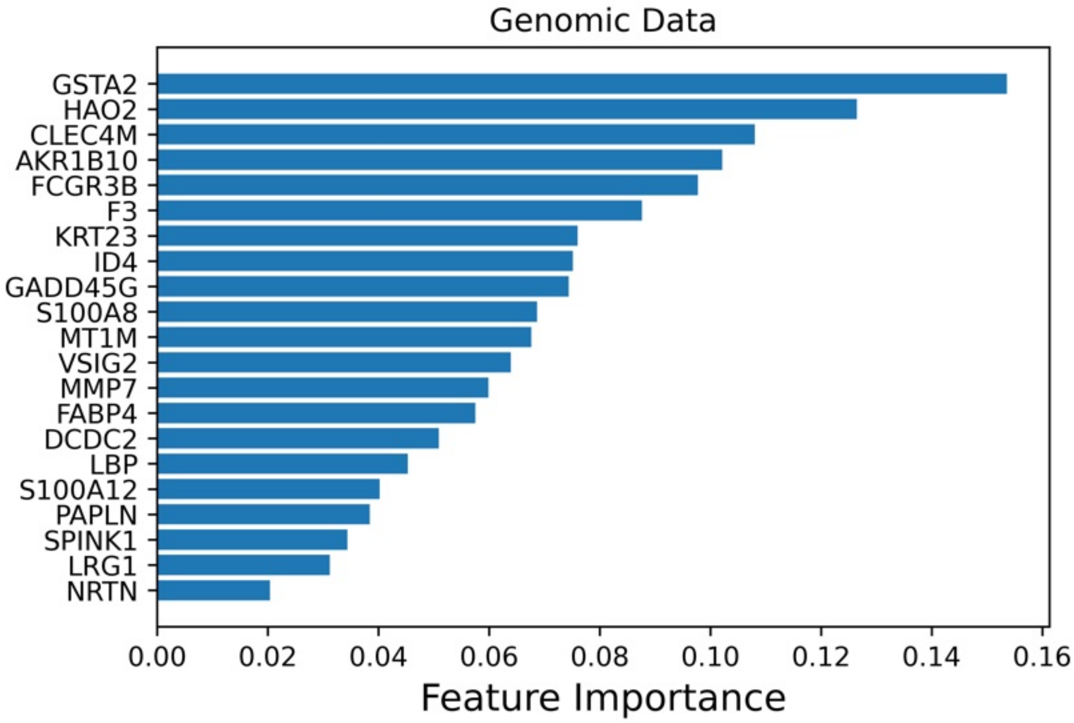

Figure AI in S1 Text: Top ranked genes for integrated analysis of Liver 3-Way.

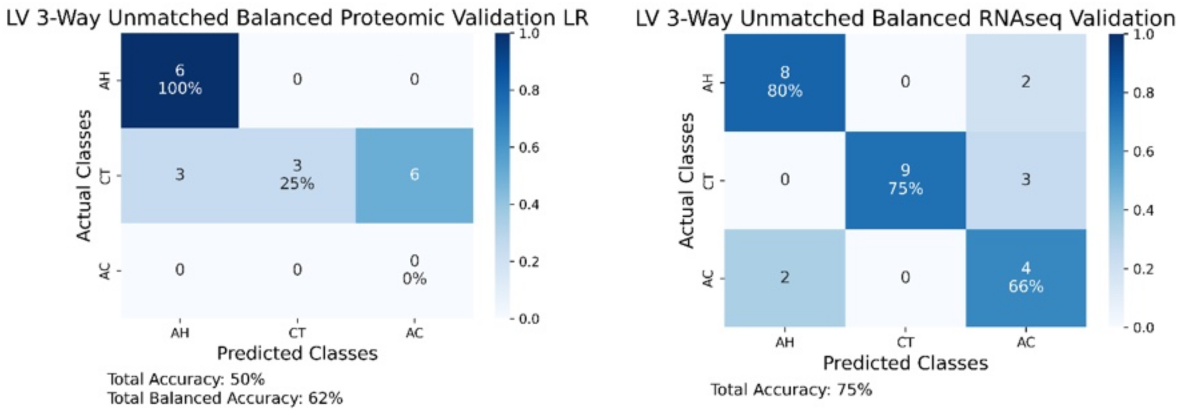

Figure AJ in S1 Text: Confusion matrices for validation of Liver 3-Way Unmatched Balanced transcriptomic and proteomic models in independent validation data.

f. Integrated Analysis of PBMC 3-Way (AH vs Healthy vs AC)

Table AD in S1 Text: Some of the best performing transcriptomic and proteomic configurations from PBMC 3-Way Unmatched Balanced datasets were paired together for integrated analysis. The best performing pair is highlighted in green. Columns are proteomic pipeline configurations, rows are transcriptomic pipeline configurations.

	LR DE Variance 2.5 70 FS Test 5 (33) (17/7/0)	LR DE None 25 FS Test 4 (20) (17/4/0)	SVM DE Variance 2.5 70 FS Test 5 (33) (17/7/0)	SVM DE None 25 FS Test 4 (20) (17/4/0)
LR Hybrid DE 3 25 FS Test 4 (16) (16/3/4)	0.70	0.74 (RNA:0.74, Prot: 0.66)	0.81 (RNA: 0.74, Prot: 0.77)	0.81 (RNA: 0.74, Prot: 0.74)
LR Union IG 3.5 100 FS Test 5 (16-17) (28/5/1)	0.70	0.70	0.70	0.70
LR Hybrid DE 3 100 FS Test 5 (39) (25/8/11)	0.70	0.70	0.70	0.70
SVM Hybrid DE 3 25 FS Test 4 (16) (16/3/4)	0.70	0.70	0.74	0.70
SVM Union IG 3.5 100 FS Test 5 (16-17) (28/5/1)	0.70	0.70	0.70	0.70
SVM Hybrid DE 3 100 FS Test 5 (39) (25/8/11)	0.70	0.70	0.74	0.70

ROC Curve of Best Integrated Protein and Gene Set for PBMCs:

The receiver operating characteristic curves were generated using one-vs-rest micro-averaging scheme as described in scikit-learn package: scikit-learn.org/stable/auto_examples/model_selection/plot_roc. The classes were not weighted by sample size.

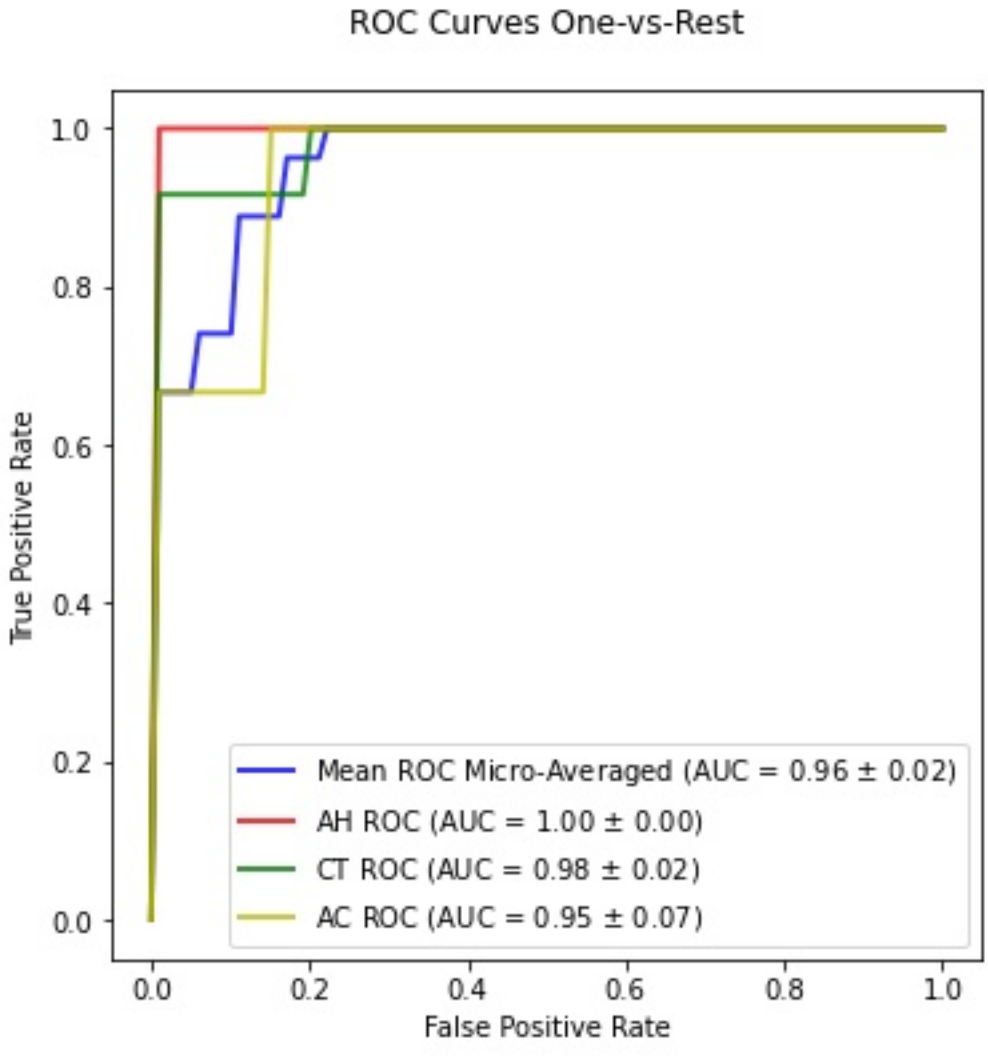

Figure AK in S1 Text: The receiver operating characteristic curve of the best integrated gene-protein PBMC 3-Way model.

Per-class Sensitivities and Specificities of Best Integrated Protein and Gene Set:

AH sensitivity = 1

AH specificity = 1

CT sensitivity = 0.92

CT specificity = 0.73

AC sensitivity = 0.33

AC specificity = 0.95

ROC Curve of Best Gene Set for PBMCs:

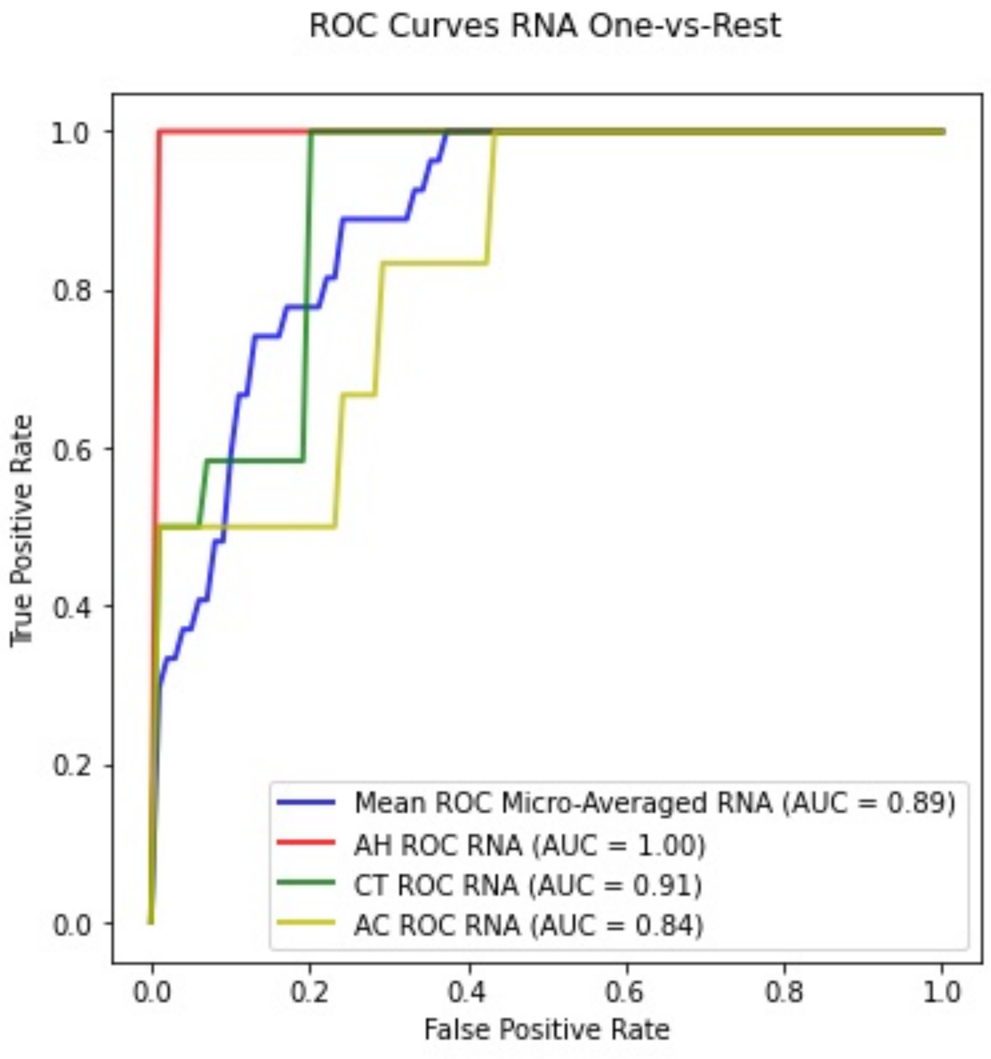

Figure AL in S1 Text: The receiver operating characteristic curve of the best transcriptomic PBMC 3-Way model.

Per-class Sensitivities and Specificities of Best Gene Set:

AH sensitivity = 0.88

AH specificity = 1

CT sensitivity = 0.58

CT specificity = 0.93

AC sensitivity = 0.83

AC specificity = 0.71

ROC Curve of Best Protein Set for PBMCs:

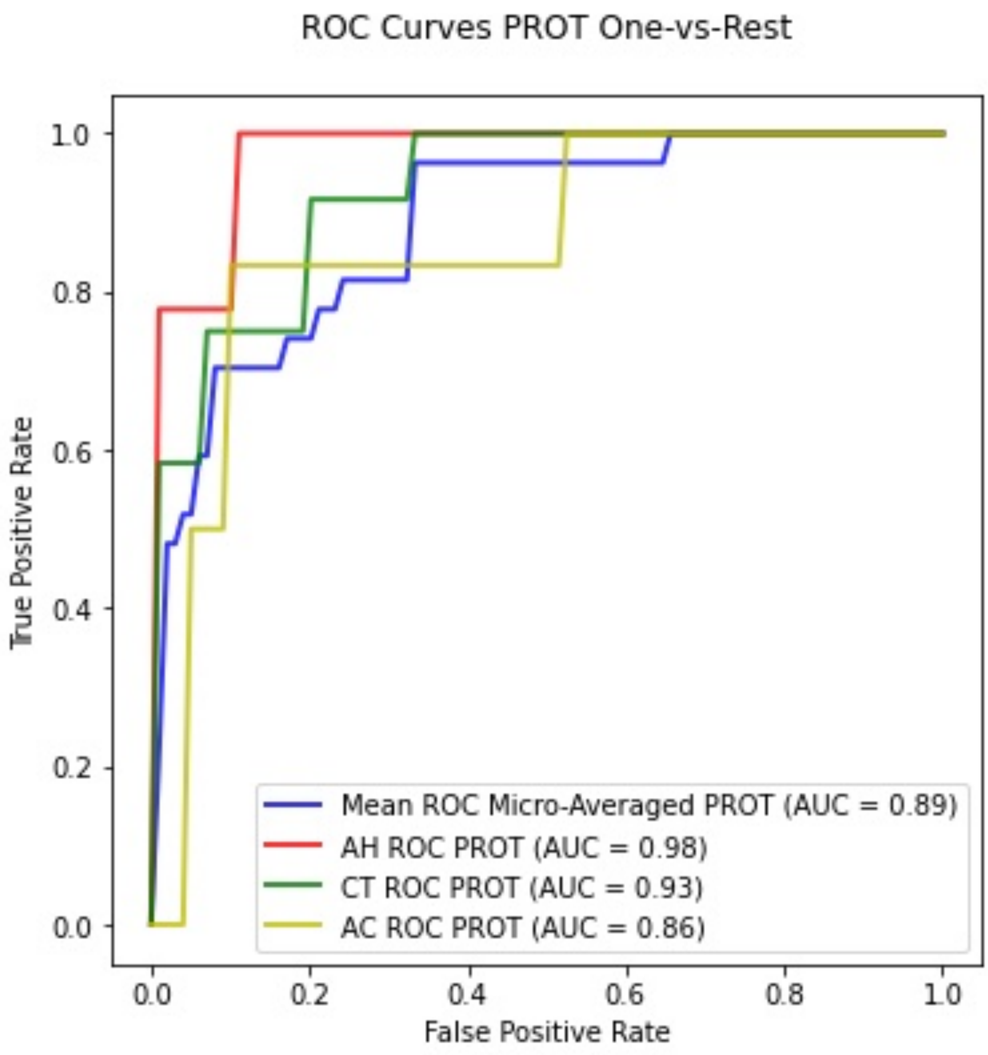

Figure AM in S1 Text: The receiver operating characteristic curve of the best proteomic PBMC 3-Way model.

Per-class Sensitivities and Specificities of Best Protein Set:

AH sensitivity = 0.77

AH specificity = 1

CT sensitivity = 0.75

CT specificity = 0.93

AC sensitivity = 0.83

AC specificity = 0.76

Best protein set for PBMC 3-Way Integrated Analysis:

ACTN1_HUMAN, ALBU_HUMAN, CCL5_HUMAN, CXCL7_HUMAN, FHL1_HUMAN, FIBA_HUMAN, FIBB_HUMAN, FIBG_HUMAN, FRIL_HUMAN, FSTL1_HUMAN, GP1BB_HUMAN, ILK_HUMAN, ITA2B_HUMAN, ITB1_HUMAN, ITB3_HUMAN, LIMS1_HUMAN, LYSC_HUMAN, MYL9_HUMAN, PP14A_HUMAN, RAP1A_HUMAN, RS4Y1_HUMAN, SBP1_HUMAN, SDPR_HUMAN, TBA4A_HUMAN, TBA8_HUMAN, TBB1_HUMAN, TPM2_HUMAN, TRML1_HUMAN, TSN15_HUMAN, TSP1_HUMAN, URP2_HUMAN, VINC_HUMAN, VTDB_HUMAN – 33 proteins.

Splitting best proteins by their differential expressions status in each pairwise comparison of corresponding unmatched balanced dataset:

All proteins are only differentially expressed for AH vs CT pairwise comparison. This is due to the two other pairwise comparison not having enough statistical power (AH vs AC too similar, AC vs CT too few samples).

Table AE in S1 Text: Top 20 AGOTOOL hits for AH vs CT pairwise comparison of PBMC 3-Way Integrated dataset. The pathways were sorted by adjusted p-value. The AH vs AC and AC vs CT pairwise comparison had too few samples to perform pathway analysis.

AH vs CT
platelet activation
phagosome
tight junction
ECM-receptor interaction
focal adhesion
regulation of actin cytoskeleton
leukocyte transendothelial migration
pathogenic Escherichia coli infection
primary focal segmental glomerulosclerosis (FSGS)
focal adhesion
VEGFA-VEGFR2 signaling pathway
complement system
integrin-mediated cell adhesion
COVID-19, thrombosis and anticoagulation
platelet aggregation
positive regulation of substrate adhesion-dependent cell spreading
cell adhesion
cell-cell adhesion
cell activation
regulation of body fluid levels

Protein ranking by model coefficients for PBMC 3-Way Integrated dataset:

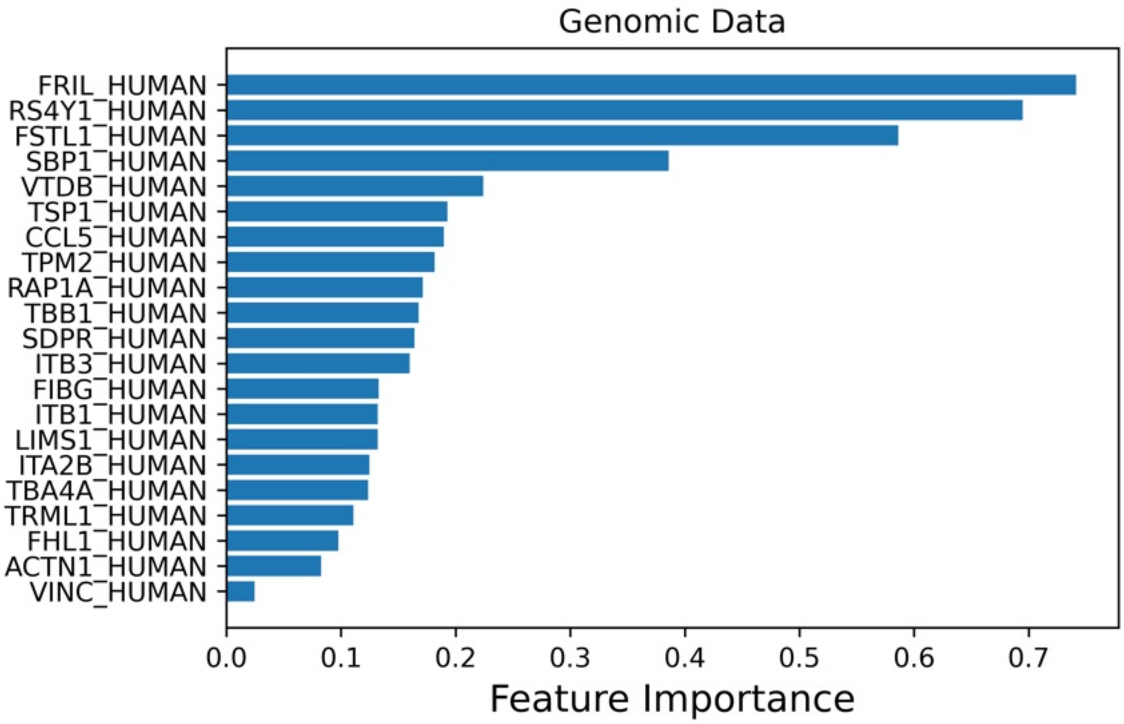

Figure AN in S1 Text: Top ranked proteins for integrated analysis of PBMC 3-Way.

Best gene set for PBMC 3-Way Integrated Analysis:

'AHSP', ALAS2', CA1', CD177', CDK10', EHMT1', HBD', HBM', IFI27', IL1R2', MECP2', MMP8', MMP9', SELENBP1', SLC4A1', TANGO2 – 16 genes.

Splitting best genes by their differential expressions status in each pairwise comparison of corresponding unmatched balanced dataset:

AH vs CT: AHSP, ALAS2, CA1, CD177, CDK10, HBD, IFI27, IL1R2, MMP8, MMP9, SELENBP1, SLC4A1, TANGO2.

AH vs AC: AHSP, CA1, CD177, CDK10, EHMT1, HBD, HBM, IFI27, IL1R2, MECP2, MMP8, MMP9, SELENBP1, SLC4A1.

CT vs AC: AHSP, ALAS2, CA1, EHMT1, HBD, HBM, IFI27, MECP2, SELENBP1, SLC4A1, TANGO2.

Table AF in S1 Text: Top 20 Enrichr hits per each pairwise comparison of PBMC 3-Way Integrated dataset. The pathways were sorted by adjusted p-value.

AH vs CT	AH vs AC	CT vs AC
oxygen/carbon dioxide exchange in erythrocytes	oxygen/carbon dioxide exchange in erythrocytes	oxygen/carbon dioxide exchange in erythrocytes
alpha-hemoglobin stabilizing enzyme	IL1 and megakaryocytes in obesity	alpha-hemoglobin stabilizing enzyme
IL1 and megakaryocytes in obesity	matrix metalloproteinases	erythrocyte differentiation
matrix Metalloproteinases	lung fibrosis	bicarbonate transport
endodermal cell differentiation	endodermal cell differentiation	myeloid cell differentiation
erythrocyte differentiation	endoderm formation	cytosine methylation
endoderm formation	bicarbonate transport	heme biosynthesis
bicarbonate transport	extracellular matrix organization	organic anion transport
myeloid cell differentiation	cytokine-mediated signaling pathway	oxygen homeostasis
cytokine-mediated signaling pathway	cytosine methylation	regulation of microtubule nucleation
extracellular matrix organization	mammary gland development pathway – involution	ion homeostasis
extracellular matrix disassembly	osteopontin signaling	cellular ion homeostasis
organic anion transport	extracellular matrix disassembly	gas homeostasis
neutrophil degranulation	organic anion transport	regulation of DNA methylation
neutrophil activation involved in immune response	neutrophil degranulation	peptidyl-lysine monomethylation
neutrophil mediated immunity	neutrophil activation involved in immune response	heme biosynthesis
positive regulation of receptor binding	positive regulation of receptor binding	bicarbonate transporters
neutrophil extravasation	neutrophil extravasation	reversible hydration of carbon dioxide
oxygen homeostasis	positive regulation of keratinocyte migration	porphyrin metabolism
positive regulation of keratinocyte migration	regulation of microtubule nucleation	mechanisms of transcriptional repression by DNA methylation

Gene ranking by model coefficients for PBMC 3-Way Integrated dataset:

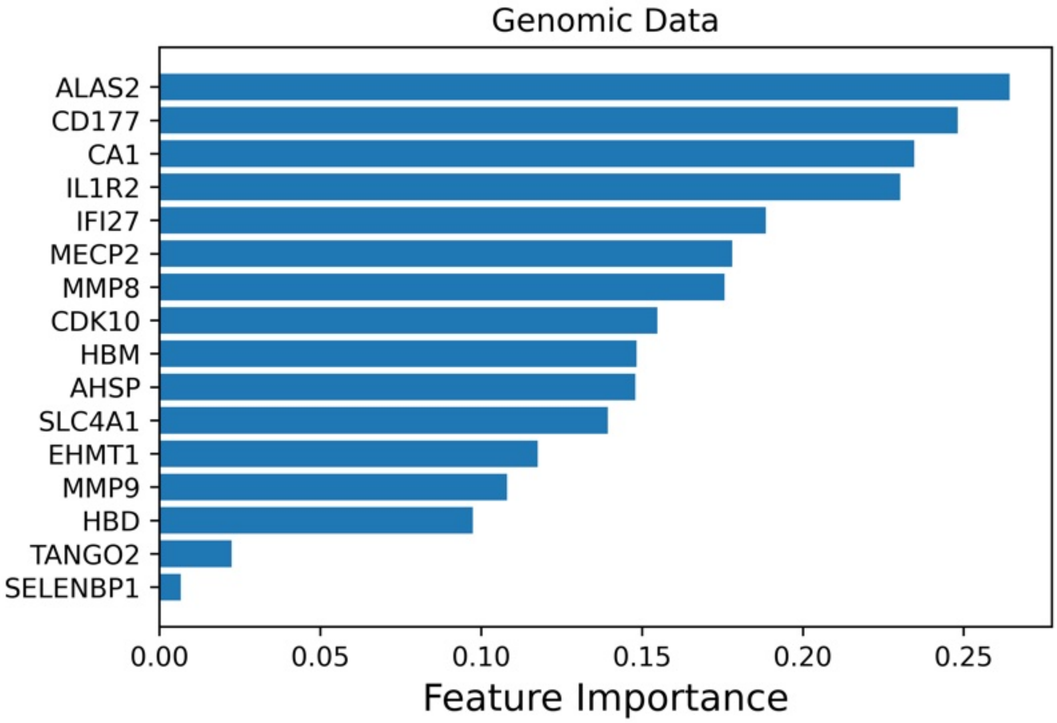

Figure AO in S1 Text: Top ranked genes for integrated analysis of PBMC 3-Way.

g. Intersection Analysis of LV 3-Way Matched Balanced Integrated (AH vs Healthy vs AC)

There are G = 1304 significant DEGs. There are P = 2647 significant DEPs.

Assume overlap signifies the ov = 374 overlapping DEGs/DEPs.

What is the probability of n element(s) in common (between 59 DEGs and 19 DEPs)?

$$P(of\ m\ best\ genes\ in\ overlap) = ((G - ov)\ /\ G)^{(g-m)} * (ov\ /\ G)^{m}\ *\ C(g, m)$$

$$for(i = 0; i \leq g; i++): P(of\ i\ best\ genes\ in\ overlap)\ *\ ((P - i)/P)^{(p-n)}\ *\ (i/P)^{n}\ *\ C(p, n)$$

The calculation above was written in Python.

Probability of 0 elements in common between best genes and proteins ≈ 88.5%

Probability of 1 elements in common between best genes and proteins ≈ 10.8%

Probability of 2 elements in common between best genes and proteins ≈ 0.65%

Assume probability of ≥ 3 elements in common is negligible.

Expected value ≈ 0.12

h. Intersection Analysis of PBMC 3-Way Matched Balanced Integrated (AH vs Healthy vs AC)

There are 971 SDE genes. There are 876 SDE proteins. The overlap between DEGs and DEPs is 88.

What is the probability of n element(s) in common (between 16 DEGs and 33 DEPs)?

Probability of 0 elements in common between best genes and proteins ≈ 94.7%

Probability of 1 elements in common between best genes and proteins ≈ 5%

Assume probability of ≥ 2 elements in common is negligible.

Expected value ≈ 0.05

SUPPLEMENTARY REFERENCES

1. Listopad S, Magnan C, Asghar A, Stolz A, Tayek JA, Liu Z, et al. Differentiating between liver diseases by applying multiclass machine learning approaches to transcriptomics of liver tissue or blood based samples. JHEP Reports. 2022;4(10).

2. Massey V, Parrish A, Argemi J, Moreno M, Mello A, García-Rocha M, et al. Integrated Multiomics Reveals Glucose Use Reprogramming and Identifies a Novel Hexokinase in Alcoholic Hepatitis. Gastroenterology. 2021;160(5):1725-1740.

3. Hardesty J, Day L, Warner J, Warner D, Gritsenko M, Asghar A, et al. Hepatic protein and phosphoprotein signatures of alcohol-associated cirrhosis and hepatitis. The American Journal of Pathology. 2022;192(7):1066-1082.

4. Argemi J, Kedia K, Gritsenko M, Clemente-Sanchez A, Asghar A, Herranz J, et al. Integrated transcriptomic and proteomic analysis identifies plasma biomarkers of hepatocellular failure in alcohol-associated hepatitis. The American Journal of Pathology. 2022; 192(12): 1658-1669.

5. Dobin A, Davis C, Schlesinger F, Drenkow J, Zaleski C, Jha S, et al. STAR: ultrafast universal RNA-seq aligner. *Bioinformatics*. 2013;29(1):15-21.

6. Kampf C, Mardinoglu A, Fagerberg L, Hallstrom BM, Edlund K, Lundberg E, et al. The human liver-specific proteome defined by transcriptomics and antibody-based profiling. Faseb Journal 2014;28:2901-2914.

7. Trapnell C, Roberts A, Goff L, Pertea G, Kim D, Kelley DR, et al. Differential gene and transcript expression analysis of RNA-seq experiments with TopHat and Cufflinks. Nature Protocols 2012;7:562-578.

8. Polpitiya AD, Qian W, Jaitly N, Petyuk VA, Adkins JN, Camp DG, et al. DAnTE: a statistical tool for quantitative analysis of -omics data. Bioinformatics. 2008; 24(13):1556-8.

9. Chen EY, Tan CM, Kou Y, Duan QN, Wang ZC, Meirelles GV, et al. Enrichr: interactive and collaborative HTML5 gene list enrichment analysis tool. Bmc Bioinformatics 2013;14.

10. Schölz C, Lyon D, Refsgaard JC, Jensen LJ, Choudhary C, Weinert BT. Avoiding abundance bias in the functional annotation of post-translationally modified proteins. Nat Methods. 2015;12(11):1003-4.
